# Diagnostic accuracy, treatment and prognosis of myocardial infarction: an 11-year follow-up of a community-based cohort of 0.5 million Chinese adults

**DOI:** 10.1136/bmjph-2025-004019

**Published:** 2026-03-10

**Authors:** Iain James Turnbull, Robert Clarke, Neil Wright, Dianjianyi Sun, Christiana Kartsonaki, Pei Pei, Canqing Yu, Bing Han, Ling Yang, Daniel Avery, Maxim Barnard, Jun Lv, Liming Li, Yiping Chen, Zhengming Chen, on behalf of the China Kadoorie Biobank Collaborative Group, Junshi Chen

**Affiliations:** 1 Nuffield Department of Population Health, MRC Clinical Trial Service Unit and Epidemiological Studies Unit, Oxford, UK; 2 Nuffield Department of Population Health, University of Oxford, Oxford, UK; 3 Department of Epidemiology & Biostatistics, Peking University, Beijing, China; 4 Peking University Center for Public Health and Epidemic Preparedness and Response, Beijing, China; 5 Ministry of Education, Beijing, China; 6 Peking University School of Public Health Department of Epidemiology and Biostatistics, Beijing, China; 7 Epidemiology & Biostatistics, Peking University Health Science Center, Beijing, China; 8 NCDs Prevention and Control Department, Henan CDC, Henan, China; 9 Clinical Trial Service Unit and Epidemiological Studies Unit, University of Oxford, Oxford, UK; 10 Ministry of Education, Brasilia, Brazil; 11 School of Public Health, Peking University Health Science Center, Beijing, China

**Keywords:** Epidemiology, Preventive Medicine, Public Health

## Abstract

**Introduction:**

Myocardial infarction (MI) is a major cause of premature death and disability in China, but available data on diagnostic accuracy, treatment and prognosis of MI cases remain limited.

**Methods:**

The China Kadoorie Biobank enrolled 512 000 adults (mean age 51 years, 59% women) from 10 (five urban, five rural) areas between 2004 and 2008. Medical records were sought on 37 159 reported first incident ischaemic heart disease (IHD) cases occurring before 2018 for adjudication. Diagnostic accuracy and risks of recurrent MI, stroke, heart failure and all-cause mortality were assessed for reported and adjudicated cases of MI and all IHD.

**Results:**

Among 19 135 adjudicated IHD cases, 10.2% (n=1948) had MI, with diagnostic accuracy of 98% for MI and 93% for all IHD. Use of guideline-directed (antiplatelet, lipid-lowering, antihypertensive and anticoagulant) medications in the hospital was high (93% MI; 83% all IHD), but differed by hospital tier and area. Use of coronary revascularisation was low (39% MI; 10% all IHD), even in higher tier hospitals. The 28-day case-fatality was sixfold greater for MI than all IHD (12.0% vs 2.0%) and higher in older cases and in women, residents in rural areas, but comparable by hospital tier. Disparities in 5-year mortality rates persisted for both MI and all IHD, by age, sex, area and hospital tiers.

**Conclusions:**

This large community-based prospective study demonstrated high levels of diagnostic accuracy for MI and all IHD in Chinese adults, but also identified disparities in treatment and outcomes that should be prioritised in future prevention strategies.

**WHAT IS ALREADY KNOWN ON THIS TOPIC**

- Ischaemic heart disease (IHD) is the leading cause of premature death in China, but most of the available evidence on diagnosis, treatment and prognosis of myocardial infarction (MI) has been restricted to cases treated in higher-tier hospitals with a short duration of follow-up.

**WHAT THIS STUDY ADDS**

- In an 11-year follow-up of the community-based China Kadoorie Biobank of 512 000 adults, medical records on 19 135 first incident IHD cases, identified between 2004 and 2017, were retrieved and independently adjudicated. The diagnostic accuracy was 98% for MI cases and 93% for all IHD cases, with 10% of adjudicated IHD cases having MI. Use of guideline-directed medications in hospital was high (93% MI; 83% all IHD), but use of any coronary revascularisation was low (39% MI; 10% all IHD). The 28-day case fatality rates were sixfold greater for MI than for all IHD, and these differences persisted for MI recurrence and all-cause mortality at 5 years, with worse outcomes in older cases, women, residents in rural areas and cases treated in lower-tier hospitals.

**HOW THIS STUDY MIGHT AFFECT RESEARCH PRACTICE OR POLICY**

- The findings highlight substantial disparities in short- and long-term disease outcomes between MI and all IHD cases and by age, sex, area and hospital tier in China, highlighting priorities for future cardiovascular disease prevention strategies.

## Introduction

 Worldwide, ischaemic heart disease (IHD) accounted for ~9 million deaths in 2019,^1^ including ~2 million in China.^2^ While age-standardised IHD incidence and mortality have declined worldwide,^1^ they have increased in China, reflecting improved survival and population ageing.^3^ China now accounts for about two-fifths of recent global increases in IHD deaths.^4^ IHD is typically classified into myocardial infarction (MI) (acute coronary occlusion and elevated troponins) and non-MI IHD (stable or unstable atherosclerotic plaques without acute arterial occlusion and normal troponin levels).^5^

Early MI treatment—including thrombolysis, coronary revascularisation and guideline-directed medications (antiplatelet, blood pressure-lowering, low-density lipoprotein cholesterol-lowering and anticoagulant medications)—has significantly improved survival.^6^,^8^ In China, hospital registries in higher-tier centres^9^ or short-term prospective studies^10^ have reported revascularisation and medication use, but few community-based studies have examined diagnostic accuracy, treatment patterns and risks of recurrence or mortality for MI across hospital types. Previous studies documented disparities in hospital treatment and case-fatality rates (CFRs) by geographic area,^11^ sex^12^ and hospital tier,^13^ yet population-level data on prognosis after MI remain scarce.^14^,^20^

This study evaluated the natural history of MI and all IHD cases over an 11-year follow-up in the China Kadoorie Biobank (CKB), a community-based cohort of 0.5 million adults from 10 regions during 2004–2017.^21^ The aims were to: (1) compare diagnostic accuracy of reported and adjudicated MI and all IHD cases; (2) assess the use of guideline-directed medications and coronary artery revascularisation in adjudicated first incident IHD cases and (3) report rates of MI recurrence, stroke, hospitalised heart failure (HF) and all-cause mortality—by MI and all IHD cases and by subgroups of age, area, sex and hospital tier.

## Methods

### Study population

Details of the CKB study design, methods and participants of the CKB have been previously reported.^21^ Briefly, 512 726 men and women, aged 30–79 years, were recruited from 10 diverse regions (five urban and five rural) during 2004–2008 into a community-based cohort study. At baseline, participants completed an interview-administered questionnaire on demographic, socioeconomic, medical and lifestyle factors; underwent measurements of blood pressure, height, weight and lung function and provided a blood sample for long-term storage.

### Follow-up for fatal and non-fatal IHD and other outcomes

After baseline, follow-up for non-fatal and fatal IHD cases used electronic linkage, via unique national IDs, with the nationwide health insurance (HI) system (>97% coverage at enrolment) for all hospitalisation episodes and death and disease registers. Active follow-up was conducted annually using residential records and local administrators were asked to identify events not captured by death or disease registers or HI, including participants who were not insured by HI agencies. In China, the Ministry of Health classified hospitals based on the numbers of patient beds and delivery of specialist care as: (1) tier 1 hospitals (<100 beds with provision of basic and preventive services); (2) tier 2 hospitals (101–500 beds with provision of some specialist care) and (3) tier 3 hospitals (>500 beds with provision of highly specialised services). Causes of death were identified from death registers and supplemented by medical records; for <5% of deaths without recent medical attention, verbal autopsy was used.^22^ This report involved first-incident hospitalised IHD cases reported between 25 June 2004 and 31 December 2017. All reported IHD cases were coded by trained medical staff, blinded to personal information, using the International Classification of Diseases, 10th revision (ICD-10). Adjudicated cases were additionally reviewed centrally by a cardiology-trained research clinician. By 1 January 2018 (censoring date for the present analyses), 45 937 (9.2%) participants had died and 5121 (1%) were lost to follow-up, after excluding those with prior self-reported IHD.

### First incident and subsequent IHD cases

All first-incident IHD subtypes were classified by WHO criteria: MI (ICD-10: I21–I23) and all IHD (I20-I25), including MI, angina pectoris (I20), other acute non-MI IHD (I24) and chronic IHD (I25) (online supplemental table S1). For prognosis, primary outcomes included recurrent MI, stroke, hospitalised HF and all-cause mortality after a first incident IHD event. Outcomes were: any hospitalised MI (I21–I23); stroke (I60–I61, I63–I64); HF (I50); or death from any cause. Additional details of diagnostic criteria and adjudication procedures are provided in the online supplemental methods.

Participants with self-reported doctor-diagnosed prior IHD (n=7200) at baseline were excluded. Since medical records were rarely available for IHD cases reported only to death registers, 4045 such cases were excluded from diagnostic accuracy analyses. However, ~7% of reported IHD cases, identified by disease registers or the HI system, were fatal and included in subsequent analyses.

### Statistical analysis

Analyses estimated retrieval rates and reporting and diagnostic accuracy of IHD cases (using positive predictive values (PPVs)), based on non-clinical verification of hospital records and online adjudication by Chinese cardiologists, by MI and all IHD. Cox proportional hazards models compared the strength of associations of MI cases with usual systolic blood pressure (SBP) or baseline body mass index (BMI) to validate adjudication procedures.

The 28-day CFRs for adjudicated cases of MI and all IHD cases were estimated as proportions who died within 28 days of the index event and standardised to the age distribution (10-year groups) of all IHD cases. CIs for age-specific rates were exact, and for age-standardised rates were Wald CIs on the log odds scale.^23^ Cumulative mortality rates following first incident IHD were estimated as 1–Kaplan-Meier survival probability. Cumulative event rates for recurrent MI and other CVD outcomes were estimated using a cumulative incidence function, treating death as a competing risk (cmprsk package in R), with 95% CIs based on the log-minus-log transformation.^24 25^ Event rates were estimated for all participants with a first incident IHD event and separately for MI. Age-specific rates were calculated by sex, area (urban/rural) and hospital tier. In analyses of multiple events, different event types were treated as competing risks. Unless otherwise specified, cumulative event rates for recurrent MI, stroke, HF and death were estimated from those occurring after 28 days following a first IHD event. Baseline characteristics of participants with and without IHD were presented after standardisation by sex, age (5-year groups) and area. All analyses were conducted in R (V.4.2.2).

### Public and patient involvement

Prior to enrolment in the study, potentially eligible participants at each of the 10 centres were invited to attend information meetings with representatives of study principal investigators, regional public health leaders and local government officials. These meetings explained the aims and procedures of the study and what participation involved and provided opportunities for questions.

## Results

### Distribution of included cases

**Figure 1:**
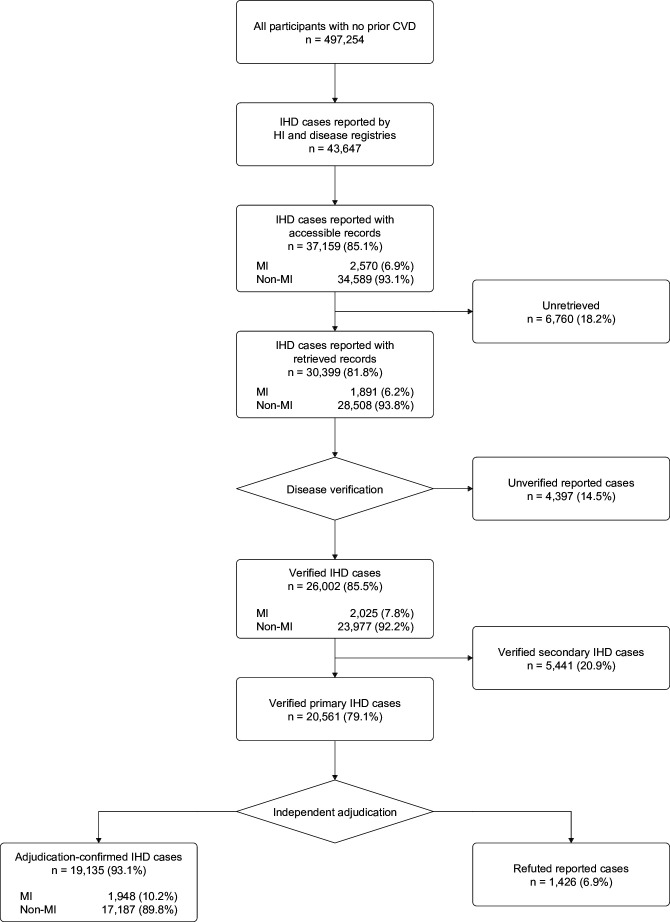
Flow chart of verification and adjudication of all reported IHD cases. The flow chart summarises the verification and adjudication of reported IHD cases. Participants with a history of IHD at baseline were excluded, as were IHD cases reported by death registries or with inaccessible records or inconsistent disease status. 7200 IHD cases with prior IHD at baseline and 4045 IHD cases reported by death registries were excluded. 6488 reported IHD cases that were inaccessible and/or had inconsistent status were excluded. CVD, cardiovascular disease; HI, health insurance; IHD, ischaemic heart disease; MI, myocardial infarction

Overall, during 5 326 454 person-years of follow-up for incident IHD, 54 892 reported non-fatal or fatal IHD cases were identified. Of 43 647 eligible first-ever IHD cases, 6488 (14.9%) from inaccessible hospitals or with inconsistent adjudication were excluded (figure 1). Among 37 159 accessible cases, medical records were retrieved on 30 399 (81.8%) from 412 hospitals (figure 1; table 1). Of these, 26 002 cases (85.5%) were verified against the primary diagnosis of the discharge summary retrieved by local research staff while 4397 (14.5%) were unverified. Of all verified cases, 20 561 (79.1%) were primary IHD cases, that is, where IHD was the main condition treated during admission, and 5441 (20.9%) were secondary cases. Among these cases, 137 (2.5%) were MI. Of primary verified cases, 19 135 (93.1%) were confirmed by clinical adjudication as IHD cases—of which 1948 (10.2%) had MI—and 1426 (6.9%), with no clinical evidence of IHD, were refuted (figure 1).

**Table 1:** Reporting and diagnostic accuracy of hospitalised IHD cases by reported IHD subtypes

	No. of reported cases*	Retrieved cases		Verified cases		Adjudication-confirmed cases	
		No.	%	No.	Reporting accuracy (PPV, 95% CI)	No.	Diagnostic accuracy (PPV, 95% CI)†
MI	2570	1891	73.6	1689	89.3 (87.9 to 90.7)	1482	97.7 (96.9 to 98.4)
All IHD	37 159	30 399	81.8	26 002	85.5 (85.1 to 85.9)	19 135	93.1 (92.7 to 93.4)

* Refers to reported IHD cases with accessible records.

† Cases with secondary diagnosis of IHD excluded from diagnostic accuracy assessment.

IHD, ischaemic heart disease; MI, myocardial infarction; PPV, positive predictive value.

### Baseline characteristics

At baseline, reported IHD cases had a mean age of 59.0 (SD 9.9) years, 59% were women and 51% lived in rural areas (online supplemental tables S2–S5). In contrast, MI cases were older (mean 60.3 (9.8) years) and more often male (65%) and rural (57%). MI cases had a higher prevalence of prior disease (3% stroke/transient ischaemic attack; 14% diabetes), and higher mean levels of SBP (138.1 (24.1) mm Hg) than all IHD cases (135.0 (23.3) mm Hg). Self-reported medication use at enrolment was low, in MI and all IHD cases (for all IHD: 0.3% statins, 15% antihypertensive agents and 1% aspirin). In total, 29 631 (79.7%) IHD cases were reported by HI agencies, with proportions increasing in later years of follow-up, and 46.9% were treated in tier 3 hospitals (online supplemental table S6). Baseline characteristics are provided after standardisation for age by access and retrieval of medical records (online supplemental table S3), adjudication status (online supplemental table S4) and hospitalisation of fatal cases (online supplemental table S5) (and hence, only in ‘All participants’ in online supplemental table S2 and ‘Reported cases’ in online supplemental table S4 do percentages in columns match the raw percentages). Compared with those in accessible hospitals, IHD cases in inaccessible hospitals were younger, more frequent in rural areas and had lower levels of SBP at baseline (online supplemental table S3). While reported IHD cases were more frequently resident in rural areas, the baseline characteristics of reported, verified and adjudication-confirmed IHD cases were broadly comparable (online supplemental table S4). Likewise, characteristics for fatal out-of-hospital IHD cases were similar to those for fatal hospitalised cases (online supplemental table S5).

### Reporting and diagnostic accuracy

Overall, the PPV for reported MI cases was 97.7 (95% CI 96.9 to 98.4) and was 85.5% (85.1–85.9) for all IHD cases (table 1). Following the exclusion of secondary IHD cases, which lacked sufficient diagnostic information, 20 561 primary cases were available for adjudication (figure 1). Of these, 19 135 cases met WHO diagnostic criteria for MI or IHD, yielding an overall PPV for adjudication of 97.7% (96.9–98.4) for MI and 93.1% (92.7–93.4) for all IHD (table 1). Adjudication increased the proportion of MI cases by nearly 50% (1948 (10.2%) adjudicated vs 2570 (6.9%) reported MI cases (figure 1). Analyses of diagnostic accuracy are provided by baseline characteristics and year for all IHD (online supplemental table S6), year for MI (online supplemental table S7), validation status of MI and non-MI IHD (online supplemental table S8) and area for all IHD (online supplemental table S9). Additional details are provided for unverified IHD cases (online supplemental tables S10 and S11), refuted cases (online supplemental tables S12 and 13) and reporting accuracy by source of cases (online supplemental table S14: online supplemental methods).

The strengths of associations of usual SBP or baseline BMI with MI were slightly greater for adjudicated than verified or reported cases (online supplemental figure S1), with weaker associations with non-MI IHD (online supplemental figure S2) providing support for the validity of the adjudication procedures.

### In-hospital management of MI and all IHD cases

Among 19 135 adjudication-confirmed hospitalised cases, 1948 (10.2%) had MI and 17 187 (89.8%) had non-MI IHD (figure 1, online supplemental tables S14–S19). Of MI cases, most were managed in tier 3 hospitals (n=1790; 73.5%; online supplemental table S18). Cardiac biomarkers (typically troponins) were measured in 12 800 (66.9%) IHD cases, with higher use in MI (94.5%), men than women (72.2% vs 63.3%), urban than rural residents (71.5% vs 59.0%) and in tier 3 vs tier 2 vs tier 1 hospitals (77.8% vs 65.1% vs 39.0%, respectively) (online supplemental table S15). Biomarker use increased during follow-up (55.2% for 2008 vs 72.1% for 2015), but almost all cases had ECG (97.7%: online supplemental table S15).

Among all adjudication-confirmed IHD cases, 66.8%, 59.0%, 62.4% and 19.9% reported current use of antiplatelet, lipid-lowering, antihypertensive and anticoagulant medications, respectively (table 2). Use of guideline-directed medications during hospital stay was higher in MI cases (antiplatelet 86.6%; lipid-lowering 78.9%; antihypertensive 73.5%; anticoagulant 64.1%), whereas use of traditional Chinese medicines (TCMs) was higher in all IHD than MI cases (69.6% vs 58.5%). Men (online supplemental table S16), urban residents (online supplemental table S17) and tier 3 patients (online supplemental table S18) had a higher use of medications for both MI and all IHD cases while TCM use was higher in women (online supplemental table S16), rural residents (online supplemental table S17) and lower-tier hospitals (online supplemental table S18). Patterns of medication use did not differ by calendar year (online supplemental table S19). No data were available on use of medications after discharge from hospital.

**Table 2:** Use of standard medication, revascularisation and traditional Chinese medicine by adjudication-confirmed IHD subtypes

Medications, n (%)	MI (n=1948)	All IHD (n=19 135)
Standard medication		
Antiplatelet*	1686 (86.6)	12 786 (66.8)
Lipid-lowering†	1536 (78.9)	11 286 (59.0)
Antihypertensive‡	1431 (73.5)	11 937 (62.4)
Anticoagulant§	1249 (64.1)	3812 (19.9)
Any standard medication	1804 (92.6)	15 834 (82.7)
Revascularisation		
Thrombolysis	78 (4.0)	103 (0.5)
Any coronary intervention¶	690 (35.4)	1843 (9.6)
Any revascularisation**	752 (38.6)	1929 (10.1)
Traditional Chinese medication	1139 (58.5)	13 312 (69.6)

Combined treatments from first 24 hours after admission, during hospital stay and at discharge.

* Aspirin, clopidogrel or glycoprotein IIb/IIIa antagonist therapy.

† Statin and non-statin lipid-lowering therapy.

‡ ACE inhibitor, angiotensin receptor blocker, beta blocker, calcium antagonist or diuretic therapy.

§ Low molecular weight heparin or unfractionated heparin therapy.

¶ Percutaneous coronary intervention or coronary artery bypass graft.

** Some cases received both thrombolytic therapy and any procedure.

IHD, ischaemic heart disease; MI, myocardial infarction.

Despite high use of medications in hospital, use of coronary revascularisation (thrombolysis, percutaneous coronary intervention or coronary artery bypass graft) was low (10.1% for all IHD; 38.6% MI; table 2), even in higher-tier hospitals (15.0% for all IHD; 44.8% MI, tier 3; online supplemental table S18). Among MI cases, coronary artery revascularisation was more frequent in men (42.9% vs 30.2% in women; online supplemental table S16), urban residents (48.1% vs 22.0% in rural residents; online supplemental table S17) and cases in tier 3 hospitals (44.8% vs 24.4% in tier 2; online supplemental table S18). Annual revascularisation rates in Tier 3 hospitals ranged from 37.6%–46.8% between 2004–2017 with no trends by calendar year (online supplemental table S19). Overall median (IQR) duration of hospital stay was nine (6–13) days for MI and eight (5–12) days for all IHD cases (online supplemental table S2).

### Prognosis of MI and all IHD cases

**Figure 2:**
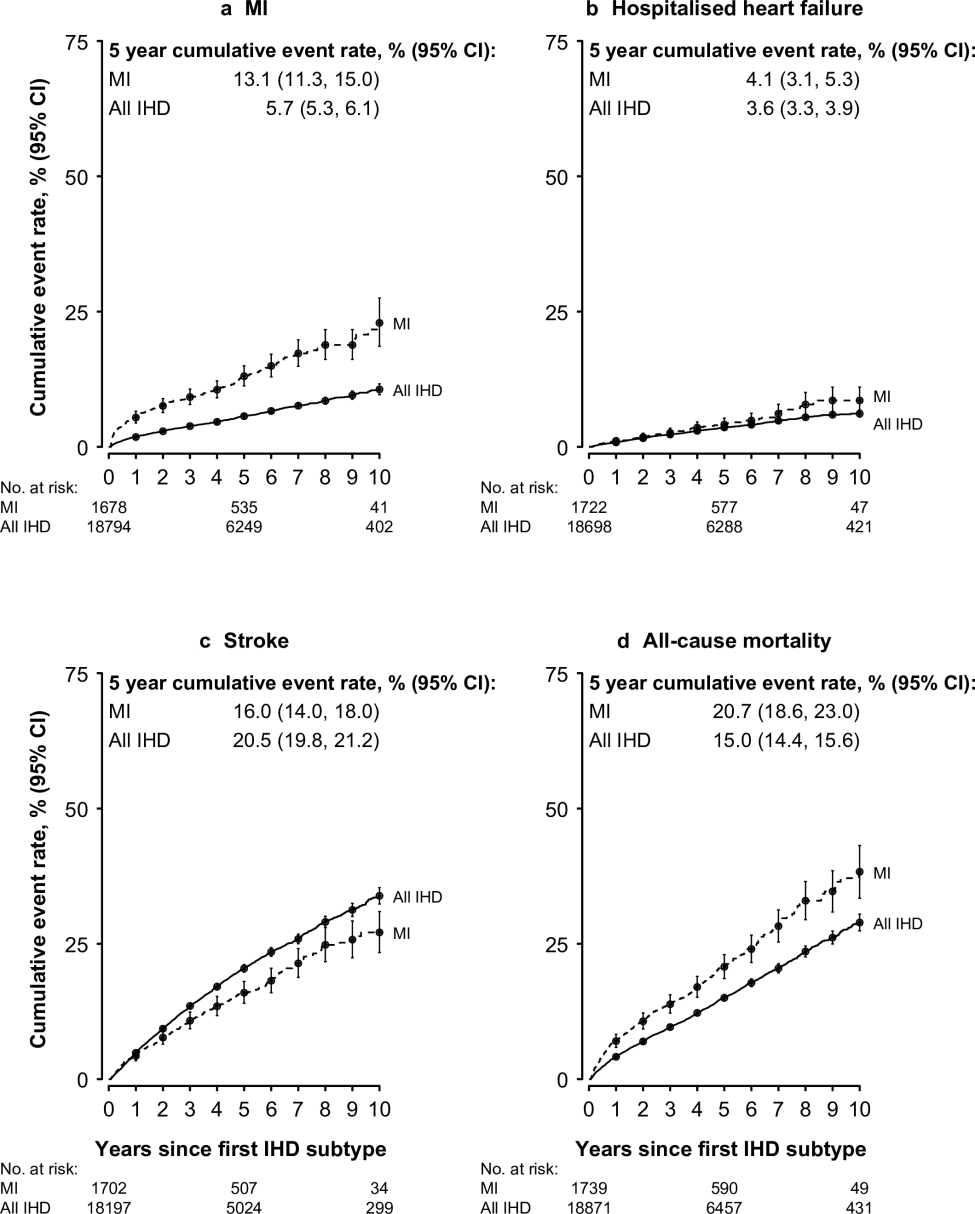
Estimated cumulative event rates of recurrent or subsequent MI events, heart failure, stroke and all−cause mortality from 28 days after first adjudicated events of MI and IHD of any type. The plotted lines show the cumulative incidence function, starting at the date of first IHD event, separately for MI and IHD of any type and years of follow-up. Deaths from any cause were treated as competing risks. Participants experiencing an event or death within 28 days from first IHD event were excluded. Plotted points mark the cumulative incidence function at annual increments. IHD, ischaemic heart disease; MI, myocardial infarction.

**Figure 3:**
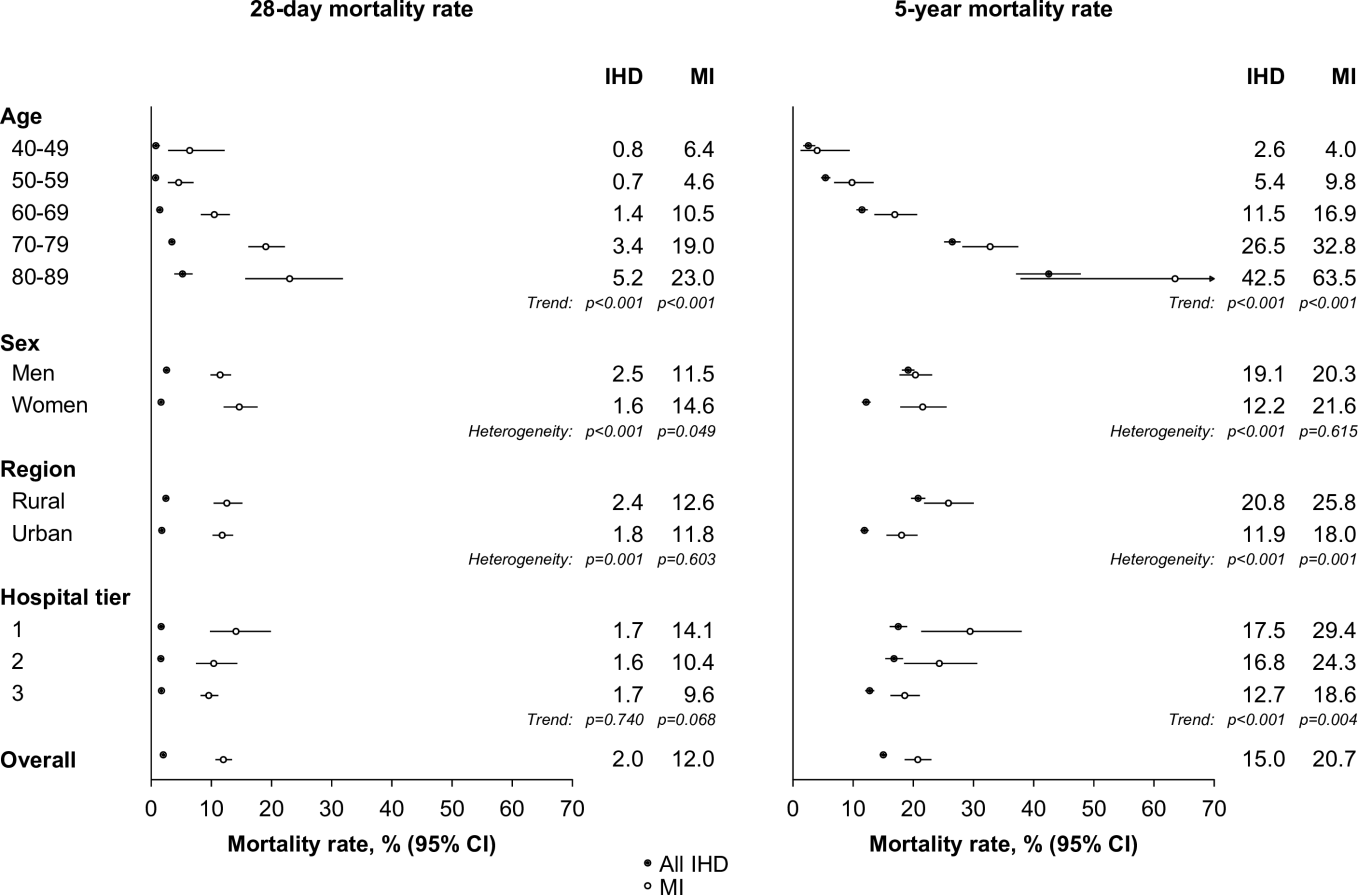
The 28-day and 5-year mortality rates (95% CI) for adjudication-confirmed MI and all IHD cases, by age at risk, sex, area and hospital tier. The 28-day mortality rates for adjudicated cases of MI and all IHD were estimated as proportions who died within 28 days of the index event and standardised to the age distribution (10-year age groups) of all adjudicated IHD cases. The 5-year mortality rates were estimated cumulative event rates, with participants dying within 28 days of the index event excluded. IHD, ischaemic heart disease; MI, myocardial infarction.

The prognosis of MI and all IHD cases is shown in online supplemental tables S20–S22 and figures2  3. Among 19 135 adjudication-confirmed IHD cases, 386 (2.0%, 95% CI 1.8 to 2.2; online supplemental table S21) died within 28 days of onset, with higher age-standardised CFRs in men than women (2.5% vs 1.6%) and rural than urban areas (2.4% vs 1.8%) (figure 3; online supplemental figure S3). CFRs were sixfold greater for MI than IHD overall (12.0% vs 2.0%; figure 3), with higher rates for MI in rural than urban areas (12.6% vs 11.8%), women than men (14.6% vs 11.5%) (figure 3; online supplemental figure S3) and tier 1 than tier 2 and 3 hospitals (14.1% vs 10.4% and 9.6%; figure 3; online supplemental figure S4).

Additional details of cumulative event rates occurring after 28 days following first MI and all IHD events are shown separately by sex (online supplemental figure S5), area (online supplemental figure S6), hospital tier (online supplemental figure S7), validation status (online supplemental figure S8), different event types (online supplemental figure S9) and subsequently for events occurring before and after 28 days for reported events (online supplemental figure S10) and adjudicated events (online supplemental figure S11). Among survivors, cumulative recurrence rates for MI from 28 days after first IHD of any type were 2%, 6% and 11%, at 1 year, 5 years and 10 years, and after first MI were 6%, 14% and 24%, respectively (online supplemental table S20). The 5-year all-cause mortality rates following first hospitalisation were 21% for MI and 15% for all IHD, respectively, while rates of hospitalisation with HF showed little variation (4% for both MI and all IHD; figure 2; online supplemental table S20).

Cumulative stroke rates after first IHD were 5%, 20% and 34%, at 1 year, 5 years and 10 years, with lower rates after first MI of 4%, 16% and 27%, respectively (figure 2; online supplemental table S20). Overall, cumulative all-cause mortality rates after first adjudicated MI were 10% at 28 days, 17% at 1 year, 31% at 5 years and 46% at 10 years (online supplemental figure S11; online supplemental table S21) with similar patterns after first reported cases (online supplemental figures S9 and S10; online supplemental table S22).

### Prognosis of MI and all IHD cases by subgroups

Figure 3 shows the mortality rates (95% CI) at 28 days and 5 years for MI and all IHD, by age, sex, area and hospital tier. The 5-year mortality rates following MI and all IHD cases increased exponentially with age and were greater in men than women (online supplemental figure S5), rural than urban areas (online supplemental figure S6) and in lower tier than higher tier hospitals (online supplemental figure S7). The differences in 28-day mortality rates among MI cases were less extreme than for all IHD cases in these population subgroups.

At 5 years, MI recurrence and all-cause mortality rates were higher in men than women for all IHD, whereas, among MI cases, women had higher recurrence rates (15.5% vs 11.8%) and all-cause mortality (21.6% vs 20.3%) (online supplemental figure S5). The 5-year mortality rates were also higher in rural than urban areas for MI and all IHD cases, while the 5-year stroke rates after all IHD cases were higher in cases in urban than rural areas (22.0% vs 17.5%) (online supplemental figure S6). For incident MI, recurrence and mortality rates were lowest in tier 3 hospitals (11.9% and 18.6%) than tier 2 hospitals (14.1% and 24.3%) (online supplemental figure S7). By contrast, outcomes were comparable across validation categories (online supplemental figure S8).

Following the first adjudicated MI, 5-year ischaemic stroke rates were similar to those for recurrent MI (14.7% vs 13.1%; online supplemental figure S12; figure 2). The 5-year cumulative stroke rates (95% CI) following IHD were higher for all IHD than for MI (20.5% (19.8% to 21.2%) vs 16.0% (14.0% to 18.0%); figure 2), chiefly reflecting ischaemic stroke subtypes (online supplemental figure S12), but the absolute stroke numbers were small in the MI group. In contrast, 5-year cumulative IHD rates after first MI exceeded those after all IHD (42.0% vs 33.0%), while rates of other CVD outcomes were lower (24.0% vs 33.5% for MI vs all IHD; online supplemental figure S13).

## Discussion

This report on the diagnostic accuracy, use of treatment and prognosis of reported first incident MI and all IHD cases in a community-based prospective study in China identified 1948 MI cases, with a diagnostic accuracy of nearly 98% for MI and 93% for all IHD. While the use of any guideline-directed medications in hospital was high (93% MI, 83% all IHD cases), use of coronary artery revascularisation was low (39% MI, 10% all IHD). Among first incident hospitalised MI cases, 12% died within 28 days and 20% died within 5 years, but 13% experienced recurrent MI and 16% had a stroke within 5 years, and 10% of survivors were hospitalised for HF within 10 years. The short-term and long-term outcomes varied by subgroups, with worse mortality outcomes in older people, women, residents in rural areas and cases treated in lower tier hospitals, highlighting the need to prioritise improvements in treatment in these population subgroups.

The diagnostic accuracy of reported MI cases in China was comparable with hospitalised IHD subtypes in Western populations.^26^ Previous population-based studies involving linkage to hospital episode statistics in the UK or Australia reported that MI cases comprised ~30% of all hospitalised IHD cases, which were higher than in China.^27 28^ While increasing use of cardiac biomarkers during follow-up in CKB might be expected to accompany a higher incidence of MI^3^ and higher lipid levels in later calendar periods in China,^29^ the China Patient-centred Evaluative Assessment of Cardiac Events (PEACE)–Retrospective AMI Study reported no such associations.^30^

A retrospective study of MI cases in six high-income countries reported revascularisation rates of 37–79% (2011–2017),^31^ comparable with the present study (39%; 45% in tier 3 hospitals). Between 2013 and 2014, the nationwide China Acute Myocardial Infarction (CAMI) Registry reported that 43% of ST-segment elevation MI (STEMI) cases in tier 3 hospitals had coronary revascularisation,^32^ while the Beijing Acute Myocardial Infarction Study (2007–2012, 60 000 MI cases) found revascularisation rates of 33–51%, consistent with CKB.^33^ Similarly, the rates of antiplatelet, lipid-lowering and antihypertensive medication use (87%, 79% and 73%, respectively) in CKB were broadly consistent with previous reports. The nationwide Improving Care for Cardiovascular Disease in China–Acute Coronary Syndrome registry (57 560 MI cases from tier 3 hospitals, 2014–2019) reported the highest rates for dual antiplatelet therapy (96%) and statins (93%) and the lowest for beta-blockers (68%) and ACE inhibitors/angiotensin receptor blockers (55%).^34^

Consistent with previous reports, revascularisation rates among MI cases were higher in men than women and in urban than rural areas.^35 36^ Differences may reflect improved access to specialist care and higher levels of health literacy in urban areas and under-diagnosis in women with atypical symptoms.^37^ Coronary revascularisation was more frequent in younger patients and higher-tier hospitals, reflecting higher procedural risks in older patients and greater access to facilities. While women were less likely than men to undergo revascularisation, a previous CKB report suggested that anatomical rather than socioeconomic factors accounted for the lower use of coronary artery revascularisation in women than men.^38^ Although use of revascularisation in CKB remained stable during follow-up, even in tier 3 hospitals, the China PEACE Study reported an increasing use of coronary artery revascularisation and guideline-directed medications during follow-up.^39^

The 30-day CFRs for MI also varied widely between the Organisation for Economic Co-operation and Development countries.^40^ In 2021, Iceland, Norway and the Netherlands reported the lowest rates (1.7–2.9%), while Mexico and Latvia reported the highest CFRs (23.7% and 15.9%).^40^ By contrast, a study of 77 000 MI cases in tier 1 and tier 2 hospitals in China reported an age-standardised 28-day CFR of 18.6% in 2019,^41^ and CKB reported a 28-day CFR of 12.0%. A more recent 2019–2020 nationwide study of 253 chest pain centres in China reported a 30-day CFR of 5.9% among 37 000 MI cases.^42^ A more recent study of >15 000 MI cases from Eastern and Central China reported improved in-hospital survival following implementation of a national accreditation programme to standardise acute IHD care in 2016.^43^

A Swedish registry of 100 000 MI cases (2006–2011) demonstrated a 1-year risk for a composite outcome of non-fatal MI, non-fatal stroke or CVD mortality of 18.3%,^44^ while a study from Sweden, USA, England and France reported 3-year CVD mortality rates of 26–36% and all-cause mortality rates of 20–30%.^45^ In CKB, combining outcomes pre and post 28 days, 1-year MI recurrence and all-cause mortality rates were 14% and 17%, respectively. By contrast, the China PEACE Study reported lower recurrence and all-cause mortality rates of 2.5% and 2.8%, respectively,^46^ but most cases in the latter study were recruited from tier 3 hospitals. A 2013–2014 CAMI Registry study of 23 887 MI cases, involving 30% of cases in tier 3 hospitals, reported a post-30-day to 2-year mortality rate of 6.0%.^47^

The age-specific 28-day CFRs in CKB were higher in women than men (15% vs 12%), consistent with a randomised trial of 40 000 STEMI cases in China^48^ and the China PEACE Study, and these differences may reflect both biological and health system factors, such as delayed diagnosis or under-treatment in women than men.^37^ Several European and US studies also reported higher CFRs in women than men in all age groups despite overall declines.^49^,^54^ A systematic review of 39 studies with ≥5 years of follow-up reported more adverse outcomes in women than men, even after age adjustment.^55^ In the CKB study, 28-day mortality and 5-year recurrence rates after MI were comparable in rural and urban areas (12.6% vs 11.8%, and 15.7% and 13.8%, respectively), but mortality rates were higher in rural than urban areas (25.8% and 18.0%), possibly reflecting poorer socioeconomic status and limitations in access to treatment. Cases in tier 1 hospitals had higher 5-year recurrence and mortality rates (19.5% and 29.4%) than those in tier 3 hospitals (11.9% and 18.6%), suggesting limited access to diagnostic tests and poor adherence to guideline-directed medications and coronary artery revascularisation. Similar disparities were evident in the CAMI registry^11^ and a Sichuan-based cohort of ~70 000 MI patients.^56^ A meta-analysis involving 40 million cases from 37 studies worldwide reported higher mortality rates following MI in rural than urban areas (15.5% vs 13.4%), with the largest disparities in North America and Asia.^57^

European prospective studies reported improved long-term survival and CVD outcomes after MI.^58 59^ In a cohort of >430 000 MI cases in England, one-third developed HF and 38% died during 9 years of follow-up.^60^ In China, evidence on prognosis post MI was restricted to single hospitals,^61^ male occupational cohorts^62^ or single municipal areas.^63^ With 11 years of follow-up, this study reported MI recurrence rates of 13% at 5 years and 23% at 10 years, respectively, and mortality rates of 21% at 5 years and 38% at 10 years, respectively. Hospitalisation rates at 5 years and 10 years after MI were 16% and 27% for stroke, and 4% and 9% for HF.

The chief strengths of this study included its prospective design, inclusion of cases from all hospital tiers, detailed data on baseline characteristics, clinical phenotypes and use of guideline-directed medications and coronary revascularisation and long-term follow-up for fatal and non-fatal CVD outcomes. However, the study also had several limitations, including lack of representativeness for the overall Chinese population and absence of data on use of medication following discharge from hospital, which may be problematic in China.^64^ Additionally, analyses were restricted to 2004–2017, largely preceding the 2016 chest pain centre accreditation programme,^43^ preventing assessment of its impact. Revascularisation rates in CKB may also have been underestimated, as cases from lower-tier or some rural hospitals may have been transferred to tier 3 hospitals outside study areas and, hence, were not recorded.

Despite advances in IHD treatment in China, substantial socioeconomic disparities, reflecting rural-urban differences, persisted during 2004–2017. Poor long-term outcomes following MI – especially in rural populations and lower-tier hospitals—reflect inequalities in access to guideline-directed medications and other treatment including coronary artery revascularisation. While short-term prognosis was more closely associated with hospital tier than geographic area, IHD cases in rural areas experienced worse long-term outcomes, highlighting gaps in care delivery, secondary prevention and referral systems. The study reinforces the need for improvements in public health strategies to prioritise wider use of coronary artery revascularisation following MI, and greater use of guideline-directed medications, both within hospitals and following discharge from hospital, particularly in subgroups identified with poor outcomes.

## Supplementary material

10.1136/bmjph-2025-004019 online supplemental file 1

## Data Availability

Data are available upon reasonable request.
