# Supplementary material for: Diagnostic accuracy, treatment and prognosis of myocardial infarction: an 11-year follow-up of a community-based cohort of 0.5 million Chinese adults
Source: BMJ Public Health. 2026 Mar 10;4(1):e004019. doi: 10.1136/bmjph-2025-004019 (PMC12983722; doi:10.1136/bmjph-2025-004019)
Supplement: online supplemental file 1 [file bmjph-4-1-s001.docx]

**Diagnostic accuracy, treatment and prognosis of myocardial infarction: an 11-year follow-up of a community-based cohort of 0.5 million Chinese adults**

**Supplementary Materials**

[Members of the China Kadoorie Biobank collaborative group: 3](#_Toc207708181)

[Supplementary Methods 4](#_Toc207708182)

[Supplementary References 7](#_Toc207708183)

[Table S1: Study endpoints and associated ICD-10 codes 8](#_Toc207708184)

[Table S2: Baseline characteristics of participants with reported incident IHD with accessible records by subtype 9](#_Toc207708185)

[Table S3: Baseline characteristics of IHD cases by retrieval status of medical records 10](#_Toc207708186)

[Table S4: Baseline characteristics of reported, verified, and adjudication-confirmed IHD cases 11](#_Toc207708187)

[Table S5: Baseline characteristics of fatal reported incident IHD events by hospitalisation status 12](#_Toc207708188)

[Table S6: Reporting and diagnostic accuracy of all reported IHD cases by selected characteristics 13](#_Toc207708189)

[Table S7: Reporting and diagnostic accuracy of hospitalised MI cases by year of diagnosis 14](#_Toc207708190)

[Table S8: Accuracy of reported and verified hospitalised IHD events by subtype at (a) verification and (b) adjudication 15](#_Toc207708191)

[Table S9: Reporting and diagnostic accuracy of hospitalised IHD cases by area 16](#_Toc207708192)

[Table S10: Hospital discharge diagnoses among unverified IHD cases by reported IHD subtype 17](#_Toc207708193)

[Table S11: Non-circulatory disease diagnoses among unverified IHD cases 18](#_Toc207708194)

[Table S12: Physician-adjudicated diagnoses among refuted IHD cases by discharge diagnosis 19](#_Toc207708195)

[Table S13: Non-circulatory disease diagnoses among refuted IHD cases 20](#_Toc207708196)

[Table S14: Reporting and diagnostic accuracy of all IHD cases reported by health insurance and disease registries including participants with prior IHD at baseline 21](#_Toc207708197)

[Table S15: Performance of medical tests 22](#_Toc207708198)

[Table S16: Use of standard medication, revascularisation and traditional Chinese medicine by IHD subtypes and sex 23](#_Toc207708199)

[Table S17: Use of standard medication, revascularisation and traditional Chinese medicine by IHD subtypes and area 24](#_Toc207708200)

[Table S18: Use of standard medication, revascularisation and traditional Chinese medicine by IHD subtypes and hospital tier 25](#_Toc207708201)

[Table S19: Use of standard medication and revascularisation by IHD subtypes by reporting year and age at diagnosis 26](#_Toc207708202)

[Table S20: Cumulative event rates of recurrent MI events, stroke, heart failure and all-cause mortality from 28 days following first adjudicated event of different IHD subtypes 27](#_Toc207708203)

[(a) after first MI 27](#_Toc207708204)

[(b) after first non-MI 28](#_Toc207708205)

[(c) after first IHD 29](#_Toc207708206)

[Table S21: Cumulative event rate of recurrent MI events, stroke, heart failure and all-cause mortality following first adjudicated event of different IHD subtypes (combining before and after 28 days) 30](#_Toc207708207)

[(a) after first MI 30](#_Toc207708208)

[(b) after first non-MI 31](#_Toc207708209)

[(c) after first IHD 32](#_Toc207708210)

[Table S22: Cumulative event rates of recurrent MI events, stroke, heart failure and all-cause mortality from 28 days following first reported event of different IHD subtypes 33](#_Toc207708211)

[(a) after first MI 33](#_Toc207708212)

[(b) after first non-MI 34](#_Toc207708213)

[(c) after first IHD 35](#_Toc207708214)

[Figure S1: Associations of SBP and BMI with risk of MI by adjudication status 36](#_Toc207708215)

[Figure S2: Associations of SBP and BMI with risk of non-MI by adjudication status 37](#_Toc207708216)

[Figure S3: Age-specific 28-day case-fatality rates after first adjudicated event of different IHD types by sex and area 38](#_Toc207708217)

[Figure S4: Age-specific 28-day case-fatality rates after first adjudicated event of different IHD types by hospital tier 39](#_Toc207708218)

[Figure S5: Estimated cumulative event rate of recurrent MI events, stroke, heart failure and all-cause mortality from 28 days after first adjudicated event of different IHD types by sex 40](#_Toc207708219)

[Figure S6: Estimated cumulative event rate of recurrent MI events, stroke, heart failure and all-cause mortality from 28 days after first adjudicated event of different IHD types by area 41](#_Toc207708220)

[Figure S7: Estimated cumulative event rate of recurrent MI events, stroke, heart failure and all-cause mortality following first adjudicated event of different IHD types by hospital tier 42](#_Toc207708221)

[Figure S8: Estimated cumulative event rate of recurrent MI events, stroke, heart failure and all-cause mortality following first event of different IHD types by validation status 43](#_Toc207708222)

[Figure S9: Estimated cumulative event rate of recurrent MI events, stroke, heart failure and all-cause mortality from 28 days after first event of different IHD types 44](#_Toc207708223)

[Figure S10: Estimated cumulative event rate of recurrent MI events, stroke, heart failure and all-cause mortality following first event of different IHD types (combining before and after 28 days) 45](#_Toc207708224)

[Figure S11: Estimated cumulative event rate of recurrent MI events, stroke, heart failure and all-cause mortality following first adjudicated event of different IHD types (combining before and after 28 days) 46](#_Toc207708225)

[Figure S12: Estimated cumulative event rate of stroke types from 28 days after first adjudicated event of different IHD types 47](#_Toc207708226)

[Figure S13: Estimated cumulative event rate of ischaemic heart disease and other cardiovascular disease from 28 days after first adjudicated event of different IHD types 48](#_Toc207708227)

# Members of the China Kadoorie Biobank collaborative group:

**International Steering Committee:** Junshi Chen, Zhengming Chen (PI), Robert Clarke, Rory Collins, Liming Li (PI), Jun Lv, Richard Peto, Robin Walters.

**International Co-ordinating Centre, Oxford:** Daniel Avery, Maxim Barnard, Derrick Bennett, Ka Hung Chan, Yiping Chen, Zhengming Chen, Johnathan Clarke; Robert Clarke, Huaidong Du, Ahmed Edris Mohamed, Hannah Fry, Prapthi Harish, Pek Kei Im, Andri Iona, Christiana Kartsonaki, Hubert Lam, Kuang Lin, James Liu, Iona Millwood, Sam Morris, Qunhua Nie, Alfred Pozarickij, Maryam Rahmati, Paul Ryder, Dan Schmidt, Becky Stevens, Iain Turnbull, Robin Walters, Baihan Wang, Lin Wang, Neil Wright, Ling Yang, Xiaoming Yang, Pang Yao.

**National Co-ordinating Centre, Beijing:** Xiao Han, Can Hou, Qingmei Xia, Chao Liu, Jun Lv, Pei Pei, Dianjianyi Sun, Canqing Yu, Lang Pan.

**10 Regional Co-ordinating Centres:**

**Qingdao CDC:** Zengchang Pang, Ruqin Gao, Shanpeng Li, Haiping Duan, Shaojie Wang, Yongmei Liu, Ranran Du, Yajing Zang, Liang Cheng, Xiaocao Tian, Hua Zhang, Yaoming Zhai, Feng Ning, Xiaohui Sun, Feifei Li. **Licang CDC:** Silu Lv, Junzheng Wang, Wei Hou. **Heilongjiang Provincial CDC:** Wei Sun, Shichun Yan, Xiaoming Cui. **Nangang CDC:** Chi Wang, Zhenyuan Wu, Yanjie Li, Quan Kang. **Hainan Provincial CDC:** Huiming Luo, Tingting Ou. **Meilan CDC:** Xiangyang Zheng, Zhendong Guo, Shukuan Wu, Yilei Li, Huimei Li. **Jiangsu Provincial CDC:** Ming Wu, Yonglin Zhou, Jinyi Zhou, Ran Tao, Jie Yang, Jian Su. **Suzhou CDC:** Fang Liu, Jun Zhang, Yihe Hu, Yan Lu, Liangcai Ma, Aiyu Tang, Shuo Zhang, Jianrong Jin, Jingchao Liu. **Guangxi Provincial CDC:** Mei Lin, Zhenzhen Lu. **Liuzhou CDC:** Lifang Zhou, Changping Xie, Jian Lan, Tingping Zhu, Yun Liu, Liuping Wei, Liyuan Zhou, Ningyu Chen, Yulu Qin, Sisi Wang. **Sichuan Provincial CDC:** Xianping Wu, Ningmei Zhang, Xiaofang Chen, Xiaoyu Chang. **Pengzhou CDC:** Mingqiang Yuan, Xia Wu, Xiaofang Chen, Wei Jiang, Jiaqiu Liu, Qiang Sun. **Gansu Provincial CDC:** Faqing Chen, Xiaolan Ren, Caixia Dong. **Maiji CDC:** Hui Zhang, Enke Mao, Xiaoping Wang, Tao Wang, Xi zhang. **Henan Provincial CDC:** Kai Kang, Shixian Feng, Huizi Tian, Lei Fan. **Huixian CDC:** XiaoLin Li, Huarong Sun, Pan He, Xukui Zhang. **Zhejiang Provincial CDC:** Min Yu, Ruying Hu, Hao Wang. **Tongxiang CDC**: Xiaoyi Zhang, Yuan Cao, Kaixu Xie, Lingli Chen, Dun Shen. **Hunan Provincial CDC:** Xiaojun Li, Donghui Jin, Li Yin, Huilin Liu, Zhongxi Fu. **Liuyang CDC:** Xin Xu, Hao Zhang, Jianwei Chen, Yuan Peng, Libo Zhang, Chan Qu.

**Event Adjudication Clinicians:**

**Beijing Tiantan Hospital, Capital Medical University** Shuya Li, Haiqiang Qin, Yongjun Wang, **Peking University People's Hospital** Qiling Chen, Jihua Wang, **The 1^st^** **Affiliated Hospital of Harbin Medical University** Xiaojia Sun, Lei Wang, Xun Wang, Liming Zhang, Shanshan Zhou, **The 2^nd^ Affiliated Hospital of Harbin Medical University** Hongyuan Chen, Li Chen, Haiyan Gou, Weizhi Wang, Yanmei Zhu, Yulan Zhu, **The 2^nd^ Hospital of Hebei Medical University** Ning Zhang, **Huashan Hospital** Xin Cheng, Qiang Dong, Yi Dong, Kun Fang, Yiting Mao, **Jinling Hospital** Yu An, Peiling Chen, Yinghua Chen, Zhihong Liu, Lihua Zhang, **The People's Hospital of Liaoning Province** Xiaohong Chen, Naixin Jv, Xiaojiu Li, Liyang Liu, Yun Lu, Xiaona Xing, **Qingdao Fuwai Cardiovascular Hospital** Shihao You, **Shengjing Hospital of China Medical University** Xiaoli Cheng, Chaojun Gua, Jinping Jiang, Jingyi Liu, Shumei Ma, **Shenyang Military General Hospital** Xuefeng Yang, **The First People's Hospital of Shenyang** Xiaomo Du, Jian Xu, Xuecheng Yang, Xiaodi Zhao, **West China Hospital, Sichuan University** Zilong Hao, Ming Liu, Deren Wang, **The Second Affiliated Hospital of Suzhou University** Xiaoting Li, **Suzhou Kowloon Hospital** Shanghai Jiao Tong, **University School of Medicine** Lili Hui, Zhanling Liao, Feng Liu, **Qingdao Fuwai Cardiovascular Hospital** Chunning Feng, Dejiang Ji, Fengxia Qu, Wenwen Yuan, **The First Affiliated Hospital of Zhengzhou University** Xin Fu, **Zhongshan Hospital** Jing Ding, Peng Du, Lirong Jin, Yueshi Mao, Xin Wang.

# Supplementary Methods

*Retrieval and verification of reported IHD subtypes*

Medical records of all reported first incident cases of ischaemic heart disease (IHD) were sought for verification and adjudication from accessible hospitals (functioning hospitals in the study areas but excluding military hospitals). Hospitals located outside the study regions, military hospitals or hospitals that had recently closed were considered inaccessible. Verification of reported IHD cases with retrieved records was conducted by trained local CKB staff using bespoke software (Portable Validation Device [PVD]) with a high-resolution camera for capturing medical records. Photographs were taken of the initial page, discharge summary (including discharge diagnosis), electrocardiogram (ECG) reports, results of troponin and cardiac enzyme assays, and coronary angiography records in addition to vital status at discharge.^(1)^ Verified diagnoses of IHD without sufficient accompanying clinical information were considered to be secondary, i.e., additional health conditions that were not the main reason for admission, and these cases were not selected for adjudication. If a reported IHD diagnosis was not found in the medical records (“unverified case”), any other non-IHD diagnoses were recorded as free-text and later assigned International Classification of Diseases 10^th^ revision (ICD-10) codes using bespoke disease standardisation software. Subsequently, these diagnoses were classified into cardiovascular disease (CVD)-related (non-IHD cardiac disease, cerebrovascular, and other circulatory diseases), non-CVD-related diseases, or other unclassified diseases (**Figure 1**, **Table S1**).

*Adjudication of IHD subtypes*

All first incident cases with verified primary IHD (i.e., where IHD was the main condition treated during admission), were adjudicated by one of 21 licensed cardiologists from 8 major Tier 3 hospitals who worked as Senior Registrars under the supervision of Consultants Cardiologists. Using a bespoke web-based platform (internet-based Case Adjudication System for clinical Events [*i*-CASE]), adjudicators were invited to review electronic copies of medical records and complete a standardised electronic form, which recorded details on clinical features and diagnostic criteria (including ECG, biomarkers of myocardial injury, and coronary angiography). Myocardial infarction (MI) was defined using World Health Organization (WHO) criteria as presence of any two or more of: (1) anginal chest pain; (2) ECG changes (pathological Q waves, ST-segment elevation or ST-segment depression, T-wave inversion, new left bundle branch block [LBBB]); and (3) elevated plasma levels of cardiac enzymes (CK-MB) or troponins. Use of guideline-directed treatments was also documented in the *i*-CASE form, including medications (antiplatelet, lipid-lowering, antihypertensive, and anticoagulant agents) and any revascularisation therapy, which encompassed any coronary intervention (percutaneous coronary intervention [PCI] or coronary artery bypass graft [CABG]) and thrombolysis. Using these data sources, the cardiologists adjudicated the reported diagnoses of IHD as MI or non-MI IHD subtypes (including non-MI acute IHD and chronic IHD), and any refuted cases of IHD were given a non-IHD diagnosis where possible. Diagnoses for refuted cases were subsequently assigned appropriate ICD-10 codes by study clinicians using bespoke standardisation software and classified as CVD-related (non-IHD cardiac disease, cerebrovascular, and other circulatory diseases), non-CVD diseases, or other unclassified diseases.

***IHD validation status***

- ***Reported cases***
  - First incident IHD cases identified from routine data sources, namely electronic linkage (via unique national identifiers) with death and disease registers and the nationwide Health Insurance (HI) system, supplemented by active follow-up to capture events not recorded from these sources (including for participants who had moved out of study areas)
- ***Verified cases***
  - Reported cases for which hospital medical records containing documentary evidence of IHD diagnosis were successfully retrieved by local CKB staff
  - Verification required photographic capture of discharge summaries (with discharge diagnosis) with or without ECG, troponin or cardiac enzyme results, angiography reports, and vital status at discharge
  - A case was classified as ***unverified*** if retrieved records did not contain an IHD diagnosis
- ***Adjudication-confirmed cases***
  - Verified IHD cases independently reviewed by trained cardiologists to confirm diagnoses of MI or non-MI IHD subtypes
  - Adjudicators applied standardised diagnostic criteria (ECG, biomarkers of myocardial injury, angiography, clinical features, and treatments)
  - A case was classified as ***refuted*** by adjudicators if the provided clinical information did not support the verified diagnosis of MI or non-MI IHD subtypes

*Quality control*

Quality control procedures involved assessing both the accuracy and consistency of diagnoses. ^(1)^ Consistency was evaluated by comparing adjudicated diagnoses for the same clinical event between two different adjudicators, either centrally or within routine processes using random subsets of cases (e.g., 10% of disease events). The alignment with international standard criteria was also checked by comparing adjudicated diagnoses to computer-generated diagnoses based on standard algorithms. Any substantial discrepancies between adjudicators or deviations from standard criteria were flagged for further investigation and additional central review by study clinicians with relevant training in cardiology.

*Statistical analysis*

Retrieval rates were defined as the proportions of cases with hospital records retrieved among the total number of reported accessible IHD cases (excluding reported deaths). Reporting accuracy (i.e., the proportion of retrieved reported cases with evidence of IHD found in hospital records during disease verification) and diagnostic accuracy of IHD (i.e., the proportion of all verified primary IHD cases that were confirmed during independent adjudication) were estimated using positive predictive values (PPV), for any IHD and MI. Only participants with incident diagnoses of IHD reported by health insurance (HI) or disease register were included in analyses of accuracy. All analyses were conducted using R (version 4.2.2).

*Additional details on verification of diagnoses of IHD subtypes*

Successful retrieval rates of medical records were higher in cases reported by HI than by disease registers (83.4% vs 75.4%), in those reported in later than in earlier years of follow-up (90.5% in 2015 vs 75.8% in 2004 – 2008), and in those reported from Tier 3 than Tier 2 or Tier 1 hospitals (91.5% vs 83.0% vs 72.4%; **Table S6**). In addition, retrieval was more successful in urban than rural areas (92.6% vs 70.8%; **Table S6**), with highest rates in Liuzhou (urban; 94.7%) and lowest rates in Gansu (rural; 53.6%) (**Table S9**). Reported IHD cases differed by retrieval status, with a higher proportion of MI cases where records were unretrieved than retrieved (10% vs 6%). Unretrieved cases were more frequent in rural than in urban areas (79% vs 43%) and in younger than older people at baseline (56.9 vs 58.9 years) (**Table S3**). While 49% of participants with reported IHD cases lived in rural areas, with 42% for verified cases and 37% for adjudication-confirmed cases, the baseline characteristics of these groups were otherwise similar (**Table S4**).

Overall, the PPVs were higher for diagnoses reported in urban than in rural areas (87.3% vs 83.2%; **Table S6**), with highest reporting accuracy in Qingdao (urban; 95.7%) and lowest in Zhejiang (rural; 69.7%), respectively (**Table S9**). The PPV for cases reported by HI were higher than those reported by disease registers (85.9% vs 84.0%), but rates were comparable by sex. The PPV for reported IHD cases increased by calendar years of follow-up (80.6% for 2008 vs 89.5% for 2015; **Table S6**). Among retrieved IHD cases verified from available records, 2,025 (7%) were MI, of which 962 (48%) were originally reported as MI while 1,063 (52%) were reclassified from non-MI IHD cases (**Table S8**).

Among 4,397 (14%) cases without a verified diagnosis of IHD in retrieved medical records, 202 (5%) had a reported diagnosis of MI IHD (**Table S10**; **Figure 1**). Among all unverified IHD cases, 936 (21%) had non-IHD cardiac diseases, of which 37 (4%) had heart failure and 113 (12%) had valvular heart disease. A total of 1,611 (37%) had cerebrovascular disease and 1,720 (39%) had non-circulatory disease, among which 163 (9%) cases had chronic obstructive pulmonary disease (ICD-10 J44) and 72 (4%) cases had gastritis and duodenitis (ICD-10 K29) (**Table S11**).

*Additional details on adjudication of IHD subtypes*

The PPV for adjudicated IHD was higher for cases from urban than from rural areas (94.3% vs 91.0%; **Table S6**), with highest and lowest diagnostic accuracy in Liuzhou (urban; 94.7%) and Zhejiang (rural; 84.6%), respectively (**Table S9**). Diagnostic accuracy was higher in those reported by HI than by disease registers (93.5% vs 91.1%), but was similar in Tier 3 and Tier 2 hospitals (93.4% vs 92.7%), and in men and women (93.6% vs 92.7%) (**Table S6**). In addition, PPV increased by year of reporting for adjudication-confirmed IHD (90.6% in 2008 vs 93.5% in 2015; **Table S6**) and MI (95.3% for 2008 vs 99.5% for 2015; **Table S7**).

Among all adjudication-confirmed diagnoses of IHD, 1,948 (10.2%) had MI, 81% (n=1,588) of which were initially verified as MI (**Table S8**; **Figure 1**). Of 1,426 (7%) refuted primary IHD cases, 25 (2%) had a discharge diagnosis of MI (**Table S12**; **Figure 1**). Overall, 448 (31%) had non-IHD cardiac diseases, of which 143 (32%) had atrial fibrillation and 149 (33%) had other arrhythmias (**Table S12**). A total of 209 (15%) refuted cases had a diagnosis of cerebrovascular disease and 721 (51%) had non-circulatory disease, among which most (568; 79%) had no codable diagnosis, 34 (5%) had abnormal findings on diagnostic imaging of central nervous system (ICD-10 R90), and 20 (3%) had other respiratory disorders (ICD-10 J98) (**Table S13**). In participants with self-reported IHD at baseline, the PPV for adjudicated IHD was slightly higher than in those with first incident IHD cases (94.2% vs 93.1%; p= 0.0057; **Table S14**).

# Supplementary References

1. Chen Y, Clarke R. Verification and Adjudication of Health Outcomes in Prospective Cohort Studies, in Population Biobank Studies: A Practical Guide. 2020: p. 123-143.

# Table S1: Study endpoints and associated ICD-10 codes

|  | | **Number of cases** | |
| --- | --- | --- | --- |
| **Disease types** | **ICD-10 codes** | **At verification N (%)** | **At adjudication N (%)** |
| **Ischaemic heart disease (IHD)** | **I20 - I25** | **26,002 (86)** | **19,135 (93)** |
| **Myocardial infarction (MI)** | **I21 - I23, I25.2** | **2,422 (8)** | **2,436 (12)** |
| Acute MI | I21 | 2,025 (7) | 1,911 (9) |
| Subsequent MI | I22 |  | 37 (0) |
| Complications following MI | I23 |  |  |
| STEMI (ST elevation myocardial infarction) | I21.0 - I21.3, I22.0 - 122.8, I23 |  | 830 (4) |
| NSTEMI (Non-ST elevation myocardial infarction) | I21.4 |  | 468 (2) |
| Unspecified MI | I21.9, I22.9 |  | 650 (3) |
| Old MI | I25.2 | 397 (1) | 488 (2) |
| **Non-MI** | **I20, I24, I25, excluding I25.2** | **23,580 (78)** | **16,699 (81)** |
| **Non-MI acute IHD** | **I20, I24** | **8,714 (29)** | **10,748 (52)** |
| Angina | I20 | 8,389 (28) | 10,748 (52) |
| Other acute IHD | I24 | 325 (1) |  |
| **Chronic IHD** | **I25.0, I25.1, I25.3 - I25.9** | **14,866 (49)** | **5,951 (29)** |
| Atherosclerotic heart disease | I25.0, I25.1 | 235 (1) | 7 (0) |
| Aneurysm of heart | I25.3 | 1 (0) |  |
| Coronary aneurysm and dissection | I25.4 |  |  |
| Ischaemic cardiomyopathy | I25.5 | 43 (0) |  |
| Silent myocardial ischaemia | I25.6 | 3 (0) | 7 (0) |
| Other chronic IHD | I25.8 |  |  |
| Unspecified chronic IHD | I25.9 | 52 (0) | 5,936 (29) |
| **Non-IHD cardiac disease** | **I00 - I09, I26 - I52** | **936 (3)** | **448 (2)** |
| Heart failure | I50 | 37 (0) | 74 (0) |
| Atrial fibrillation | I48 | 27 (0) | 143 (1) |
| Other arrhythmia | I47, I49 | 221 (1) | 149 (1) |
| Cardiomyopathy | I42 - I43 | 61 (0) | 4 (0) |
| Valvular heart disease | I00 - 109, I34 - I39 | 113 (0) | 15 (0) |
| Pulmonary heart disease and diseases of pulmonary circulation | I26 - I28 | 46 (0) | 9 (0) |
| Cardiac arrest | I46 | 1 (0) |  |
| Other non-IHD cardiac disease | I30 - I33, I40 - I41, I44 - 145, I51 - I52 | 430 (1) | 54 (0) |
| **Cerebrovascular disease** | **I60 - I69** | **1,611 (5)** | **209 (1)** |
| **Hypertensive disease** | **I10 - I15** | **93 (0)** | **45 (0)** |
| **Other circulatory disease** | **I70 - I79, I80 - I89, I95 - I99** | **37 (0)** | **3 (0)** |
| **Non-circulatory disease** | **All codes, excluding I00 - I99** | **1,720 (6)** | **721 (4)** |
| ICD-10 = International Classification of Diseases 10th revision | | | |

# Table S2: Baseline characteristics of participants with reported incident IHD with accessible records by subtype

|  | **MI** | **Non-MI** | **Any IHD** | **All participants** |
| --- | --- | --- | --- | --- |
| **Mean (SD) or n (%)** | (N = 2,620) | (N = 34,539) | (N = 37,159) | (N = 497,254) |
| **Demographic factors** |  |  |  |  |
| Age at baseline, years | 60.3 (9.8) | 58.9 (9.9) | 59.0 (9.9) | 51.7 (10.6) |
| Age at first onset, years | 66.2 (10.0) | 65.1 (10.1) | 65.1 (10.1) | 62.2 (10.2) |
| Male | 1,587 (65) | 13,305 (39) | 14,892 (41) | 204,487 (41) |
| Rural resident | 1,359 (57) | 17,028 (51) | 18,387 (51) | 281,570 (57) |
| High school or higher education | 530 (19) | 8,029 (21) | 8,559 (21) | 103,175 (21) |
| Household income ≥20,000 yuan/year | 995 (42) | 13,557 (43) | 14,552 (43) | 212,155 (43) |
| **Lifestyle factors** |  |  |  |  |
| Current regular alcohol drinker |  |  |  |  |
| Men | 602 (39) | 5,794 (44) | 6,396 (43) | 85,728 (42) |
| Women | 32 (5) | 858 (3) | 890 (3) | 8,621 (3) |
| Current regular smoker |  |  |  |  |
| Men | 1,016 (72) | 7,560 (64) | 8,576 (65) | 125,955 (62) |
| Women | 66 (5) | 931 (3) | 997 (3) | 6,762 (2) |
| Total physical activity, MET-h/day | 19.4 (12.4) | 20.3 (11.6) | 20.4 (11.6) | 21.3 (13.9) |
| **Medical history^*^** |  |  |  |  |
| Prior stroke/TIA | 126 (3) | 1,239 (2) | 1,365 (2) | 7,657 (2) |
| Prior and screen-detected diabetes | 406 (14) | 3,821 (8) | 4,227 (9) | 27,602 (6) |
| **Medication use^†^** |  |  |  |  |
| Statin | 7 (0.2) | 127 (0.4) | 134 (0.3) | 930 (0.2) |
| Any antihypertensive agent | 546 (15) | 6,123 (15) | 6,669 (15) | 42,107 (8) |
| Aspirin | 68 (2) | 644 (1) | 712 (1) | 3,823 (1) |
| **Anthropometry** |  |  |  |  |
| BMI, kg/m² | 24.1 (3.5) | 24.3 (3.7) | 24.3 (3.7) | 23.6 (3.4) |
| SBP, mmHg | 138.1 (24.1) | 134.9 (23.2) | 135.0 (23.3) | 130.8 (21.2) |
| RHR, bpm | 79.7 (12.2) | 79.3 (12.1) | 79.3 (12.1) | 78.9 (11.8) |
| **Length of hospital stay, days^‡^** | 9 (6-13) | 8 (5-12) | 8 (5-12) | – |
| Excluding participants with prior IHD. | | | | |
| Means and percentages are directly standardised to the age (at baseline, 5 year intervals), sex, and study region structure of the CKB cohort after excluding participants with prior IHD, as appropriate. | | | | |
| Non-MI includes non-MI acute IHD and chronic IHD cases. | | | | |
| ^*^ Self-reported at baseline questionnaire | | | | |
| ^†^ Self-reported taking blood pressure medication and/or taking ACE inhibitors, beta-blockers, diuretics, or calcium channel blockers. | | | | |
| ^‡^ Median (IQR) | | | | |
| MI = myocardial infarction; IHD = ischaemic heart disease; TIA = transient ischaemic attack; BMI = body mass index; SBP = systolic blood pressure; RHR = heart rate; SD = standard deviation. | | | | |

# Table S3: Baseline characteristics of IHD cases by retrieval status of medical records

|  | **Records accessible** | | **Records retrieved** | |
| --- | --- | --- | --- | --- |
|  | **Yes** | **No** | **Yes** | **No** |
| **Mean (SD) or n (%)** | (N = 37,159) | (N = 6,482) | (N = 30,399) | (N = 6,760) |
| **Reported IHD type^**^** |  |  |  |  |
| MI | 2,620 (7) | 392 (6) | 1,939 (6) | 681 (10) |
| Non-MI | 34,539 (93) | 6,090 (94) | 28,460 (94) | 6,079 (90) |
| **Demographic factors** |  |  |  |  |
| Age at baseline, years | 58.4 (9.9) | 56.3 (9.9) | 58.9 (9.8) | 56.9 (10.4) |
| Age at first onset, years | 64.5 (10.1) | 66.0 (10.2) | 65.2 (9.8) | 62.0 (10.7) |
| Male | 14,892 (40) | 2,599 (41) | 12,137 (40) | 2,755 (39) |
| Rural resident | 18,387 (50) | 3,786 (59) | 13,009 (43) | 5,378 (79) |
| High school or higher education | 8,559 (23) | 1,339 (23) | 7,695 (23) | 864 (22) |
| Household income ≥20,000 yuan/year | 14,552 (40) | 2,929 (40) | 12,370 (40) | 2,182 (39) |
| **Lifestyle factors** |  |  |  |  |
| Current regular alcohol drinker |  |  |  |  |
| Men | 6,396 (43) | 1,043 (42) | 5,399 (43) | 997 (43) |
| Women | 890 (4) | 113 (4) | 744 (4) | 146 (4) |
| Current regular smoker |  |  |  |  |
| Men | 8,576 (58) | 1,577 (57) | 6,847 (58) | 1,729 (57) |
| Women | 997 (4) | 99 (4) | 844 (4) | 153 (5) |
| Total physical activity, MET-h/day | 16.2 (11.6) | 16.6 (13.3) | 16.1 (11.0) | 16.5 (13.4) |
| **Medical history^*^** |  |  |  |  |
| Prior stroke/TIA | 1,365 (4) | 133 (2) | 1,218 (4) | 147 (3) |
| Prior and screen-detected diabetes | 4,227 (11) | 505 (9) | 3,722 (11) | 505 (10) |
| **Medication use^†^** |  |  |  |  |
| Statin | 134 (0.4) | 22 (0.3) | 114 (0.4) | 20 (0.2) |
| Any antihypertensive agent | 6,669 (18) | 918 (15) | 5,707 (18) | 962 (16) |
| Aspirin | 712 (2) | 75 (1) | 597 (2) | 115 (2) |
| **Anthropometry** |  |  |  |  |
| BMI, kg/m² | 24.3 (3.7) | 24.2 (3.6) | 24.4 (3.7) | 24.2 (3.6) |
| SBP, mmHg | 138.2 (23.3) | 136.5 (22.7) | 138.6 (23.3) | 137.0 (23.3) |
| RHR, bpm | 78.6 (12.1) | 78.3 (12.1) | 78.6 (12.1) | 78.6 (12.4) |
| **Length of hospital stay, days^‡^** | 8 (5-12) | 7 (2-10) | 9 (6-13) | 6 (3-10) |
| Means and percentages are directly standardised to the age (at baseline, 5 year intervals), sex, and study region structure of IHD cases, as appropriate. | | | | |
| Non-MI includes non-MI acute IHD and chronic IHD cases. | | | | |
| ^*^ Self-reported at baseline questionnaire | | | | |
| ^**^ percentages not standardised | | | | |
| ^†^ Self-reported taking blood pressure medication and/or taking ACE inhibitors, beta-blockers, diuretics, or calcium channel blockers. | | | | |
| ^‡^ Median (IQR) | | | | |
| TIA = transient ischaemic attack; BMI = body mass index; SBP = systolic blood pressure; RHR = heart rate; SD = standard deviation. | | | | |

# Table S4: Baseline characteristics of reported, verified, and adjudication-confirmed IHD cases

|  | **Reported cases*** | **Verified cases** | **Adjudication-confirmed cases** |
| --- | --- | --- | --- |
| **Mean (SD) or n (%)** | (N = 37,159) | (N = 26,002) | (N = 19,135) |
| **Reported IHD type^***^** |  |  |  |
| MI | 2,620 (7) | 1,732 (7) | 1,507 (8) |
| Non-MI | 34,539 (93) | 24,270 (93) | 17,628 (92) |
| **Demographic factors** |  |  |  |
| Age at baseline, years | 58.4 (9.9) | 59.0 (9.7) | 58.8 (9.7) |
| Age at first onset, years | 64.5 (10.1) | 65.6 (9.6) | 65.3 (9.6) |
| Male | 14,892 (40) | 10,332 (40) | 7,760 (41) |
| Rural resident | 18,387 (49) | 10,820 (42) | 7,004 (37) |
| High school or higher education | 8,559 (23) | 6,621 (23) | 5,193 (23) |
| Household income ≥20,000 yuan/year | 14,552 (39) | 10,574 (39) | 8,140 (39) |
| **Lifestyle factors** |  |  |  |
| Current regular alcohol drinker |  |  |  |
| Men | 6,396 (43) | 4,586 (43) | 3,374 (43) |
| Women | 890 (4) | 636 (4) | 376 (4) |
| Current regular smoker |  |  |  |
| Men | 8,576 (58) | 5,851 (58) | 4,443 (59) |
| Women | 997 (4) | 738 (4) | 465 (4) |
| Total physical activity, MET-h/day | 15.8 (11.6) | 15.7 (10.9) | 15.7 (10.9) |
| **Medical history^**^** |  |  |  |
| Prior stroke/TIA | 1,365 (4) | 1,014 (4) | 727 (3) |
| Prior and screen-detected diabetes | 4,227 (11) | 3,255 (12) | 2,435 (12) |
| **Medication use^†^** |  |  |  |
| Statin | 134 (0.4) | 94 (0.4) | 60 (0.4) |
| Any antihypertensive agent | 6,669 (18) | 4,910 (19) | 3,579 (18) |
| Aspirin | 712 (2) | 520 (2) | 400 (2) |
| **Anthropometry** |  |  |  |
| BMI, kg/m² | 24.4 (3.7) | 24.5 (3.6) | 24.5 (3.6) |
| SBP, mmHg | 138.3 (23.3) | 138.8 (23.2) | 138.9 (23.1) |
| RHR, bpm | 78.6 (12.1) | 78.5 (12.0) | 78.4 (11.9) |
| **Length of hospital stay, days^‡^** | 8 (5-12) | 9 (6-13) | 9 (6-13) |
| Excluding participants with prior IHD. | | | |
| Non-MI includes non-MI acute IHD and chronic IHD cases. | | | |
| Means and percentages are directly standardised to the age (at baseline, 5 year intervals), sex, and study region structure of the cases with accessible records, as appropriate.  * Reported IHD cases with accessible records | | | |
| ^**^ Self-reported at baseline questionnaire | | | |
| ^**^ percentages not standardised | | | |
| ^†^ Self-reported taking blood pressure medication and/or taking ACE inhibitors, beta-blockers, diuretics, or calcium channel blockers. | | | |
| ^‡^ Median (IQR) | | | |
| TIA = transient ischaemic attack; BMI = body mass index; SBP = systolic blood pressure; RHR = heart rate; SD = standard deviation. | | | |

# Table S5: Baseline characteristics of fatal reported incident IHD events by hospitalisation status

|  | **Death pre-hospital** | | **Death 0 - 28 days** | | **Death 28 days - 5 years** | |
| --- | --- | --- | --- | --- | --- | --- |
| **Mean (SD) or n (%)** | **MI (n = 2,370)** | **Non-MI (n = 1,675)** | **MI (n = 689)** | **Non-MI (n = 129)** | **MI (n = 793)** | **Non-MI (n = 821)** |
| **Demographic factors** |  |  |  |  |  |  |
| Age at baseline, years | 63.2 (9.9) | 64.9 (8.9) | 62.7 (9.4) | 65.8 (6.9) | 64.0 (8.6) | 65.0 (7.8) |
| Age at death, years | 69.7 (10.3) | 71.5 (9.3) | 69.4 (10.0) | 73.1 (7.4) | 71.6 (9.0) | 72.8 (7.9) |
| Sex, male | 1,347 (58) | 940 (53) | 377 (55) | 61 (48) | 415 (53) | 415 (49) |
| Rural | 1,706 (72) | 775 (46) | 525 (75) | 51 (42) | 552 (69) | 408 (50) |
| High school or higher education | 256 (13) | 252 (11) | 64 (11) | 14 (10) | 76 (10) | 116 (13) |
| Household income ≥ 20,000 yuan/year | 469 (24) | 490 (23) | 141 (26) | 38 (30) | 191 (26) | 266 (29) |
| **Lifestyle factors** |  |  |  |  |  |  |
| Current regular alcohol drinker |  |  |  |  |  |  |
| Men | 433 (38) | 379 (39) | 136 (40) | 19 (33) | 142 (34) | 165 (40) |
| Women | 31 (4) | 33 (5) | 14 (3) | 3 (4) | 10 (3) | 20 (5) |
| Current smoker |  |  |  |  |  |  |
| Men | 827 (57) | 529 (58) | 215 (59) | 34 (57) | 269 (63) | 231 (58) |
| Women | 63 (8) | 53 (6) | 17 (4) | 8 (11) | 23 (7) | 31 (8) |
| Total physical activity, MET-h/day | 12.9 (11.5) | 12.3 (9.6) | 12.7 (11.9) | 13.0 (11.8) | 13.2 (11.5) | 11.4 (9.3) |
| **Medical history and health status^*^** |  |  |  |  |  |  |
| Prior stroke/TIA | 119 (6) | 128 (7) | 48 (9) | 7 (4) | 46 (6) | 65 (8) |
| Prior and screen-detected diabetes | 265 (13) | 291 (15) | 123 (21) | 32 (20) | 148 (19) | 173 (18) |
| **Medication use^†^** |  |  |  |  |  |  |
| Statin | 10 (0.4) | 5 (0.3) | 0 (0.0) | 1 (1.3) | 2 (0.2) | 2 (0.3) |
| Any antihypertensive agent | 435 (21) | 337 (19) | 143 (28) | 32 (25) | 180 (22) | 192 (22) |
| Aspirin | 65 (3) | 39 (3) | 19 (4) | 3 (3) | 22 (3) | 18 (3) |
| **Anthropometry** |  |  |  |  |  |  |
| BMI, kg/m² | 23.3 (3.8) | 23.2 (4.0) | 23.8 (3.7) | 23.4 (4.4) | 23.8 (4.0) | 24.1 (4.1) |
| SBP, mmHg | 145.8 (25.9) | 146.0 (25.5) | 148.3 (24.4) | 147.7 (25.6) | 148.0 (26.1) | 147.6 (24.0) |
| RHR, bpm | 80.5 (12.8) | 79.6 (12.5) | 80.5 (12.6) | 77.3 (12.5) | 80.0 (12.7) | 79.6 (13.6) |
| Means and percentages are directly standardised to the age (at baseline, 5 year intervals), sex, and study region structure of all participants with fatal reported incident IHD, as appropriate. | | | | | | |
| ^*^ Self-reported at baseline questionnaire | | | | | | |
| ^†^ Self-reported taking blood pressure medication and/or taking ACE inhibitors, beta-blockers, diuretics, or calcium channel blockers. | | | | | | |
| MI = myocardial infarction; IHD = ischaemic heart disease; TIA = transient ischaemic attack; BMI = body mass index; SBP = systolic blood pressure; RHR = heart rate; SD = standard deviation. | | | | | | |

# Table S6: Reporting and diagnostic accuracy of all reported IHD cases by selected characteristics

| **Demographic & Reporting sources** | **No. of reported cases^**^** | **Retrieved cases** | | **Verified cases** | | **Adjudication-confirmed cases** | |
| --- | --- | --- | --- | --- | --- | --- | --- |
|  |  | **No.** | **%** | **No.** | **Reporting accuracy (PPV, 95% CI)** | **No.** | **Diagnostic accuracy (PPV, 95% CI)^*^** |
| **Age at diagnosis** |  |  |  |  |  |  |  |
| <60 | 12,324 | 9,353 | 75.9 | 7,680 | 82.1 (81.3, 82.9) | 5,994 | 92.3 (91.7, 93.0) |
| 60 - 69 | 12,502 | 10,410 | 83.3 | 9,016 | 86.6 (86.0, 87.3) | 6,642 | 93.4 (92.8, 94.0) |
| 70 - 79 | 10,703 | 9,189 | 85.9 | 8,025 | 87.3 (86.7, 88.0) | 5,687 | 93.4 (92.7, 94.0) |
| 80+ | 1,630 | 1,447 | 88.8 | 1,281 | 88.5 (86.9, 90.2) | 812 | 93.8 (92.2, 95.4) |
| **Sex** |  |  |  |  |  |  |  |
| Male | 14,892 | 12,137 | 81.5 | 10,332 | 85.1 (84.5, 85.8) | 7,760 | 93.6 (93.1, 94.1) |
| Female | 22,267 | 18,262 | 82.0 | 15,670 | 85.8 (85.3, 86.3) | 11,375 | 92.7 (92.3, 93.2) |
| **Region** |  |  |  |  |  |  |  |
| Rural | 18,387 | 13,009 | 70.8 | 10,820 | 83.2 (82.5, 83.8) | 7,004 | 91.0 (90.4, 91.6) |
| Urban | 18,772 | 17,390 | 92.6 | 15,182 | 87.3 (86.8, 87.8) | 12,131 | 94.3 (93.9, 94.7) |
| **Reporting source** |  |  |  |  |  |  |  |
| Disease registry | 7,528 | 5,677 | 75.4 | 4,767 | 84.0 (83.0, 84.9) | 3,214 | 91.1 (90.2, 92.1) |
| Health insurance | 29,631 | 24,722 | 83.4 | 21,235 | 85.9 (85.5, 86.3) | 15,921 | 93.5 (93.1, 93.8) |
| **Year of diagnosis** |  |  |  |  |  |  |  |
| <2008 | 2,838 | 2,152 | 75.8 | 1,388 | 64.5 (62.5, 66.5) | 945 | 89.6 (87.7, 91.4) |
| 2008 | 2,656 | 1,920 | 72.3 | 1,548 | 80.6 (78.9, 82.4) | 1,172 | 90.6 (89.0, 92.2) |
| 2009 | 2,621 | 1,903 | 72.6 | 1,585 | 83.3 (81.6, 85.0) | 1,216 | 91.5 (90.0, 93.0) |
| 2010 | 2,757 | 2,064 | 74.9 | 1,793 | 86.9 (85.4, 88.3) | 1,395 | 91.0 (89.6, 92.4) |
| 2011 | 3,525 | 2,489 | 70.6 | 2,093 | 84.1 (82.7, 85.5) | 1,642 | 93.0 (91.8, 94.2) |
| 2012 | 3,788 | 3,038 | 80.2 | 2,555 | 84.1 (82.8, 85.4) | 1,945 | 92.8 (91.7, 93.9) |
| 2013 | 4,152 | 3,538 | 85.2 | 3,124 | 88.3 (87.2, 89.4) | 2,417 | 94.6 (93.8, 95.5) |
| 2014 | 4,638 | 4,057 | 87.5 | 3,692 | 91.0 (90.1, 91.9) | 2,627 | 96.2 (95.4, 96.9) |
| 2015 | 4,348 | 3,935 | 90.5 | 3,523 | 89.5 (88.6, 90.5) | 2,703 | 93.5 (92.6, 94.4) |
| >2015 | 5,836 | 5,303 | 90.9 | 4,701 | 88.6 (87.8, 89.5) | 3,073 | 92.8 (91.9, 93.6) |
| **Hospital tier** |  |  |  |  |  |  |  |
| 0^†^ | 2,671 | 1,439 | 53.9 | 1,085 | 75.4 (73.2, 77.6) | 583 | 92.5 (90.5, 94.6) |
| 1 | 10,762 | 7,791 | 72.4 | 6,495 | 83.4 (82.5, 84.2) | 3,998 | 92.6 (91.8, 93.4) |
| 2 | 6,288 | 5,221 | 83.0 | 4,497 | 86.1 (85.2, 87.1) | 3,505 | 92.7 (91.8, 93.5) |
| 3 | 17,438 | 15,948 | 91.5 | 13,925 | 87.3 (86.8, 87.8) | 11,049 | 93.4 (93.0, 93.8) |
| **All** | **37,159** | **30,399** | **81.8** | **26,002** | **85.5 (85.1, 85.9)** | **19,135** | **93.1 (92.7, 93.4)** |
| PPV = positive predictive value | | | | | | | |
| ^*^ cases with secondary diagnosis of IHD excluded from diagnostic accuracy assessment | | | | | | | |
| ^**^ reported IHD cases with accessible records | | | | | | | |
| ^†^ includes hospitals of unclassified tier | | | | | | | |

# Table S7: Reporting and diagnostic accuracy of hospitalised MI cases by year of diagnosis

|  | **No. of reported cases^†^** | **Retrieved cases** | | **Verified cases** | | **Adjudication-confirmed cases** | | |
| --- | --- | --- | --- | --- | --- | --- | --- | --- |
|  |  | **No.** | **%** | **No.** | **Reporting accuracy (PPV, 95% CI)** | **No.** | **Diagnostic accuracy (PPV, 95% CI)^*^** | **P-value^**^** |
| <2008 | 249 | 140 | 56.2 | 122 | 87.1 (81.6, 92.7) | 109 | 99.1 (97.3, 100.9) | 0.20 |
| 2008 | 236 | 176 | 74.6 | 160 | 90.9 (86.7, 95.2) | 141 | 95.3 (91.9, 98.7) |  |
| 2009 | 209 | 142 | 67.9 | 128 | 90.1 (85.2, 95.0) | 112 | 97.4 (94.5, 100.3) |  |
| 2010 | 215 | 144 | 67.0 | 127 | 88.2 (82.9, 93.5) | 110 | 97.3 (94.4, 100.3) |  |
| 2011 | 219 | 147 | 67.1 | 127 | 86.4 (80.9, 91.9) | 109 | 96.5 (93.1, 99.9) |  |
| 2012 | 236 | 181 | 76.7 | 159 | 87.8 (83.1, 92.6) | 141 | 98.6 (96.7, 100.5) |  |
| 2013 | 330 | 263 | 79.7 | 242 | 92.0 (88.7, 95.3) | 208 | 96.7 (94.4, 99.1) |  |
| 2014 | 291 | 230 | 79.0 | 208 | 90.4 (86.6, 94.2) | 187 | 97.9 (95.9, 99.9) |  |
| 2015 | 285 | 228 | 80.0 | 210 | 92.1 (88.6, 95.6) | 192 | 99.5 (98.5, 100.5) |  |
| >2015 | 350 | 288 | 82.3 | 249 | 86.5 (82.5, 90.4) | 198 | 98.0 (96.1, 99.9) |  |
| **All** | **2,620** | **1,939** | **74.0** | **1,732** | **89.3 (87.9, 90.7)** | **1,507** | **97.7 (96.9, 98.4)** |  |
| MI = myocardial infarction; PPV = positive predictive value | | | | | | | | |
| ^*^ cases with secondary diagnosis of IHD excluded from diagnostic accuracy assessment | | | | | | | | |
| ^**^ P-value for test of equal proportions | | | | | | | | |
| ^†^ reported IHD cases with accessible records | | | | | | | | |

# Table S8: Accuracy of reported and verified hospitalised IHD events by subtype at (a) verification and (b) adjudication

|  | **Reported cases, N (%)** | | **(a) Unverified^*^/ (b) Refuted^**^** | **Total** |
| --- | --- | --- | --- | --- |
|  | **MI** | **Non-MI** | **No. (%)** | **No.** |
| **(a) Verified types** | | | | |
| Reported type^†^ |  |  |  |  |
| MI | **1,066 (55%)** | 666 (34%) | 207 (11%) | 1,939 |
| Non-MI | 1,356 (5%) | **22,914 (81%)** | 4,190 (15%) | 28,460 |
| Any IHD | 2,422 (8%) | 23,580 (78%) | 4,397 (14%) | 30,399 |
| **(b) Adjudication-confirmed types** | | | | |
| Verified type^‡^ |  |  |  |  |
| MI | **1,909 (84%)** | 348 (15%) | 28 (1%) | 2,285 |
| Non-MI | 527 (3%) | **16,351 (89%)** | 1,398 (8%) | 18,276 |
| Any IHD | 2,436 (12%) | 16,699 (81%) | 1,426 (7%) | 20,561 |
| IHD = ischaemic heart disease; MI = myocardial infarction; PPV = positive predictive value | | | | |
| Non-MI includes non-MI acute IHD and chronic IHD cases. | | | | |
| ^*^ no evidence of IHD on review of medical records by public health staff | | | | |
| ^**^ no clinical evidence of IHD on independent adjudication by expert clinicians | | | | |
| ^†^ reported IHD cases with accessible records | | | | |
| ^‡^ cases with secondary diagnosis of IHD excluded from diagnostic accuracy assessment | | | | |

# Table S9: Reporting and diagnostic accuracy of hospitalised IHD cases by area

|  | **No. of reported cases^†^** | **Retrieved cases** | | **Verified cases** | | **Adjudication-confirmed cases** | | |
| --- | --- | --- | --- | --- | --- | --- | --- | --- |
|  |  | **No.** | **%** | **No.** | **Reporting accuracy (PPV, 95% CI)** | **No.** | **Diagnostic accuracy (PPV, 95% CI)^*^** | **P-value^**^** |
| **Urban** | **18,772** | **17,390** | **92.6** | **15,182** | **87.3 (86.8, 87.8)** | **12,131** | 94.3 (93.9, 94.7) | <0.0001 |
| Qingdao | 3,181 | 2,969 | 93.3 | 2,840 | 95.7 (94.9, 96.4) | 2,412 | 94.5 (93.6, 95.4) |  |
| Harbin | 9,404 | 8,799 | 93.6 | 7,679 | 87.3 (86.6, 88.0) | 6,131 | 94.4 (93.9, 95.0) |  |
| Haikou | 1,481 | 1,360 | 91.8 | 1,200 | 88.2 (86.5, 89.9) | 937 | 94.2 (92.7, 95.6) |  |
| Suzhou | 1,086 | 834 | 76.8 | 628 | 75.3 (72.4, 78.2) | 469 | 90.4 (87.8, 92.9) |  |
| Liuzhou | 3,620 | 3,428 | 94.7 | 2,835 | 82.7 (81.4, 84.0) | 2,182 | 94.7 (93.8, 95.6) |  |
| **Rural** | **18,387** | **13,009** | **70.8** | **10,820** | **83.2 (82.5, 83.8)** | **7,004** | 91.0 (90.4, 91.6) |  |
| Sichuan | 3,618 | 2,861 | 79.1 | 2,402 | 84.0 (82.6, 85.3) | 801 | 88.8 (86.7, 90.9) |  |
| Gansu | 2,645 | 1,419 | 53.6 | 1,114 | 78.5 (76.4, 80.6) | 830 | 91.7 (89.9, 93.5) |  |
| Henan | 5,293 | 4,102 | 77.5 | 3,535 | 86.2 (85.1, 87.2) | 2,749 | 91.2 (90.2, 92.2) |  |
| Zhejiang | 1,977 | 1,203 | 60.8 | 838 | 69.7 (67.1, 72.3) | 573 | 84.6 (81.9, 87.4) |  |
| Hunan | 4,854 | 3,424 | 70.5 | 2,931 | 85.6 (84.4, 86.8) | 2,051 | 93.3 (92.3, 94.4) |  |
| IHD = ischaemic heart disease; PPV = positive predictive value | | | | | | | | |
| ^*^ cases with secondary diagnosis of IHD excluded from diagnostic accuracy assessment | | | | | | | | |
| ^**^ P-value for test of equal proportions | | | | | | | | |
| ^†^ reported IHD cases with accessible records | | | | | | | | |

# Table S10: Hospital discharge diagnoses among unverified IHD cases by reported IHD subtype

|  | **Reported IHD type (N, %)** | | |
| --- | --- | --- | --- |
| **Hospital discharge diagnoses** | **MI** | **Non-MI** | **All IHD** |
| **Non-IHD cardiac disease** |  |  |  |
| Heart failure | 1 (0) | 36 (1) | 37 (1) |
| Atrial fibrillation | 2 (1) | 25 (1) | 27 (1) |
| Other arrhythmia | 9 (4) | 212 (5) | 221 (5) |
| Cardiomyopathy | 3 (1) | 58 (1) | 61 (1) |
| Valvular heart disease | 1 (0) | 112 (3) | 113 (3) |
| Pulmonary heart disease | 1 (0) | 45 (1) | 46 (1) |
| Cardiac arrest | 0 (0) | 1 (0) | 1 (0) |
| Other non-IHD cardiac disease | 14 (7) | 416 (10) | 430 (10) |
| **Cerebrovascular disease** | **102 (49)** | **1,509 (36)** | **1,611 (37)** |
| **Hypertensive disease** | **0 (0)** | **93 (2)** | **93 (2)** |
| **Other circulatory disease** | **1 (0)** | **36 (1)** | **37 (1)** |
| **Non-circulatory disease** | **73 (35)** | **1,647 (39)** | **1,720 (39)** |
| **Total** | **207 (100)** | **4,190 (100)** | **4,397 (100)** |
| Hierarchy: Heart failure > Atrial fibrillation > Other arrhythmia > Cardiomyopathy > Valvular heart disease > Pulmonary heart disease > Cardiac arrest > Other non-IHD cardiac disease > Cerebrovascular disease > Hypertensive disease > Other circulatory disease > Non-circulatory disease | | | |
| IHD = ischaemic heart disease | | | |

# Table S11: Non-circulatory disease diagnoses among unverified IHD cases

| **Diagnosis** | **Number of cases** |
| --- | --- |
| J44: Other chronic obstructive pulmonary disease | 163 |
| N28: Other disorders of kidney and ureter, not elsewhere classified | 76 |
| K29: Gastritis and duodenitis | 72 |
| E13: Other specified diabetes mellitus | 64 |
| C34: Malignant neoplasm of bronchus and lung | 59 |
| G45: Transient cerebral ischaemic attacks and related syndromes | 57 |
| E11: Non-insulin-dependent diabetes mellitus | 49 |
| J18: Pneumonia, organism unspecified | 44 |
| J98: Other respiratory disorders | 43 |
| C16: Malignant neoplasm of stomach | 36 |
| C15: Malignant neoplasm of oesophagus | 34 |
| C18: Malignant neoplasm of colon | 33 |
| J20: Acute bronchitis | 33 |
| C22: Malignant neoplasm of liver and intrahepatic bile ducts | 32 |
| M51: Other intervertebral disc disorders | 32 |
| M47: Spondylosis | 31 |
| D25: Leiomyoma of uterus | 29 |
| K81: Cholecystitis | 29 |
| C50: Malignant neoplasm of breast | 28 |
| N64: Other disorders of breast | 28 |
| G98: Other disorders of nervous system, not elsewhere classified | 26 |
| F45: Somatoform disorders | 24 |
| Q24: Other congenital malformations of heart | 23 |
| C53: Malignant neoplasm of cervix uteri | 21 |
| J42: Unspecified chronic bronchitis | 21 |
| C20: Malignant neoplasm of rectum | 19 |
| N19: Unspecified kidney failure | 19 |
| R69: Unknown and unspecified causes of morbidity | 19 |
| A15: Respiratory tuberculosis, bacteriologically and histologically confirmed | 18 |
| N39: Other disorders of urinary system | 18 |
| A09: Other gastroenteritis and colitis of infectious and unspecified origin | 17 |
| J06: Acute upper respiratory infections of multiple and unspecified sites | 17 |
| K25: Gastric ulcer | 17 |
| K80: Cholelithiasis | 17 |
| E78: Disorders of lipoprotein metabolism and other lipidaemias | 16 |
| K64: No codable diagnosis | 16 |
| C76: Malignant neoplasm of other and ill-defined sites | 14 |
| K83: Other diseases of biliary tract | 14 |
| K92: Other diseases of digestive system | 13 |
| N18: Chronic kidney disease | 13 |
| B88: Other infestations | 12 |
| D48: Neoplasm of uncertain or unknown behaviour of other and unspecified sites | 12 |
| H26: Other cataract | 12 |
| K35: Acute appendicitis | 12 |
| J45: Asthma | 11 |
| K40: Inguinal hernia | 11 |
| J40: Bronchitis, not specified as acute or chronic | 10 |
| Only diagnoses with 10 or more cases are shown. | |

# Table S12: Physician-adjudicated diagnoses among refuted IHD cases by discharge diagnosis

|  | **IHD diagnosis at discharge (N, %)** | | |
| --- | --- | --- | --- |
| **Physician-adjudicated diagnoses** | **MI** | **Non-MI** | **All IHD** |
| **Non-IHD cardiac disease** |  |  |  |
| Heart failure | 1 (4) | 73 (5) | 74 (5) |
| Atrial fibrillation | 0 (0) | 143 (10) | 143 (10) |
| Other arrhythmia | 2 (7) | 147 (11) | 149 (10) |
| Cardiomyopathy | 0 (0) | 4 (0) | 4 (0) |
| Valvular heart disease | 1 (4) | 14 (1) | 15 (1) |
| Pulmonary heart disease | 1 (4) | 8 (1) | 9 (1) |
| Cardiac arrest | 0 (0) | 0 (0) | 0 (0) |
| Other non-IHD cardiac disease | 1 (4) | 53 (4) | 54 (4) |
| **Cerebrovascular disease** | **5 (18)** | **204 (15)** | **209 (15)** |
| **Hypertensive disease** | **1 (4)** | **44 (3)** | **45 (3)** |
| **Other circulatory disease** | **0 (0)** | **3 (0)** | **3 (0)** |
| **Non-circulatory disease** | **16 (57)** | **705 (50)** | **721 (51)** |
| **Total** | **28 (100)** | **1,398 (100)** | **1,426 (100)** |
| Hierarchy: Heart failure > Atrial fibrillation > Other arrhythmia > Cardiomyopathy > Valvular heart disease > Pulmonary heart disease > Cardiac arrest > Other non-IHD cardiac disease > Cerebrovascular disease > Hypertensive disease > Other circulatory disease > Non-circulatory disease | | | |
| IHD = ischaemic heart disease | | | |

# Table S13: Non-circulatory disease diagnoses among refuted IHD cases

| **Diagnosis** | **Number of cases** |
| --- | --- |
| No ICD-10 codable diagnosis | 568 |
| R90: Abnormal findings on diagnostic imaging of central nervous system | 34 |
| J98: Other respiratory disorders | 20 |
| R69: Unknown and unspecified causes of morbidity | 18 |
| K29: Gastritis and duodenitis | 17 |
| J18: Pneumonia, organism unspecified | 9 |
| J42: Unspecified chronic bronchitis | 6 |
| J44: Other chronic obstructive pulmonary disease | 6 |
| J22: Unspecified acute lower respiratory infection | 3 |
| J45: Asthma | 3 |
| A09: Other gastroenteritis and colitis of infectious and unspecified origin | 2 |
| B02: Zoster [herpes zoster] | 2 |
| D64: Other anaemias | 2 |
| F45: Somatoform disorders | 2 |
| J04: Acute laryngitis and tracheitis | 2 |
| J20: Acute bronchitis | 2 |
| K25: Gastric ulcer | 2 |
| K74: Fibrosis and cirrhosis of liver | 2 |
| C16: Malignant neoplasm of stomach | 1 |
| C34: Malignant neoplasm of bronchus and lung | 1 |
| E03: Other hypothyroidism | 1 |
| E05: Thyrotoxicosis [hyperthyroidism] | 1 |
| E11: Non-insulin-dependent diabetes mellitus | 1 |
| E87: Other disorders of fluid, electrolyte and acid-base balance | 1 |
| F41: Other anxiety disorders | 1 |
| F48: Other neurotic disorders | 1 |
| G90: Disorders of autonomic nervous system | 1 |
| H81: Disorders of vestibular function | 1 |
| J40: Bronchitis, not specified as acute or chronic | 1 |
| J47: Bronchiectasis | 1 |
| J90: Pleural effusion, not elsewhere classified | 1 |
| K27: Peptic ulcer, site unspecified | 1 |
| K31: Other diseases of stomach and duodenum | 1 |
| K76: Other diseases of liver | 1 |
| K92: Other diseases of digestive system | 1 |
| L50: Urticaria | 1 |
| M47: Spondylosis | 1 |
| M75: Shoulder lesions | 1 |
| N20: Calculus of kidney and ureter | 1 |
| R09: Other symptoms and signs involving the circulatory and respiratory systems | 1 |
| R42: Dizziness and giddiness | 1 |
| T78: Adverse effects, not elsewhere classified | 1 |

# Table S14: Reporting and diagnostic accuracy of all IHD cases reported by health insurance and disease registries including participants with prior IHD at baseline

|  | **No. of reported cases** | **Retrieved cases** | | **Verified cases** | | **Adjudication-confirmed cases** | | |
| --- | --- | --- | --- | --- | --- | --- | --- | --- |
|  |  | **No.** | **%** | **No.** | **Reporting accuracy (PPV, 95% CI)** | **No.** | **Diagnostic accuracy (PPV, 95% CI)^*^** | **P-value^**^** |
| All IHD cases | 43,475 | 36,082 | 83.0 | 31,268 | 86.7 (86.3, 87.0) | 23,208 | 93.3 (92.9, 93.6) | 0.0057 |
| No self-reported IHD at baseline | 37,159 | 30,399 | 81.8 | 26,002 | 85.5 (85.1, 85.9) | 19,135 | 93.1 (92.7, 93.4) |  |
| Self-reported IHD at baseline | 6,310 | 5,677 | 90.0 | 5,261 | 92.7 (92.0, 93.4) | 4,073 | 94.2 (93.5, 94.9) |  |
| PPV = positive predictive value; IHD = ischaemic heart disease | | | | | | | | |
| ^*^ cases with secondary diagnosis of IHD excluded from diagnostic accuracy assessment | | | | | | | | |
| ^**^ P-value for test of equal proportions | | | | | | | | |

# Table S15: Performance of medical tests

|  | | **Tests performed, n (%)** | |
| --- | --- | --- | --- |
|  | **No. of adjudication-confirmed cases** | **Cardiac biomarkers^*^** | **ECG** |
| **Reported IHD subtype** |  |  |  |
| MI | 2,436 | 2,212 (90.8) | 2,401 (98.6) |
| Non-MI | 16,699 | 10,588 (63.4) | 16,286 (97.5) |
| **Sex** |  |  |  |
| Men | 7,760 | 5,600 (72.2) | 7,592 (97.8) |
| Women | 11,375 | 7,200 (63.3) | 11,095 (97.5) |
| **Region** |  |  |  |
| Rural | 7,004 | 4,130 (59.0) | 6,797 (97.0) |
| Urban | 12,131 | 8,670 (71.5) | 11,890 (98.0) |
| **Hospital tier** |  |  |  |
| 0 | 583 | 361 (61.9) | 564 (96.7) |
| 1 | 3,998 | 1,561 (39.0) | 3,855 (96.4) |
| 2 | 3,505 | 2,283 (65.1) | 3,441 (98.2) |
| 3 | 11,049 | 8,595 (77.8) | 10,827 (98.0) |
| **Year** |  |  |  |
| <2008 | 945 | 500 (52.9) | 921 (97.5) |
| 2008 | 1,172 | 647 (55.2) | 1,147 (97.9) |
| 2009 | 1,216 | 687 (56.5) | 1,179 (97.0) |
| 2010 | 1,395 | 831 (59.6) | 1,362 (97.6) |
| 2011 | 1,642 | 1,052 (64.1) | 1,604 (97.7) |
| 2012 | 1,945 | 1,268 (65.2) | 1,896 (97.5) |
| 2013 | 2,417 | 1,703 (70.5) | 2,349 (97.2) |
| 2014 | 2,627 | 1,874 (71.3) | 2,563 (97.6) |
| 2015 | 2,703 | 1,949 (72.1) | 2,643 (97.8) |
| >2015 | 3,073 | 2,289 (74.5) | 3,023 (98.4) |
| **All** | 19,135 | 12,800 (66.9) | 18,687 (97.7) |
| IHD = ischaemic heart disease; MI = myocardial infarction; ECG = electrocardiogram | | | |
| Non-MI includes non-MI acute IHD and chronic IHD cases. | | | |
| ^*^ cardiac enzymes and/or troponin testing | | | |

# Table S16: Use of standard medication, revascularisation and traditional Chinese medicine by IHD subtypes and sex

|  | **MI** | | **Non-MI^*^** | |
| --- | --- | --- | --- | --- |
| **Medications, n (%)** | **Men (n=1,571)** | **Women (n=865)** | **Men (n=6,189)** | **Women (n=10,510)** |
| **Standard medication** |  |  |  |  |
| Antiplatelet^**^ | 1,370 (87.2) | 694 (80.2) | 4,316 (69.7) | 6,406 (61.0) |
| Lipid-lowering^†^ | 1,246 (79.3) | 637 (73.6) | 3,736 (60.4) | 5,667 (53.9) |
| Antihypertensive^‡^ | 1,166 (74.2) | 642 (74.2) | 3,961 (64.0) | 6,168 (58.7) |
| Anticoagulant^§^ | 931 (59.3) | 421 (48.7) | 1,231 (19.9) | 1,229 (11.7) |
| Any standard medication | 1,460 (92.9) | 777 (89.8) | 5,231 (84.5) | 8,366 (79.6) |
| **Revascularisation** |  |  |  |  |
| Thrombolysis | 55 (3.5) | 26 (3.0) | 10 (0.2) | 12 (0.1) |
| Any coronary intervention^\|^ | 601 (38.3) | 226 (26.1) | 586 (9.5) | 430 (4.1) |
| Any revascularisation^¶^ | 643 (40.9) | 248 (28.7) | 596 (9.6) | 442 (4.2) |
| **Traditional Chinese Medication** | 933 (59.4) | 515 (59.5) | 4,242 (68.5) | 7,622 (72.5) |
| Abbreviations: IHD = ischaemic heart disease; MI = myocardial infarction | | | | |
| ^*^ includes non-MI acute IHD and chronic IHD cases | | | | |
| ^**^ aspirin, clopidogrel, or GP IIb/IIIa antagonist therapy | | | | |
| ^†^ statin and non-statin lipid-lowering therapy | | | | |
| ^‡^ angiotensin converting enzyme inhibitor, angiotensin receptor blocker, beta blocker, calcium antagonist, or diuretic therapy | | | | |
| ^§^ low molecular weight heparin or unfractionated heparin therapy | | | | |
| ^\|^ percutaneous coronary intervention (PCI) or coronary artery bypass graft (CABG) | | | | |
| ^¶^ some cases received both thrombolytic therapy and any procedure | | | | |
| MI = myocardial infarction; IHD = ischaemic heart disease | | | | |
| Combined treatments from first 24 hours after admission, during hospital stay, and at discharge. | | | | |

# Table S17: Use of standard medication, revascularisation and traditional Chinese medicine by IHD subtypes and area

|  | **MI** | | **Non-MI^*^** | |
| --- | --- | --- | --- | --- |
| **Medications, n (%)** | **Rural (n=880)** | **Urban (n=1,556)** | **Rural (n=6,124)** | **Urban (n=10,575)** |
| **Standard medication** |  |  |  |  |
| Antiplatelet^**^ | 712 (80.9) | 1,352 (86.9) | 3,212 (52.4) | 7,510 (71.0) |
| Lipid-lowering^†^ | 631 (71.7) | 1,252 (80.5) | 2,863 (46.8) | 6,540 (61.8) |
| Antihypertensive^‡^ | 622 (70.7) | 1,186 (76.2) | 3,662 (59.8) | 6,467 (61.2) |
| Anticoagulant^§^ | 488 (55.5) | 864 (55.5) | 806 (13.2) | 1,654 (15.6) |
| Any standard medication | 793 (90.1) | 1,444 (92.8) | 4,687 (76.5) | 8,910 (84.3) |
| **Revascularisation** |  |  |  |  |
| Thrombolysis | 43 (4.9) | 38 (2.4) | 10 (0.2) | 12 (0.1) |
| Any coronary intervention^\|^ | 143 (16.2) | 684 (44.0) | 160 (2.6) | 856 (8.1) |
| Any revascularisation^¶^ | 182 (20.7) | 709 (45.6) | 170 (2.8) | 868 (8.2) |
| **Traditional Chinese Medication** | 578 (65.7) | 870 (55.9) | 4,564 (74.5) | 7,300 (69.0) |
| Abbreviations: IHD = ischaemic heart disease; MI = myocardial infarction | | | | |
| ^*^ includes non-MI acute IHD and chronic IHD cases | | | | |
| ^**^ aspirin, clopidogrel, or GP IIb/IIIa antagonist therapy | | | | |
| ^†^ statin and non-statin lipid-lowering therapy | | | | |
| ^‡^ angiotensin converting enzyme inhibitor, angiotensin receptor blocker, beta blocker, calcium antagonist, or diuretic therapy | | | | |
| ^§^ low molecular weight heparin or unfractionated heparin therapy | | | | |
| ^\|^ percutaneous coronary intervention (PCI) or coronary artery bypass graft (CABG) | | | | |
| ^¶^ some cases received both thrombolytic therapy and any procedure | | | | |
| MI = myocardial infarction; IHD = ischaemic heart disease | | | | |
| Combined treatments from first 24 hours after admission, during hospital stay, and at discharge. | | | | |

# Table S18: Use of standard medication, revascularisation and traditional Chinese medicine by IHD subtypes and hospital tier

|  | **MI** | | | | **Non-MI** | | | |
| --- | --- | --- | --- | --- | --- | --- | --- | --- |
| **Medications, n (%)** | **Tier: 0 (n=48)^††^** | **1 (n=228)** | **2 (n=370)** | **3 (n=1,790)** | **Tier: 0 (n=535)** | **1 (n=3,770)** | **2 (n=3,135)** | **3 (n=9,259)** |
| **Standard medication** |  |  |  |  |  |  |  |  |
| Antiplatelet^**^ | 36 (75.0) | 156 (68.4) | 305 (82.4) | 1,567 (87.5) | 298 (55.7) | 1,455 (38.6) | 2,032 (64.8) | 6,937 (74.9) |
| Lipid-lowering^†^ | 34 (70.8) | 133 (58.3) | 258 (69.7) | 1,458 (81.5) | 246 (46.0) | 1,179 (31.3) | 1,664 (53.1) | 6,314 (68.2) |
| Antihypertensive^‡^ | 32 (66.7) | 133 (58.3) | 270 (73.0) | 1,373 (76.7) | 300 (56.1) | 1,816 (48.2) | 1,981 (63.2) | 6,032 (65.1) |
| Anticoagulant^§^ | 19 (39.6) | 110 (48.2) | 185 (50.0) | 1,038 (58.0) | 43 (8.0) | 334 (8.9) | 427 (13.6) | 1,656 (17.9) |
| Any standard medication | 39 (81.2) | 187 (82.0) | 333 (90.0) | 1,678 (93.7) | 403 (75.3) | 2,460 (65.3) | 2,584 (82.4) | 8,150 (88.0) |
| **Revascularisation** |  |  |  |  |  |  |  |  |
| Thrombolysis | 1 (2.1) | 11 (4.8) | 22 (5.9) | 47 (2.6) | 1 (0.2) | 4 (0.1) | 5 (0.2) | 12 (0.1) |
| Any coronary intervention^\|^ | 10 (20.8) | 12 (5.3) | 71 (19.2) | 734 (41.0) | 28 (5.2) | 22 (0.6) | 92 (2.9) | 874 (9.4) |
| Any revascularisation^¶^ | 11 (22.9) | 23 (10.1) | 91 (24.6) | 766 (42.8) | 29 (5.4) | 26 (0.7) | 97 (3.1) | 886 (9.6) |
| **Traditional Chinese Medication** | 29 (60.4) | 161 (70.6) | 218 (58.9) | 1,040 (58.1) | 376 (70.3) | 2,866 (76.0) | 2,350 (75.0) | 6,272 (67.7) |
| Abbreviations: IHD = ischaemic heart disease; MI = myocardial infarction | | | | | | | | |
| ^*^ includes non-MI acute IHD and chronic IHD cases | | | | | | | | |
| ^**^ aspirin, clopidogrel, or GP IIb/IIIa antagonist therapy | | | | | | | | |
| ^†^ statin and non-statin lipid-lowering therapy | | | | | | | | |
| ^‡^ angiotensin converting enzyme inhibitor, angiotensin receptor blocker, beta blocker, calcium antagonist, or diuretic therapy | | | | | | | | |
| ^§^ low molecular weight heparin or unfractionated heparin therapy | | | | | | | | |
| ^\|^ percutaneous coronary intervention (PCI) or coronary artery bypass graft (CABG) | | | | | | | | |
| ^¶^ some cases received both thrombolytic therapy and any procedure | | | | | | | | |
| ^††^ includes hospitals of unclassified tier | | | | | | | | |
| MI = myocardial infarction; IHD = ischaemic heart disease | | | | | | | | |
| Combined treatments from first 24 hours after admission, during hospital stay, and at discharge. | | | | | | | | |

# Table S19: Use of standard medication and revascularisation by IHD subtypes by reporting year and age at diagnosis

|  | **No. (%) with any standard medication** | | **No. (%) with any revascularisation** | |
| --- | --- | --- | --- | --- |
|  | **MI** | **Non-MI** | **MI** | **Non-MI** |
| **Year** |  |  |  |  |
| <2008 | 133 (91.7) | 646 (80.8) | 49 (33.8) | 52 (6.5) |
| 2008 | 149 (83.2) | 817 (82.3) | 56 (31.3) | 86 (8.7) |
| 2009 | 156 (83.4) | 828 (80.5) | 73 (39.0) | 80 (7.8) |
| 2010 | 163 (91.6) | 995 (81.8) | 76 (42.7) | 90 (7.4) |
| 2011 | 181 (90.0) | 1,231 (85.4) | 86 (42.8) | 98 (6.8) |
| 2012 | 238 (95.6) | 1,410 (83.1) | 83 (33.3) | 91 (5.4) |
| 2013 | 292 (91.0) | 1,743 (83.2) | 106 (33.0) | 119 (5.7) |
| 2014 | 318 (95.2) | 1,877 (81.9) | 128 (38.3) | 104 (4.5) |
| 2015 | 299 (94.6) | 1,914 (80.2) | 108 (34.2) | 142 (5.9) |
| >2015 | 308 (94.5) | 2,136 (77.8) | 126 (38.7) | 176 (6.4) |
| **Age at diagnosis, years** |  |  |  |  |
| 30 - 39 | 7 (100.0) | 8 (47.1) | 4 (57.1) | 3 (17.6) |
| 40 - 49 | 139 (93.9) | 709 (68.6) | 71 (48.0) | 50 (4.8) |
| 50 - 59 | 451 (90.4) | 3,234 (77.9) | 227 (45.5) | 229 (5.5) |
| 60 - 69 | 754 (92.2) | 4,795 (83.4) | 314 (38.4) | 412 (7.2) |
| 70 - 79 | 749 (91.3) | 4,225 (84.3) | 247 (30.1) | 307 (6.1) |
| 80 - 89 | 137 (95.1) | 626 (84.9) | 28 (19.4) | 37 (5.0) |
| **Total** | 2,237 (91.8) | 13,597 (81.4) | 891 (36.6) | 1,038 (6.2) |
| Any standard medication includes antiplatelet, lipid-lowering, antihypertensive, and anticoagulant agents. | | | | |
| Any revascularisation includes thrombolysis, percutaneous coronary intervention (PCI) or coronary artery bypass graft (CABG). | | | | |
| Following MI, rates of any revascularisation in tier 3 hospitals by year were 40.4% (<2008), 39.8%, 45.4%, 43.9%, 45.3%, 37.6%, 42.1%, 46.8%, 41.1%, and 43.6% (>2015). | | | | |

# Table S20: Cumulative event rates of recurrent MI events, stroke, heart failure and all-cause mortality from 28 days following first adjudicated event of different IHD subtypes

## (a) after first MI

|  | **Years since first MI (n = 2,481)** | | | | | | | | | | |
| --- | --- | --- | --- | --- | --- | --- | --- | --- | --- | --- | --- |
|  | **28 days** | **1** | **2** | **3** | **4** | **5** | **6** | **7** | **8** | **9** | **10** |
| **Recurrent MI event** |  |  |  |  |  |  |  |  |  |  |  |
| No. events | 0 | 132 | 177 | 211 | 232 | 263 | 281 | 293 | 298 | 299 | 304 |
| No. free of any events and death | 2,158 | 1,922 | 1,555 | 1,261 | 970 | 681 | 474 | 317 | 210 | 115 | 45 |
| No. deaths | 0 | 101 | 163 | 200 | 240 | 268 | 290 | 301 | 312 | 316 | 318 |
| No. censored | 0 | 3 | 263 | 486 | 716 | 946 | 1,113 | 1,247 | 1,338 | 1,428 | 1,491 |
| Cumulative event rate, % (95% CI) | 0 | 6 (5-7) | 8 (7-10) | 10 (9-12) | 12 (10-13) | 14 (13-16) | 17 (15-19) | 19 (16-21) | 20 (17-22) | 20 (18-23) | 24 (20-28) |
| **Stroke** |  |  |  |  |  |  |  |  |  |  |  |
| No. events | 0 | 92 | 164 | 216 | 255 | 285 | 305 | 326 | 338 | 342 | 344 |
| No. free of any events and death | 2,165 | 1,931 | 1,534 | 1,210 | 919 | 635 | 440 | 288 | 188 | 102 | 40 |
| No. deaths | 0 | 139 | 209 | 254 | 289 | 324 | 347 | 361 | 372 | 376 | 380 |
| No. censored | 0 | 3 | 258 | 485 | 702 | 921 | 1,073 | 1,190 | 1,267 | 1,345 | 1,401 |
| Cumulative event rate, % (95% CI) | 0 | 4 (3-5) | 8 (7-9) | 11 (10-12) | 14 (12-15) | 16 (14-18) | 19 (17-21) | 22 (20-24) | 25 (22-27) | 26 (23-29) | 27 (24-31) |
| **Heart failure** |  |  |  |  |  |  |  |  |  |  |  |
| No. events | 0 | 26 | 42 | 59 | 73 | 79 | 85 | 93 | 99 | 101 | 102 |
| No. free of any events and death | 2,196 | 2,020 | 1,659 | 1,342 | 1,034 | 737 | 526 | 357 | 237 | 137 | 54 |
| No. deaths | 0 | 147 | 227 | 278 | 325 | 365 | 390 | 411 | 425 | 430 | 435 |
| No. censored | 0 | 3 | 268 | 517 | 764 | 1,015 | 1,195 | 1,335 | 1,435 | 1,528 | 1,605 |
| Cumulative event rate, % (95% CI) | 0 | 1 (1-2) | 2 (1-3) | 3 (2-4) | 4 (3-5) | 4 (4-5) | 5 (4-6) | 6 (5-8) | 8 (6-10) | 8 (7-10) | 9 (7-12) |
| **All-cause mortality** |  |  |  |  |  |  |  |  |  |  |  |
| No. events | 0 | 164 | 253 | 315 | 369 | 417 | 450 | 475 | 495 | 501 | 506 |
| No. free of any events and death | 2,220 | 2,053 | 1,687 | 1,373 | 1,066 | 755 | 538 | 371 | 248 | 140 | 57 |
| No. censored | 0 | 3 | 280 | 532 | 785 | 1,048 | 1,232 | 1,374 | 1,477 | 1,579 | 1,657 |
| Cumulative event rate, % (95% CI) | 0 | 7 (6-9) | 12 (10-13) | 15 (14-17) | 19 (17-21) | 23 (21-25) | 27 (25-29) | 31 (28-33) | 35 (32-38) | 37 (34-41) | 40 (36-44) |
| IHD = ischaemic heart disease; MI = myocardial infarction | | | | | | | | | | | |
| Non-MI includes non-MI acute IHD and chronic IHD cases. | | | | | | | | | | | |

## (b) after first non-MI

|  | **Years since first non-MI (n = 16,778)** | | | | | | | | | | |
| --- | --- | --- | --- | --- | --- | --- | --- | --- | --- | --- | --- |
|  | **28 days** | **1** | **2** | **3** | **4** | **5** | **6** | **7** | **8** | **9** | **10** |
| **Recurrent MI event** |  |  |  |  |  |  |  |  |  |  |  |
| No. events | 0 | 224 | 371 | 478 | 551 | 611 | 657 | 690 | 712 | 731 | 737 |
| No. free of any events and death | 16,591 | 15,844 | 12,610 | 10,181 | 7,768 | 5,543 | 3,817 | 2,563 | 1,644 | 916 | 353 |
| No. deaths | 0 | 493 | 816 | 1,061 | 1,265 | 1,417 | 1,530 | 1,610 | 1,670 | 1,701 | 1,719 |
| No. censored | 0 | 30 | 2,794 | 4,871 | 7,007 | 9,020 | 10,587 | 11,728 | 12,565 | 13,243 | 13,782 |
| Cumulative event rate, % (95% CI) | 0 | 1 (1-2) | 2 (2-3) | 3 (3-3) | 4 (4-4) | 5 (4-5) | 6 (5-6) | 6 (6-7) | 7 (7-8) | 8 (7-9) | 9 (8-10) |
| **Stroke** |  |  |  |  |  |  |  |  |  |  |  |
| No. events | 0 | 793 | 1,467 | 1,991 | 2,352 | 2,614 | 2,784 | 2,870 | 2,957 | 2,998 | 3,023 |
| No. free of any events and death | 16,032 | 14,735 | 11,283 | 8,743 | 6,401 | 4,389 | 2,946 | 1,972 | 1,212 | 674 | 259 |
| No. deaths | 0 | 475 | 745 | 940 | 1,086 | 1,199 | 1,280 | 1,320 | 1,359 | 1,387 | 1,401 |
| No. censored | 0 | 29 | 2,537 | 4,358 | 6,193 | 7,830 | 9,022 | 9,870 | 10,504 | 10,973 | 11,349 |
| Cumulative event rate, % (95% CI) | 0 | 5 (5-5) | 10 (9-10) | 14 (13-14) | 18 (17-18) | 21 (20-22) | 24 (23-25) | 26 (25-27) | 30 (29-31) | 32 (31-33) | 35 (33-37) |
| **Heart failure** |  |  |  |  |  |  |  |  |  |  |  |
| No. events | 0 | 129 | 247 | 328 | 391 | 441 | 467 | 493 | 507 | 515 | 516 |
| No. free of any events and death | 16,502 | 15,780 | 12,596 | 10,174 | 7,793 | 5,551 | 3,846 | 2,591 | 1,667 | 942 | 367 |
| No. deaths | 0 | 564 | 918 | 1,197 | 1,406 | 1,581 | 1,706 | 1,793 | 1,863 | 1,902 | 1,921 |
| No. censored | 0 | 29 | 2,741 | 4,803 | 6,912 | 8,929 | 10,483 | 11,625 | 12,465 | 13,143 | 13,698 |
| Cumulative event rate, % (95% CI) | 0 | 1 (1-1) | 2 (1-2) | 2 (2-2) | 3 (3-3) | 3 (3-4) | 4 (4-4) | 5 (4-5) | 5 (5-6) | 6 (5-6) | 6 (5-6) |
| **All-cause mortality** |  |  |  |  |  |  |  |  |  |  |  |
| No. events | 0 | 613 | 1,017 | 1,337 | 1,584 | 1,785 | 1,933 | 2,032 | 2,111 | 2,158 | 2,182 |
| No. free of any events and death | 16,651 | 16,008 | 12,810 | 10,383 | 7,969 | 5,702 | 3,950 | 2,665 | 1,715 | 969 | 374 |
| No. censored | 0 | 30 | 2,824 | 4,931 | 7,098 | 9,164 | 10,768 | 11,954 | 12,825 | 13,524 | 14,095 |
| Cumulative event rate, % (95% CI) | 0 | 4 (3-4) | 6 (6-7) | 9 (8-9) | 11 (11-12) | 14 (13-15) | 17 (16-17) | 19 (18-20) | 22 (21-23) | 25 (23-26) | 27 (26-29) |
| IHD = ischaemic heart disease; MI = myocardial infarction | | | | | | | | | | | |
| Non-MI includes non-MI acute IHD and chronic IHD cases. | | | | | | | | | | | |

## (c) after first IHD

|  | **Years since first IHD (n = 19,259)** | | | | | | | | | | |
| --- | --- | --- | --- | --- | --- | --- | --- | --- | --- | --- | --- |
|  | **28 days** | **1** | **2** | **3** | **4** | **5** | **6** | **7** | **8** | **9** | **10** |
| **Recurrent MI event** |  |  |  |  |  |  |  |  |  |  |  |
| No. events | 0 | 356 | 548 | 689 | 783 | 874 | 938 | 983 | 1,010 | 1,030 | 1,041 |
| No. free of any events and death | 18,749 | 17,766 | 14,165 | 11,442 | 8,738 | 6,224 | 4,291 | 2,880 | 1,854 | 1,031 | 398 |
| No. deaths | 0 | 594 | 979 | 1,261 | 1,505 | 1,685 | 1,820 | 1,911 | 1,982 | 2,017 | 2,037 |
| No. censored | 0 | 33 | 3,057 | 5,357 | 7,723 | 9,966 | 11,700 | 12,975 | 13,903 | 14,671 | 15,273 |
| Cumulative event rate, % (95% CI) | 0 | 2 (2-2) | 3 (3-3) | 4 (4-4) | 5 (4-5) | 6 (5-6) | 7 (6-7) | 8 (7-8) | 9 (8-9) | 10 (9-10) | 11 (10-12) |
| **Stroke** |  |  |  |  |  |  |  |  |  |  |  |
| No. events | 0 | 885 | 1,631 | 2,207 | 2,607 | 2,899 | 3,089 | 3,196 | 3,295 | 3,340 | 3,367 |
| No. free of any events and death | 18,197 | 16,666 | 12,817 | 9,953 | 7,320 | 5,024 | 3,386 | 2,260 | 1,400 | 776 | 299 |
| No. deaths | 0 | 614 | 954 | 1,194 | 1,375 | 1,523 | 1,627 | 1,681 | 1,731 | 1,763 | 1,781 |
| No. censored | 0 | 32 | 2,795 | 4,843 | 6,895 | 8,751 | 10,095 | 11,060 | 11,771 | 12,318 | 12,750 |
| Cumulative event rate, % (95% CI) | 0 | 5 (5-5) | 9 (9-10) | 14 (13-14) | 17 (16-18) | 20 (20-21) | 23 (23-24) | 26 (25-27) | 29 (28-30) | 31 (30-32) | 34 (32-35) |
| **Heart failure** |  |  |  |  |  |  |  |  |  |  |  |
| No. events | 0 | 155 | 289 | 387 | 464 | 520 | 552 | 586 | 606 | 616 | 618 |
| No. free of any events and death | 18,698 | 17,800 | 14,255 | 11,516 | 8,827 | 6,288 | 4,372 | 2,948 | 1,904 | 1,079 | 421 |
| No. deaths | 0 | 711 | 1,145 | 1,475 | 1,731 | 1,946 | 2,096 | 2,204 | 2,288 | 2,332 | 2,356 |
| No. censored | 0 | 32 | 3,009 | 5,320 | 7,676 | 9,944 | 11,678 | 12,960 | 13,900 | 14,671 | 15,303 |
| Cumulative event rate, % (95% CI) | 0 | 1 (1-1) | 2 (1-2) | 2 (2-3) | 3 (3-3) | 4 (3-4) | 4 (4-4) | 5 (4-5) | 5 (5-6) | 6 (5-7) | 6 (6-7) |
| **All-cause mortality** |  |  |  |  |  |  |  |  |  |  |  |
| No. events | 0 | 777 | 1,270 | 1,652 | 1,953 | 2,202 | 2,383 | 2,507 | 2,606 | 2,659 | 2,688 |
| No. free of any events and death | 18,871 | 18,061 | 14,497 | 11,756 | 9,035 | 6,457 | 4,488 | 3,036 | 1,963 | 1,109 | 431 |
| No. censored | 0 | 33 | 3,104 | 5,463 | 7,883 | 10,212 | 12,000 | 13,328 | 14,302 | 15,103 | 15,752 |
| Cumulative event rate, % (95% CI) | 0 | 4 (4-4) | 7 (7-7) | 10 (9-10) | 12 (12-13) | 15 (14-16) | 18 (17-19) | 20 (20-21) | 24 (23-25) | 26 (25-27) | 29 (27-30) |
| IHD = ischaemic heart disease; MI = myocardial infarction | | | | | | | | | | | |
| Non-MI includes non-MI acute IHD and chronic IHD cases. | | | | | | | | | | | |

# Table S21: Cumulative event rate of recurrent MI events, stroke, heart failure and all-cause mortality following first adjudicated event of different IHD subtypes (combining before and after 28 days)

## (a) after first MI

|  | **Years since first MI (n = 2,481)** | | | | | | | | | | |
| --- | --- | --- | --- | --- | --- | --- | --- | --- | --- | --- | --- |
|  | **28 days** | **1** | **2** | **3** | **4** | **5** | **6** | **7** | **8** | **9** | **10** |
| **Recurrent MI event** |  |  |  |  |  |  |  |  |  |  |  |
| No. events | 209 | 341 | 386 | 420 | 441 | 472 | 490 | 502 | 507 | 508 | 513 |
| No. free of any events and death | 2,158 | 1,922 | 1,555 | 1,261 | 970 | 681 | 474 | 317 | 210 | 115 | 45 |
| No. deaths | 113 | 214 | 276 | 313 | 353 | 381 | 403 | 414 | 425 | 429 | 431 |
| No. censored | 1 | 4 | 264 | 487 | 717 | 947 | 1,114 | 1,248 | 1,339 | 1,429 | 1,492 |
| Cumulative event rate, % (95% CI) | 8 (7-10) | 14 (12-15) | 16 (14-17) | 17 (16-19) | 19 (17-20) | 21 (19-23) | 23 (21-25) | 25 (23-27) | 26 (23-28) | 26 (24-28) | 29 (26-33) |
| **Stroke** |  |  |  |  |  |  |  |  |  |  |  |
| No. events | 84 | 176 | 248 | 300 | 339 | 369 | 389 | 410 | 422 | 426 | 428 |
| No. free of any events and death | 2,165 | 1,931 | 1,534 | 1,210 | 919 | 635 | 440 | 288 | 188 | 102 | 40 |
| No. deaths | 232 | 371 | 441 | 486 | 521 | 556 | 579 | 593 | 604 | 608 | 612 |
| No. censored | 0 | 3 | 258 | 485 | 702 | 921 | 1,073 | 1,190 | 1,267 | 1,345 | 1,401 |
| Cumulative event rate, % (95% CI) | 3 (3-4) | 7 (6-8) | 10 (9-11) | 13 (12-14) | 15 (14-17) | 18 (16-19) | 20 (18-22) | 23 (20-25) | 25 (22-27) | 26 (23-29) | 27 (24-30) |
| **Heart failure** |  |  |  |  |  |  |  |  |  |  |  |
| No. events | 37 | 63 | 79 | 96 | 110 | 116 | 122 | 130 | 136 | 138 | 139 |
| No. free of any events and death | 2,196 | 2,020 | 1,659 | 1,342 | 1,034 | 737 | 526 | 357 | 237 | 137 | 54 |
| No. deaths | 247 | 394 | 474 | 525 | 572 | 612 | 637 | 658 | 672 | 677 | 682 |
| No. censored | 1 | 4 | 269 | 518 | 765 | 1,016 | 1,196 | 1,336 | 1,436 | 1,529 | 1,606 |
| Cumulative event rate, % (95% CI) | 1 (1-2) | 3 (2-3) | 3 (3-4) | 4 (3-5) | 5 (4-6) | 5 (4-6) | 6 (5-7) | 7 (6-9) | 8 (7-10) | 9 (7-11) | 10 (8-12) |
| **All-cause mortality** |  |  |  |  |  |  |  |  |  |  |  |
| No. events | 260 | 424 | 513 | 575 | 629 | 677 | 710 | 735 | 755 | 761 | 766 |
| No. free of any events and death | 2,220 | 2,053 | 1,687 | 1,373 | 1,066 | 755 | 538 | 371 | 248 | 140 | 57 |
| No. censored | 1 | 4 | 281 | 533 | 786 | 1,049 | 1,233 | 1,375 | 1,478 | 1,580 | 1,658 |
| Cumulative event rate, % (95% CI) | 10 (9-12) | 17 (16-19) | 21 (19-23) | 24 (22-26) | 27 (26-29) | 31 (29-33) | 34 (32-37) | 38 (35-40) | 42 (39-45) | 44 (41-47) | 46 (43-50) |
| IHD = ischaemic heart disease; MI = myocardial infarction | | | | | | | | | | | |
| Non-MI includes non-MI acute IHD and chronic IHD cases. | | | | | | | | | | | |

## (b) after first non-MI

|  | **Years since first non-MI (n = 16,778)** | | | | | | | | | | |
| --- | --- | --- | --- | --- | --- | --- | --- | --- | --- | --- | --- |
|  | **28 days** | **1** | **2** | **3** | **4** | **5** | **6** | **7** | **8** | **9** | **10** |
| **Recurrent MI event** |  |  |  |  |  |  |  |  |  |  |  |
| No. events | 92 | 316 | 463 | 570 | 643 | 703 | 749 | 782 | 804 | 823 | 829 |
| No. free of any events and death | 16,591 | 15,844 | 12,610 | 10,181 | 7,768 | 5,543 | 3,817 | 2,563 | 1,644 | 916 | 353 |
| No. deaths | 94 | 587 | 910 | 1,155 | 1,359 | 1,511 | 1,624 | 1,704 | 1,764 | 1,795 | 1,813 |
| No. censored | 1 | 31 | 2,795 | 4,872 | 7,008 | 9,021 | 10,588 | 11,729 | 12,566 | 13,244 | 13,783 |
| Cumulative event rate, % (95% CI) | 1 (0-1) | 2 (2-2) | 3 (3-3) | 4 (3-4) | 4 (4-5) | 5 (5-6) | 6 (6-6) | 7 (6-7) | 8 (7-8) | 9 (8-9) | 9 (8-10) |
| **Stroke** |  |  |  |  |  |  |  |  |  |  |  |
| No. events | 642 | 1,435 | 2,109 | 2,633 | 2,994 | 3,256 | 3,426 | 3,512 | 3,599 | 3,640 | 3,665 |
| No. free of any events and death | 16,032 | 14,735 | 11,283 | 8,743 | 6,401 | 4,389 | 2,946 | 1,972 | 1,212 | 674 | 259 |
| No. deaths | 103 | 578 | 848 | 1,043 | 1,189 | 1,302 | 1,383 | 1,423 | 1,462 | 1,490 | 1,504 |
| No. censored | 1 | 30 | 2,538 | 4,359 | 6,194 | 7,831 | 9,023 | 9,871 | 10,505 | 10,974 | 11,350 |
| Cumulative event rate, % (95% CI) | 4 (4-4) | 9 (8-9) | 13 (12-13) | 17 (16-18) | 21 (20-21) | 24 (23-25) | 27 (26-28) | 29 (28-30) | 32 (31-33) | 34 (33-36) | 37 (36-39) |
| **Heart failure** |  |  |  |  |  |  |  |  |  |  |  |
| No. events | 161 | 290 | 408 | 489 | 552 | 602 | 628 | 654 | 668 | 676 | 677 |
| No. free of any events and death | 16,502 | 15,780 | 12,596 | 10,174 | 7,793 | 5,551 | 3,846 | 2,591 | 1,667 | 942 | 367 |
| No. deaths | 114 | 678 | 1,032 | 1,311 | 1,520 | 1,695 | 1,820 | 1,907 | 1,977 | 2,016 | 2,035 |
| No. censored | 1 | 30 | 2,742 | 4,804 | 6,913 | 8,930 | 10,484 | 11,626 | 12,466 | 13,144 | 13,699 |
| Cumulative event rate, % (95% CI) | 1 (1-1) | 2 (2-2) | 2 (2-3) | 3 (3-3) | 4 (3-4) | 4 (4-5) | 5 (4-5) | 5 (5-6) | 6 (5-7) | 6 (6-7) | 7 (6-7) |
| **All-cause mortality** |  |  |  |  |  |  |  |  |  |  |  |
| No. events | 126 | 739 | 1,143 | 1,463 | 1,710 | 1,911 | 2,059 | 2,158 | 2,237 | 2,284 | 2,308 |
| No. free of any events and death | 16,651 | 16,008 | 12,810 | 10,383 | 7,969 | 5,702 | 3,950 | 2,665 | 1,715 | 969 | 374 |
| No. censored | 1 | 31 | 2,825 | 4,932 | 7,099 | 9,165 | 10,769 | 11,955 | 12,826 | 13,525 | 14,096 |
| Cumulative event rate, % (95% CI) | 1 (1-1) | 4 (4-5) | 7 (7-7) | 10 (9-10) | 12 (11-13) | 15 (14-15) | 17 (16-18) | 20 (19-20) | 22 (21-24) | 25 (24-26) | 28 (26-29) |
| IHD = ischaemic heart disease; MI = myocardial infarction | | | | | | | | | | | |
| Non-MI includes non-MI acute IHD and chronic IHD cases. | | | | | | | | | | | |

## (c) after first IHD

|  | **Years since first IHD (n = 19,259)** | | | | | | | | | | |
| --- | --- | --- | --- | --- | --- | --- | --- | --- | --- | --- | --- |
|  | **28 days** | **1** | **2** | **3** | **4** | **5** | **6** | **7** | **8** | **9** | **10** |
| **Recurrent MI event** |  |  |  |  |  |  |  |  |  |  |  |
| No. events | 301 | 657 | 849 | 990 | 1,084 | 1,175 | 1,239 | 1,284 | 1,311 | 1,331 | 1,342 |
| No. free of any events and death | 18,749 | 17,766 | 14,165 | 11,442 | 8,738 | 6,224 | 4,291 | 2,880 | 1,854 | 1,031 | 398 |
| No. deaths | 207 | 801 | 1,186 | 1,468 | 1,712 | 1,892 | 2,027 | 2,118 | 2,189 | 2,224 | 2,244 |
| No. censored | 2 | 35 | 3,059 | 5,359 | 7,725 | 9,968 | 11,702 | 12,977 | 13,905 | 14,673 | 15,275 |
| Cumulative event rate, % (95% CI) | 2 (1-2) | 3 (3-4) | 4 (4-5) | 5 (5-6) | 6 (6-7) | 7 (7-8) | 8 (8-9) | 9 (9-10) | 10 (9-11) | 11 (10-12) | 12 (11-13) |
| **Stroke** |  |  |  |  |  |  |  |  |  |  |  |
| No. events | 726 | 1,611 | 2,357 | 2,933 | 3,333 | 3,625 | 3,815 | 3,922 | 4,021 | 4,066 | 4,093 |
| No. free of any events and death | 18,197 | 16,666 | 12,817 | 9,953 | 7,320 | 5,024 | 3,386 | 2,260 | 1,400 | 776 | 299 |
| No. deaths | 335 | 949 | 1,289 | 1,529 | 1,710 | 1,858 | 1,962 | 2,016 | 2,066 | 2,098 | 2,116 |
| No. censored | 1 | 33 | 2,796 | 4,844 | 6,896 | 8,752 | 10,096 | 11,061 | 11,772 | 12,319 | 12,751 |
| Cumulative event rate, % (95% CI) | 4 (4-4) | 8 (8-9) | 13 (12-13) | 17 (16-17) | 20 (19-21) | 23 (22-24) | 26 (25-27) | 28 (27-29) | 31 (30-32) | 33 (32-34) | 36 (34-37) |
| **Heart failure** |  |  |  |  |  |  |  |  |  |  |  |
| No. events | 198 | 353 | 487 | 585 | 662 | 718 | 750 | 784 | 804 | 814 | 816 |
| No. free of any events and death | 18,698 | 17,800 | 14,255 | 11,516 | 8,827 | 6,288 | 4,372 | 2,948 | 1,904 | 1,079 | 421 |
| No. deaths | 361 | 1,072 | 1,506 | 1,836 | 2,092 | 2,307 | 2,457 | 2,565 | 2,649 | 2,693 | 2,717 |
| No. censored | 2 | 34 | 3,011 | 5,322 | 7,678 | 9,946 | 11,680 | 12,962 | 13,902 | 14,673 | 15,305 |
| Cumulative event rate, % (95% CI) | 1 (1-1) | 2 (2-2) | 3 (2-3) | 3 (3-4) | 4 (4-4) | 5 (4-5) | 5 (5-5) | 6 (5-6) | 6 (6-7) | 7 (6-7) | 7 (6-8) |
| **All-cause mortality** |  |  |  |  |  |  |  |  |  |  |  |
| No. events | 386 | 1,163 | 1,656 | 2,038 | 2,339 | 2,588 | 2,769 | 2,893 | 2,992 | 3,045 | 3,074 |
| No. free of any events and death | 18,871 | 18,061 | 14,497 | 11,756 | 9,035 | 6,457 | 4,488 | 3,036 | 1,963 | 1,109 | 431 |
| No. censored | 2 | 35 | 3,106 | 5,465 | 7,885 | 10,214 | 12,002 | 13,330 | 14,304 | 15,105 | 15,754 |
| Cumulative event rate, % (95% CI) | 2 (2-2) | 6 (6-6) | 9 (8-9) | 11 (11-12) | 14 (13-15) | 17 (16-17) | 19 (19-20) | 22 (21-23) | 25 (24-26) | 28 (26-29) | 30 (29-32) |
| IHD = ischaemic heart disease; MI = myocardial infarction | | | | | | | | | | | |
| Non-MI includes non-MI acute IHD and chronic IHD cases. | | | | | | | | | | | |

# Table S22: Cumulative event rates of recurrent MI events, stroke, heart failure and all-cause mortality from 28 days following first reported event of different IHD subtypes

## (a) after first MI

|  | **Years since first MI (n = 5,410)** | | | | | | | | | | |
| --- | --- | --- | --- | --- | --- | --- | --- | --- | --- | --- | --- |
|  | **28 days** | **1** | **2** | **3** | **4** | **5** | **6** | **7** | **8** | **9** | **10** |
| **Recurrent MI event** |  |  |  |  |  |  |  |  |  |  |  |
| No. events | 0 | 186 | 248 | 296 | 320 | 343 | 361 | 370 | 375 | 382 | 388 |
| No. free of any events and death | 2,253 | 1,771 | 1,465 | 1,218 | 992 | 758 | 578 | 441 | 326 | 192 | 84 |
| No. deaths | 0 | 81 | 124 | 165 | 186 | 211 | 229 | 244 | 257 | 269 | 270 |
| No. censored | 0 | 215 | 416 | 574 | 755 | 941 | 1,085 | 1,198 | 1,295 | 1,410 | 1,511 |
| Cumulative event rate, % (95% CI) | 0 | 9 (7-10) | 12 (10-13) | 15 (13-16) | 16 (15-18) | 18 (16-20) | 20 (18-22) | 21 (19-23) | 22 (20-24) | 23 (21-26) | 26 (23-29) |
| **Stroke** |  |  |  |  |  |  |  |  |  |  |  |
| No. events | 0 | 99 | 176 | 233 | 288 | 319 | 345 | 361 | 380 | 389 | 396 |
| No. free of any events and death | 2,306 | 1,846 | 1,502 | 1,226 | 982 | 739 | 566 | 423 | 297 | 172 | 71 |
| No. deaths | 0 | 147 | 207 | 254 | 279 | 308 | 328 | 344 | 355 | 369 | 371 |
| No. censored | 0 | 214 | 421 | 593 | 757 | 940 | 1,067 | 1,178 | 1,274 | 1,376 | 1,468 |
| Cumulative event rate, % (95% CI) | 0 | 4 (4-5) | 8 (7-10) | 12 (10-13) | 15 (14-17) | 18 (16-20) | 20 (18-22) | 22 (20-24) | 25 (23-28) | 27 (24-30) | 30 (27-33) |
| **Heart failure** |  |  |  |  |  |  |  |  |  |  |  |
| No. events | 0 | 32 | 49 | 61 | 71 | 80 | 85 | 88 | 95 | 99 | 102 |
| No. free of any events and death | 2,351 | 1,935 | 1,638 | 1,378 | 1,142 | 882 | 698 | 537 | 391 | 239 | 103 |
| No. deaths | 0 | 158 | 222 | 288 | 316 | 350 | 375 | 398 | 418 | 434 | 438 |
| No. censored | 0 | 226 | 442 | 624 | 822 | 1,039 | 1,193 | 1,328 | 1,447 | 1,579 | 1,708 |
| Cumulative event rate, % (95% CI) | 0 | 1 (1-2) | 2 (2-3) | 3 (2-4) | 4 (3-5) | 4 (3-5) | 5 (4-6) | 5 (4-6) | 6 (5-8) | 7 (6-9) | 8 (6-10) |
| **All-cause mortality** |  |  |  |  |  |  |  |  |  |  |  |
| No. events | 0 | 176 | 250 | 320 | 357 | 399 | 429 | 454 | 477 | 495 | 499 |
| No. free of any events and death | 2,387 | 1,979 | 1,675 | 1,420 | 1,176 | 912 | 722 | 559 | 415 | 253 | 112 |
| No. censored | 0 | 232 | 462 | 647 | 854 | 1,076 | 1,236 | 1,374 | 1,495 | 1,639 | 1,776 |
| Cumulative event rate, % (95% CI) | 0 | 8 (7-9) | 11 (10-13) | 15 (14-17) | 18 (16-19) | 21 (19-23) | 24 (22-26) | 27 (24-29) | 30 (27-33) | 34 (31-37) | 35 (32-38) |
| IHD = ischaemic heart disease; MI = myocardial infarction | | | | | | | | | | | |
| Non-MI includes non-MI acute IHD and chronic IHD cases. | | | | | | | | | | | |

## (b) after first non-MI

|  | **Years since first non-MI (n = 42,282)** | | | | | | | | | | |
| --- | --- | --- | --- | --- | --- | --- | --- | --- | --- | --- | --- |
|  | **28 days** | **1** | **2** | **3** | **4** | **5** | **6** | **7** | **8** | **9** | **10** |
| **Recurrent MI event** |  |  |  |  |  |  |  |  |  |  |  |
| No. events | 0 | 384 | 667 | 885 | 1,038 | 1,173 | 1,277 | 1,355 | 1,406 | 1,452 | 1,471 |
| No. free of any events and death | 39,791 | 34,336 | 28,185 | 23,750 | 19,346 | 15,585 | 12,040 | 8,725 | 6,511 | 4,265 | 2,168 |
| No. deaths | 0 | 1,129 | 1,873 | 2,458 | 2,982 | 3,415 | 3,771 | 3,999 | 4,195 | 4,344 | 4,409 |
| No. censored | 0 | 3,942 | 9,066 | 12,698 | 16,425 | 19,618 | 22,703 | 25,712 | 27,679 | 29,730 | 31,743 |
| Cumulative event rate, % (95% CI) | 0 | 1 (1-1) | 2 (2-2) | 3 (2-3) | 3 (3-3) | 4 (4-4) | 5 (4-5) | 5 (5-5) | 6 (5-6) | 6 (6-7) | 7 (6-7) |
| **Stroke** |  |  |  |  |  |  |  |  |  |  |  |
| No. events | 0 | 1,673 | 3,025 | 4,060 | 4,784 | 5,372 | 5,814 | 6,103 | 6,325 | 6,460 | 6,551 |
| No. free of any events and death | 38,367 | 32,021 | 25,446 | 20,811 | 16,514 | 12,992 | 9,860 | 6,996 | 5,167 | 3,314 | 1,634 |
| No. deaths | 0 | 1,039 | 1,679 | 2,153 | 2,540 | 2,859 | 3,105 | 3,246 | 3,365 | 3,467 | 3,510 |
| No. censored | 0 | 3,634 | 8,217 | 11,343 | 14,529 | 17,144 | 19,588 | 22,022 | 23,510 | 25,126 | 26,672 |
| Cumulative event rate, % (95% CI) | 0 | 5 (4-5) | 9 (8-9) | 13 (12-13) | 16 (15-16) | 19 (18-19) | 21 (21-22) | 24 (23-24) | 26 (25-26) | 28 (27-28) | 30 (29-31) |
| **Heart failure** |  |  |  |  |  |  |  |  |  |  |  |
| No. events | 0 | 436 | 662 | 859 | 1,014 | 1,121 | 1,207 | 1,272 | 1,337 | 1,372 | 1,399 |
| No. free of any events and death | 39,491 | 33,944 | 27,925 | 23,500 | 19,165 | 15,463 | 11,950 | 8,655 | 6,434 | 4,221 | 2,138 |
| No. deaths | 0 | 1,185 | 2,003 | 2,638 | 3,179 | 3,637 | 4,013 | 4,257 | 4,457 | 4,603 | 4,663 |
| No. censored | 0 | 3,926 | 8,901 | 12,494 | 16,133 | 19,270 | 22,321 | 25,307 | 27,263 | 29,295 | 31,291 |
| Cumulative event rate, % (95% CI) | 0 | 1 (1-1) | 2 (2-2) | 3 (2-3) | 3 (3-3) | 4 (3-4) | 4 (4-4) | 5 (4-5) | 5 (5-6) | 6 (6-6) | 6 (6-7) |
| **All-cause mortality** |  |  |  |  |  |  |  |  |  |  |  |
| No. events | 0 | 1,330 | 2,251 | 2,980 | 3,610 | 4,149 | 4,588 | 4,875 | 5,113 | 5,296 | 5,382 |
| No. free of any events and death | 39,948 | 34,645 | 28,546 | 24,128 | 19,718 | 15,914 | 12,331 | 8,960 | 6,693 | 4,396 | 2,224 |
| No. censored | 0 | 3,973 | 9,151 | 12,840 | 16,620 | 19,885 | 23,029 | 26,113 | 28,142 | 30,256 | 32,342 |
| Cumulative event rate, % (95% CI) | 0 | 3 (3-4) | 6 (6-6) | 9 (8-9) | 11 (11-12) | 14 (14-14) | 17 (16-17) | 19 (18-19) | 21 (21-22) | 24 (23-25) | 26 (25-27) |
| IHD = ischaemic heart disease; MI = myocardial infarction | | | | | | | | | | | |
| Non-MI includes non-MI acute IHD and chronic IHD cases. | | | | | | | | | | | |

## (c) after first IHD

|  | **Years since first IHD (n = 47,692)** | | | | | | | | | | |
| --- | --- | --- | --- | --- | --- | --- | --- | --- | --- | --- | --- |
|  | **28 days** | **1** | **2** | **3** | **4** | **5** | **6** | **7** | **8** | **9** | **10** |
| **Recurrent MI event** |  |  |  |  |  |  |  |  |  |  |  |
| No. events | 0 | 570 | 915 | 1,181 | 1,358 | 1,516 | 1,638 | 1,725 | 1,781 | 1,834 | 1,859 |
| No. free of any events and death | 42,044 | 36,107 | 29,650 | 24,968 | 20,338 | 16,343 | 12,618 | 9,166 | 6,837 | 4,457 | 2,252 |
| No. deaths | 0 | 1,210 | 1,997 | 2,623 | 3,168 | 3,626 | 4,000 | 4,243 | 4,452 | 4,613 | 4,679 |
| No. censored | 0 | 4,157 | 9,482 | 13,272 | 17,180 | 20,559 | 23,788 | 26,910 | 28,974 | 31,140 | 33,254 |
| Cumulative event rate, % (95% CI) | 0 | 1 (1-2) | 2 (2-3) | 3 (3-3) | 4 (4-4) | 5 (4-5) | 5 (5-6) | 6 (6-6) | 7 (6-7) | 7 (7-8) | 8 (7-8) |
| **Stroke** |  |  |  |  |  |  |  |  |  |  |  |
| No. events | 0 | 1,772 | 3,201 | 4,293 | 5,072 | 5,691 | 6,159 | 6,464 | 6,705 | 6,849 | 6,947 |
| No. free of any events and death | 40,673 | 33,867 | 26,948 | 22,037 | 17,496 | 13,731 | 10,426 | 7,419 | 5,464 | 3,486 | 1,705 |
| No. deaths | 0 | 1,186 | 1,886 | 2,407 | 2,819 | 3,167 | 3,433 | 3,590 | 3,720 | 3,836 | 3,881 |
| No. censored | 0 | 3,848 | 8,638 | 11,936 | 15,286 | 18,084 | 20,655 | 23,200 | 24,784 | 26,502 | 28,140 |
| Cumulative event rate, % (95% CI) | 0 | 5 (4-5) | 9 (8-9) | 12 (12-13) | 16 (15-16) | 18 (18-19) | 21 (21-22) | 23 (23-24) | 26 (25-26) | 28 (27-28) | 30 (29-30) |
| **Heart failure** |  |  |  |  |  |  |  |  |  |  |  |
| No. events | 0 | 468 | 711 | 920 | 1,085 | 1,201 | 1,292 | 1,360 | 1,432 | 1,471 | 1,501 |
| No. free of any events and death | 41,842 | 35,879 | 29,563 | 24,878 | 20,307 | 16,345 | 12,648 | 9,192 | 6,825 | 4,460 | 2,241 |
| No. deaths | 0 | 1,343 | 2,225 | 2,926 | 3,495 | 3,987 | 4,388 | 4,655 | 4,875 | 5,037 | 5,101 |
| No. censored | 0 | 4,152 | 9,343 | 13,118 | 16,955 | 20,309 | 23,514 | 26,635 | 28,710 | 30,874 | 32,999 |
| Cumulative event rate, % (95% CI) | 0 | 1 (1-1) | 2 (2-2) | 3 (2-3) | 3 (3-3) | 4 (4-4) | 4 (4-5) | 5 (5-5) | 5 (5-6) | 6 (6-6) | 7 (6-7) |
| **All-cause mortality** |  |  |  |  |  |  |  |  |  |  |  |
| No. events | 0 | 1,506 | 2,501 | 3,300 | 3,967 | 4,548 | 5,017 | 5,329 | 5,590 | 5,791 | 5,881 |
| No. free of any events and death | 42,335 | 36,624 | 30,221 | 25,548 | 20,894 | 16,826 | 13,053 | 9,519 | 7,108 | 4,649 | 2,336 |
| No. censored | 0 | 4,205 | 9,613 | 13,487 | 17,474 | 20,961 | 24,265 | 27,487 | 29,637 | 31,895 | 34,118 |
| Cumulative event rate, % (95% CI) | 0 | 4 (4-4) | 7 (6-7) | 9 (9-9) | 12 (11-12) | 14 (14-15) | 17 (17-18) | 19 (19-20) | 22 (21-22) | 24 (24-25) | 26 (26-27) |
| IHD = ischaemic heart disease; MI = myocardial infarction | | | | | | | | | | | |
| Non-MI includes non-MI acute IHD and chronic IHD cases. | | | | | | | | | | | |

# Figure S1: Associations of SBP and BMI with risk of MI by adjudication status


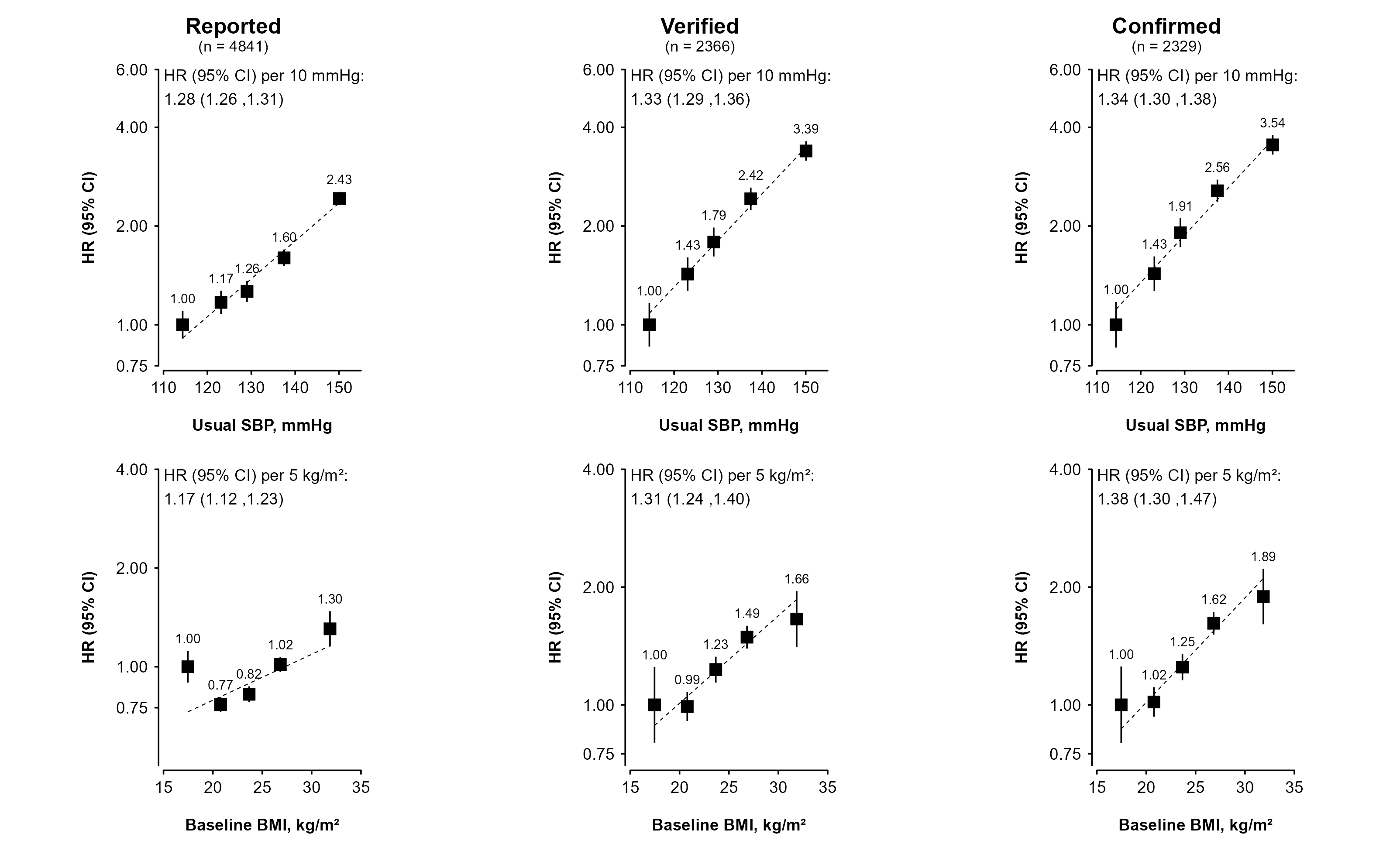


SBP = systolic blood pressure; BMI = body mass index; MI = myocardial infarction

# Figure S2: Associations of SBP and BMI with risk of non-MI by adjudication status


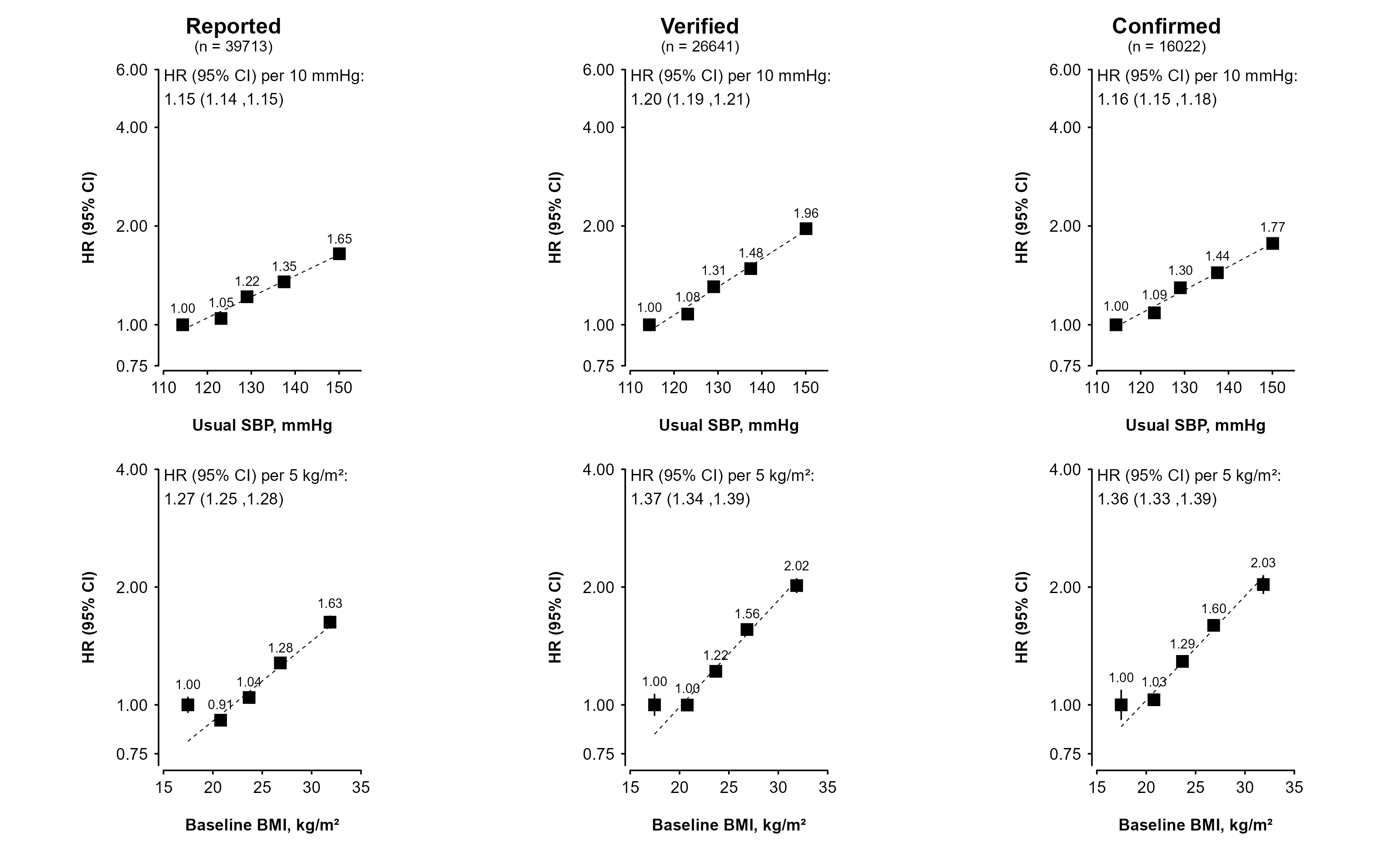


SBP = systolic blood pressure; BMI = body mass index; MI = myocardial infarction

Non-MI includes non-MI acute IHD and chronic IHD cases

# Figure S3: Age-specific 28-day case-fatality rates after first adjudicated event of different IHD types by sex and area


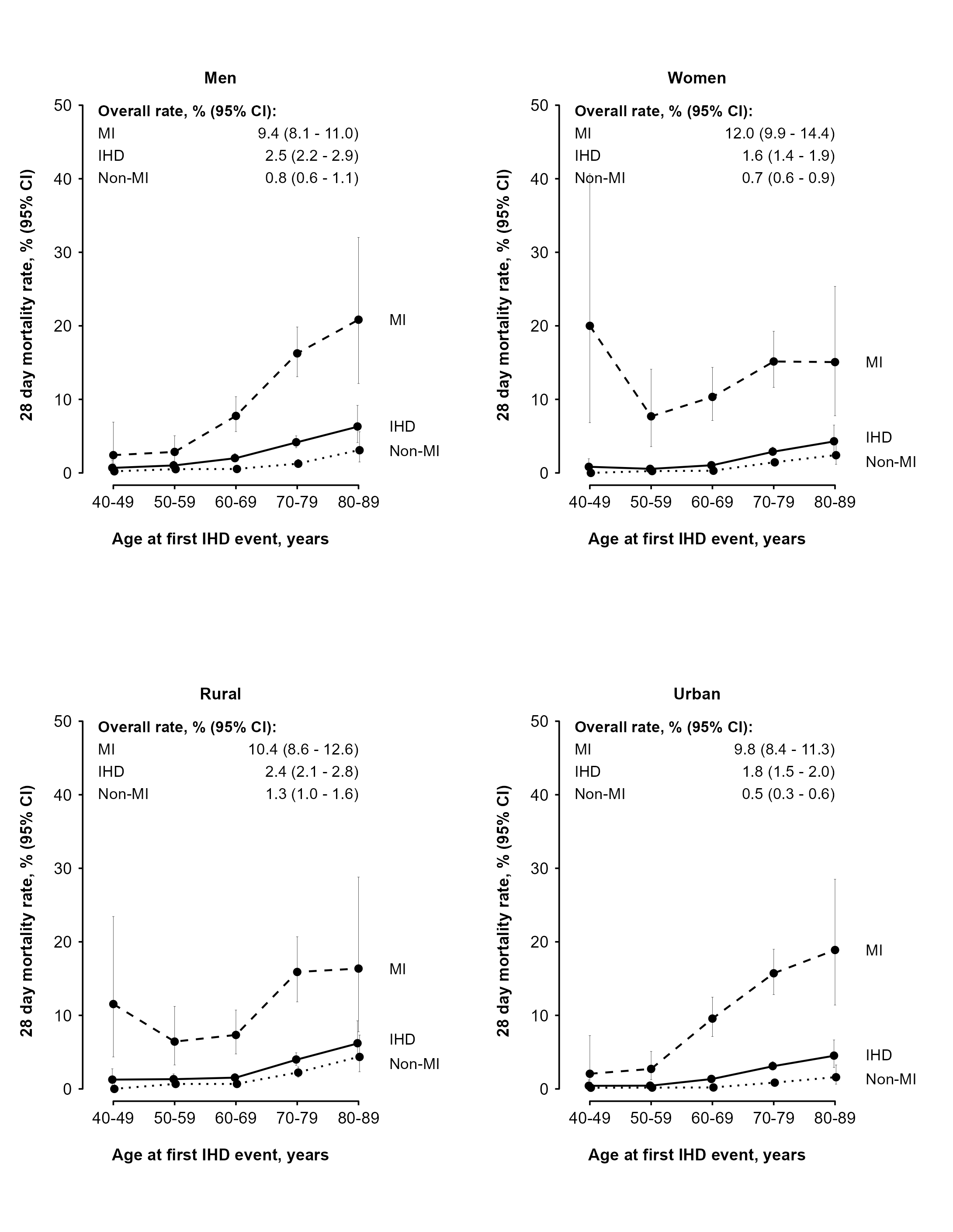


IHD = ischaemic heart disease; MI = myocardial infarction

Non-MI includes non-MI acute IHD and chronic IHD cases

# Figure S4: Age-specific 28-day case-fatality rates after first adjudicated event of different IHD types by hospital tier


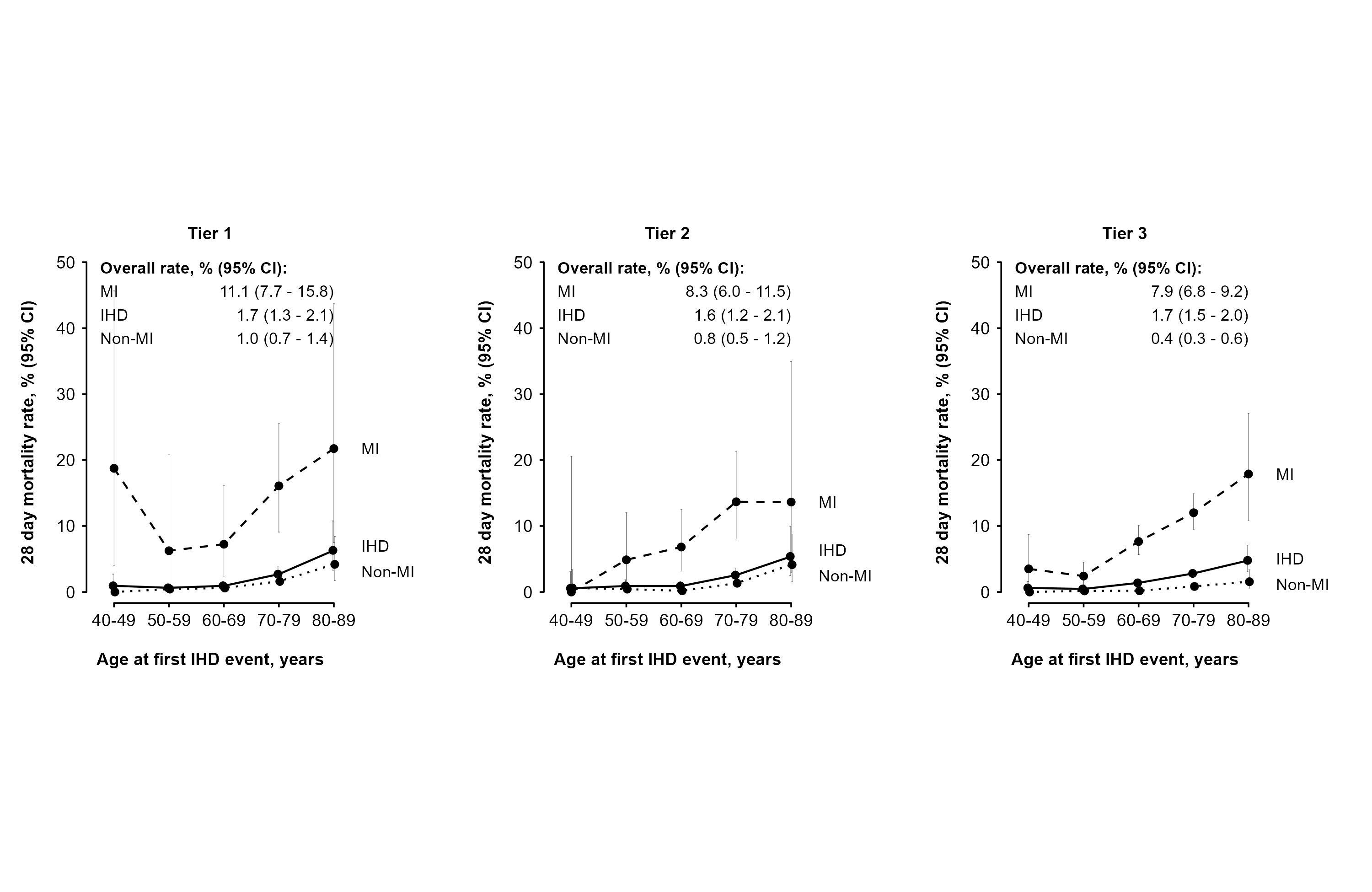


IHD = ischaemic heart disease; MI = myocardial infarction

Non-MI includes non-MI acute IHD and chronic IHD cases

# Figure S5: Estimated cumulative event rate of recurrent MI events, stroke, heart failure and all-cause mortality from 28 days after first adjudicated event of different IHD types by sex


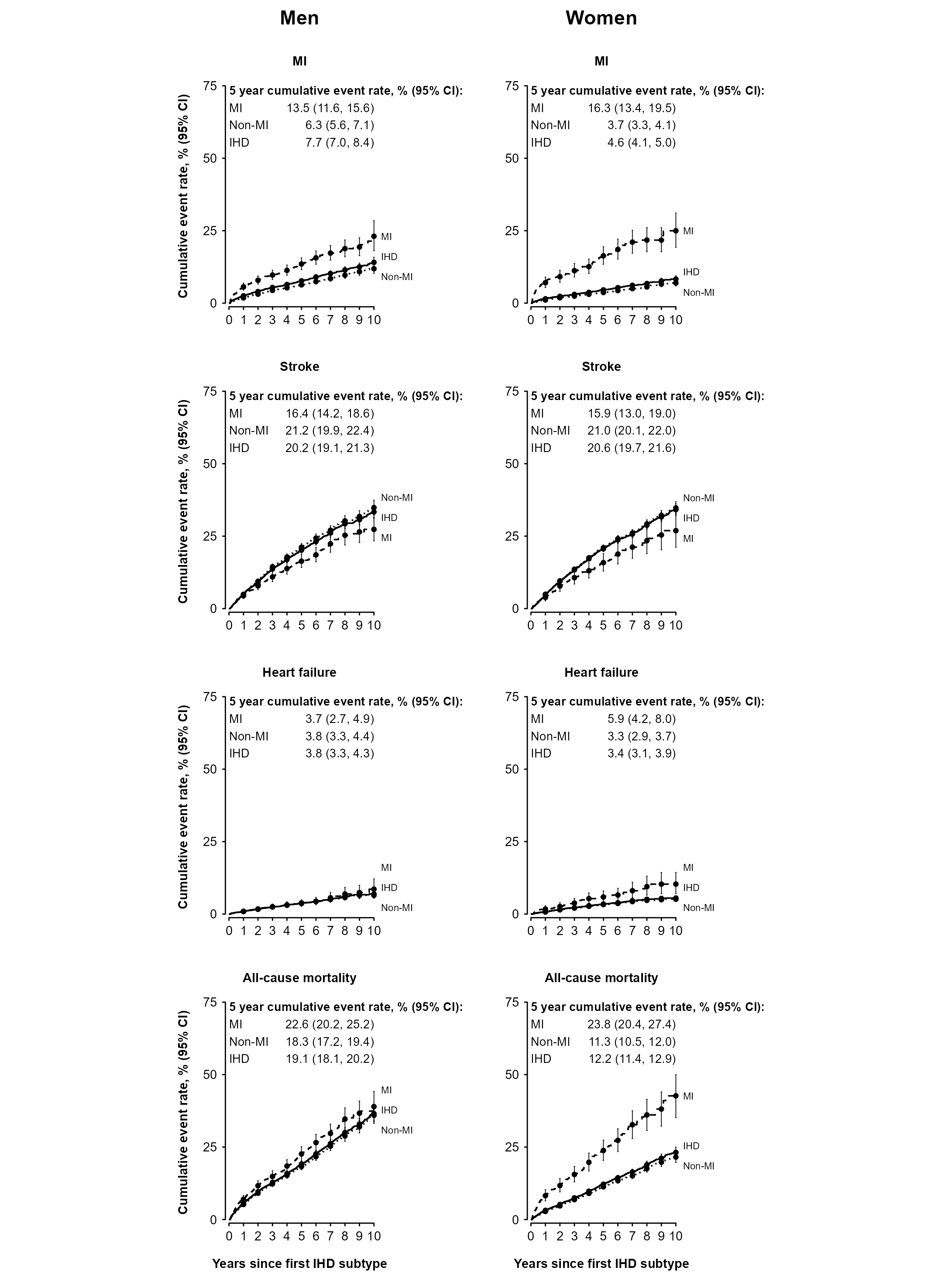


IHD = ischaemic heart disease; MI = myocardial infarction

Non-MI includes non-MI acute IHD and chronic IHD cases

# Figure S6: Estimated cumulative event rate of recurrent MI events, stroke, heart failure and all-cause mortality from 28 days after first adjudicated event of different IHD types by area


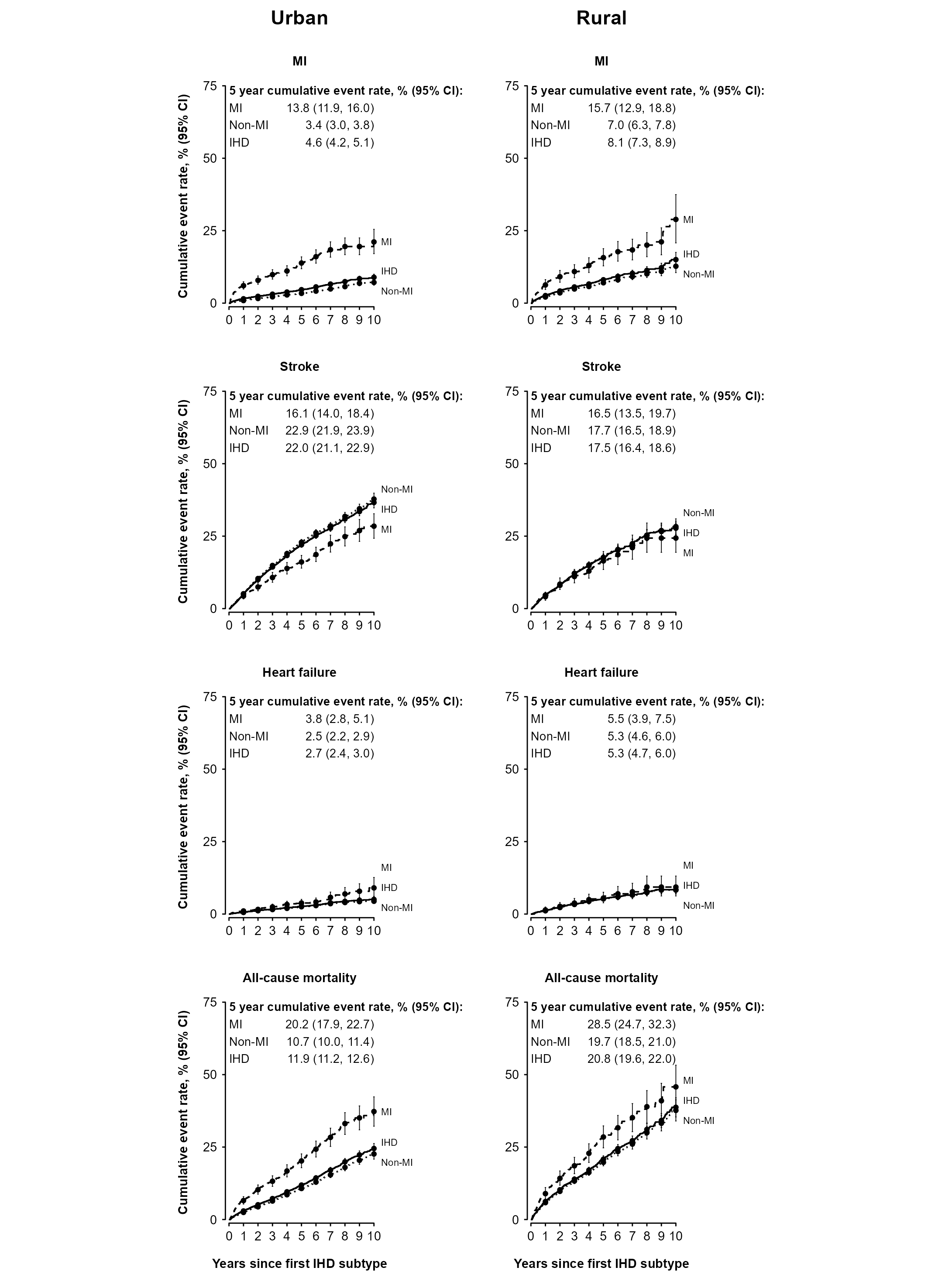


IHD = ischaemic heart disease; MI = myocardial infarction

Non-MI includes non-MI acute IHD and chronic IHD cases

# Figure S7: Estimated cumulative event rate of recurrent MI events, stroke, heart failure and all-cause mortality following first adjudicated event of different IHD types by hospital tier


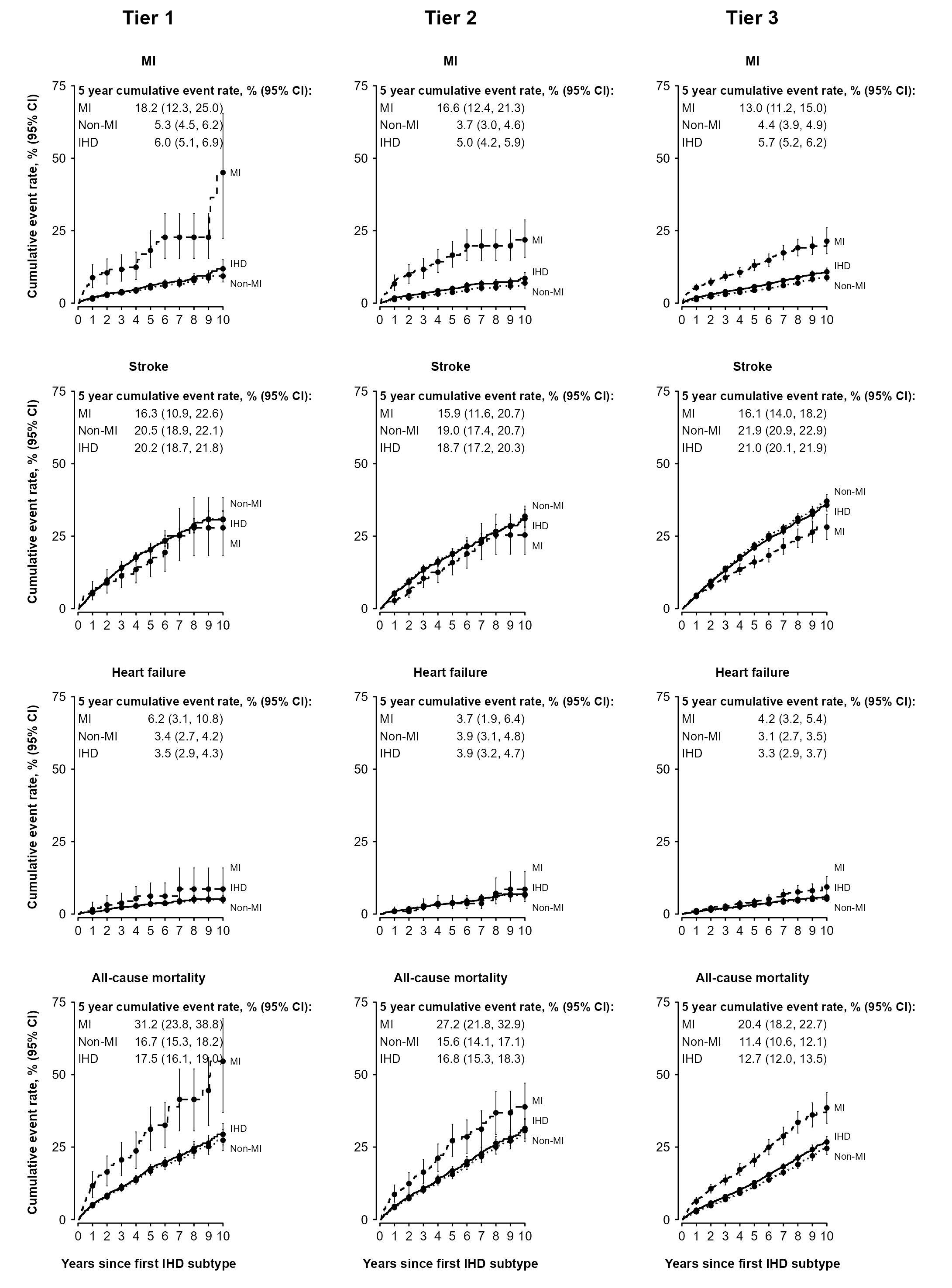


IHD = ischaemic heart disease; MI = myocardial infarction

Non-MI includes non-MI acute IHD and chronic IHD cases

# Figure S8: Estimated cumulative event rate of recurrent MI events, stroke, heart failure and all-cause mortality following first event of different IHD types by validation status


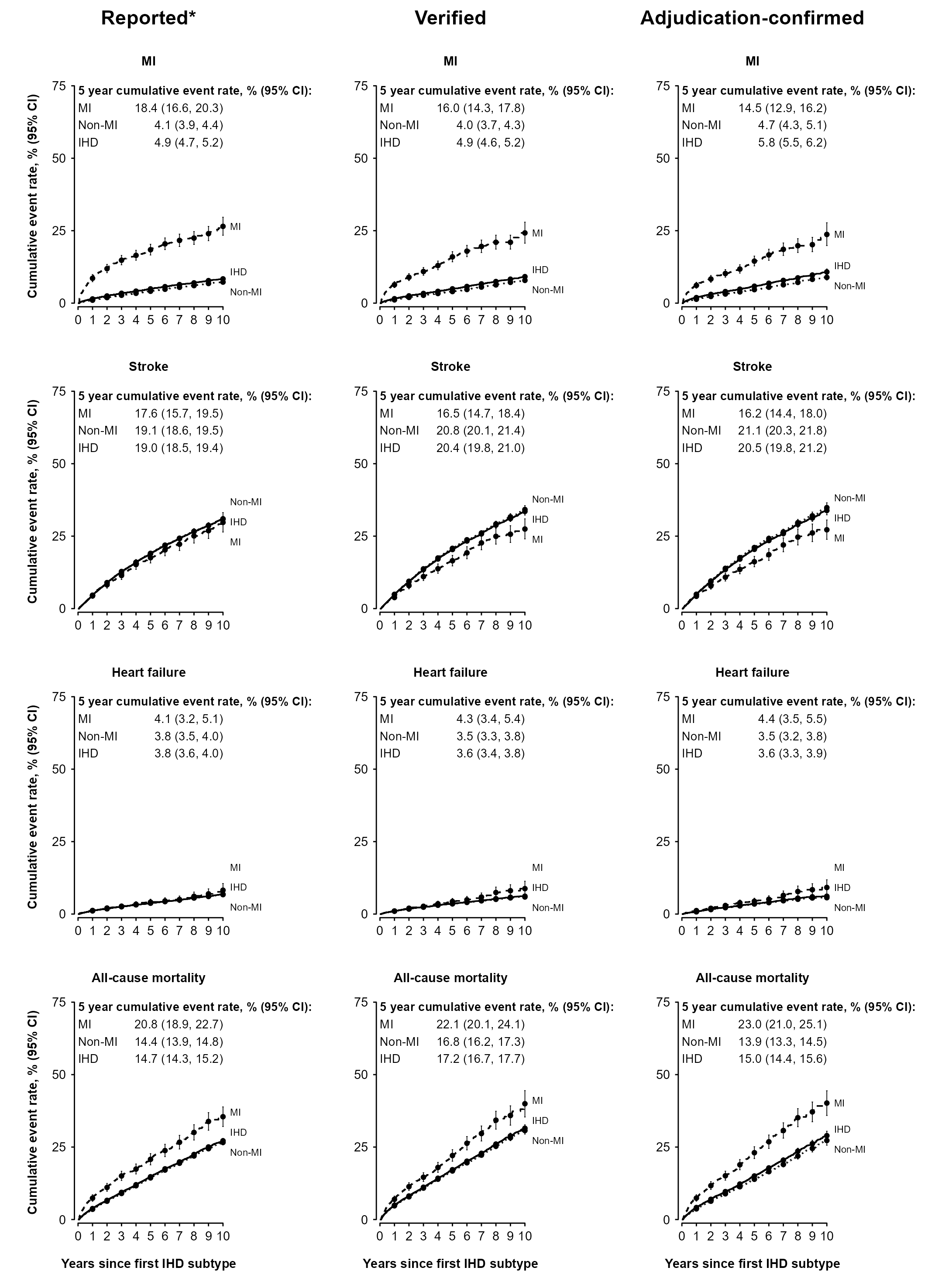


^*^ Reported IHD cases with accessible records

Excludes verified secondary IHD cases

IHD = ischaemic heart disease; MI = myocardial infarction

Non-MI includes non-MI acute IHD and chronic IHD cases

# Figure S9: Estimated cumulative event rate of recurrent MI events, stroke, heart failure and all-cause mortality from 28 days after first event of different IHD types


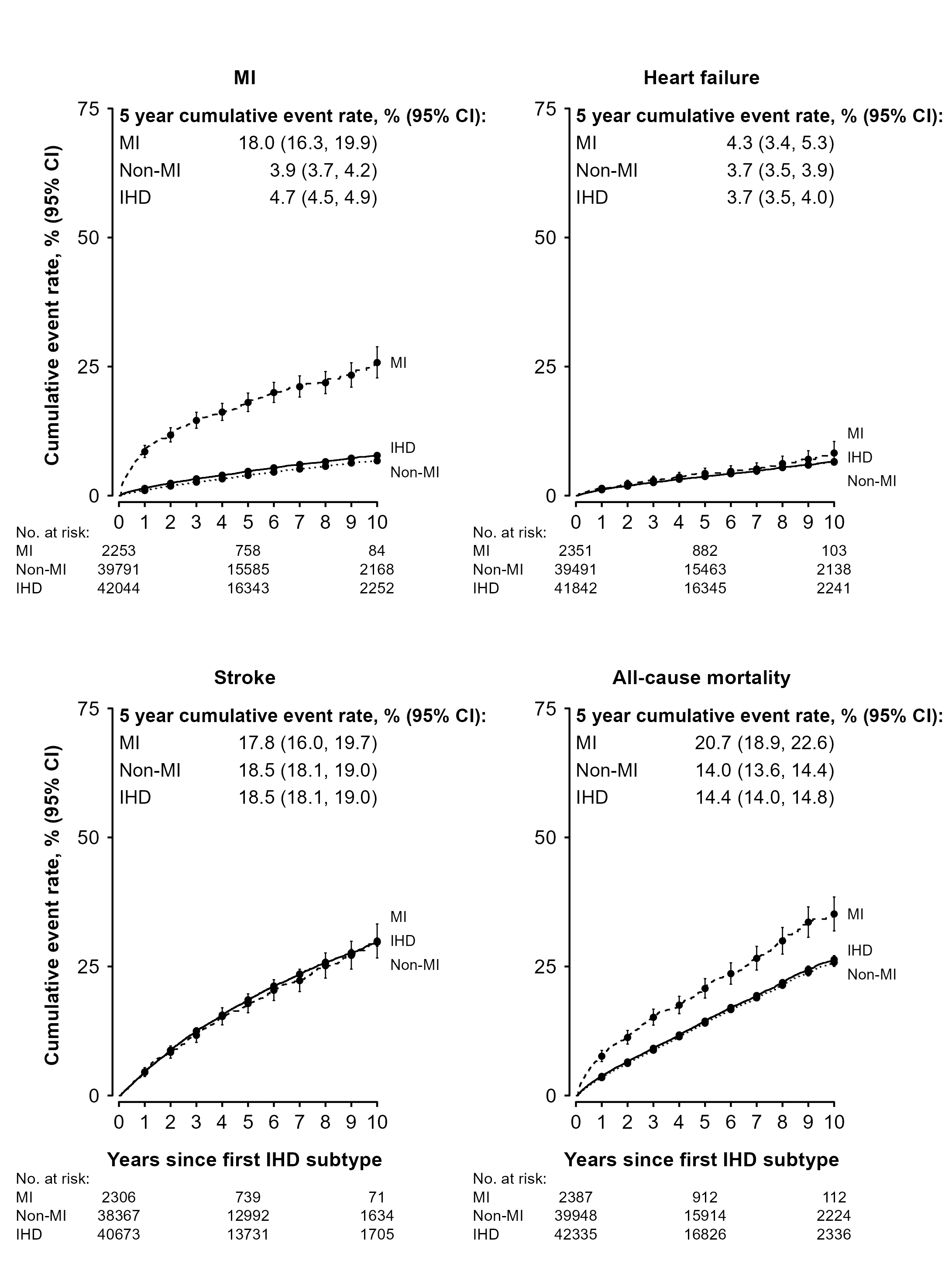


IHD = ischaemic heart disease; MI = myocardial infarction

Non-MI includes non-MI acute IHD and chronic IHD cases

# Figure S10: Estimated cumulative event rate of recurrent MI events, stroke, heart failure and all-cause mortality following first event of different IHD types (combining before and after 28 days)


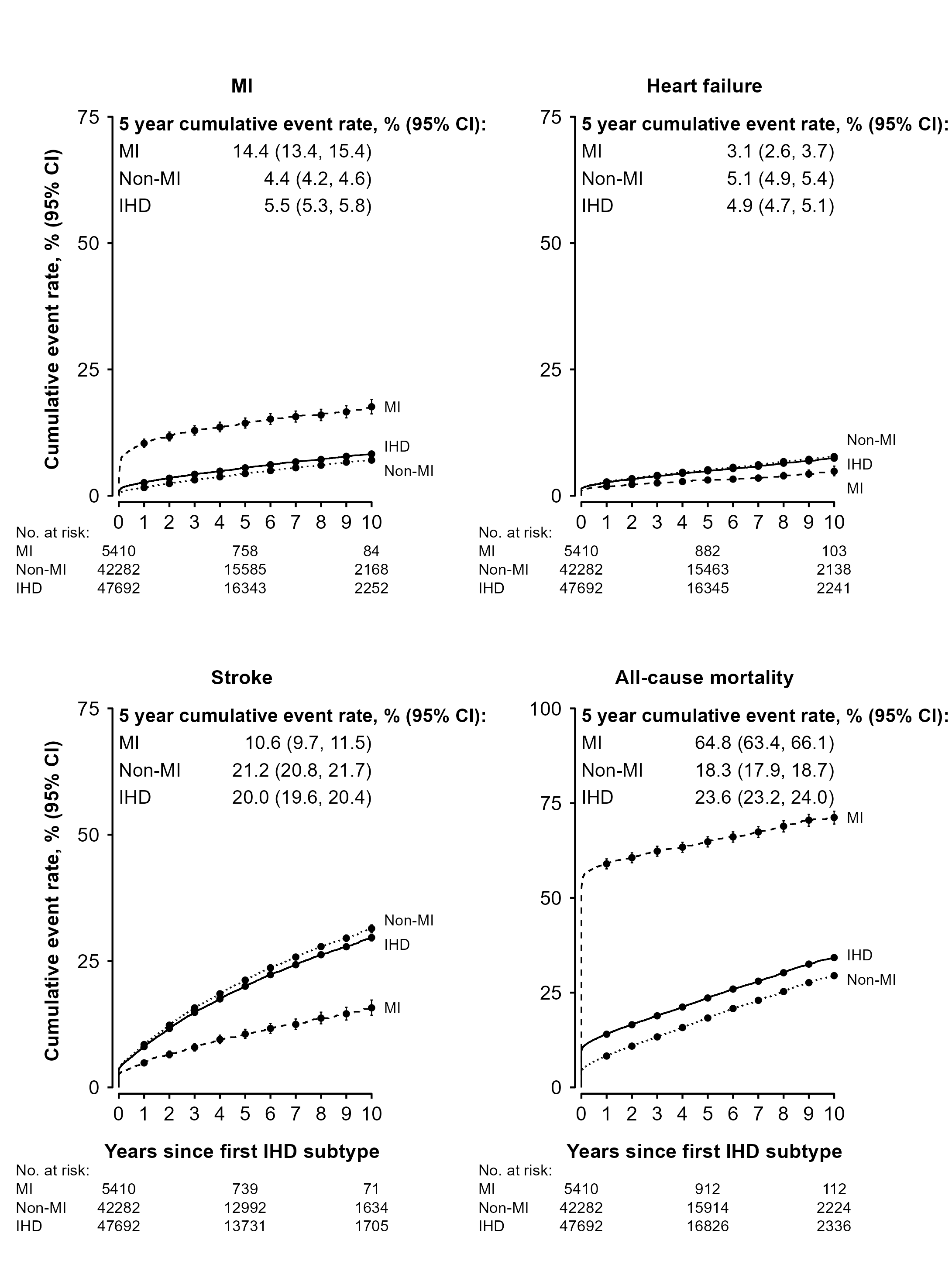


IHD = ischaemic heart disease; MI = myocardial infarction

Non-MI includes non-MI acute IHD and chronic IHD cases

# Figure S11: Estimated cumulative event rate of recurrent MI events, stroke, heart failure and all-cause mortality following first adjudicated event of different IHD types (combining before and after 28 days)


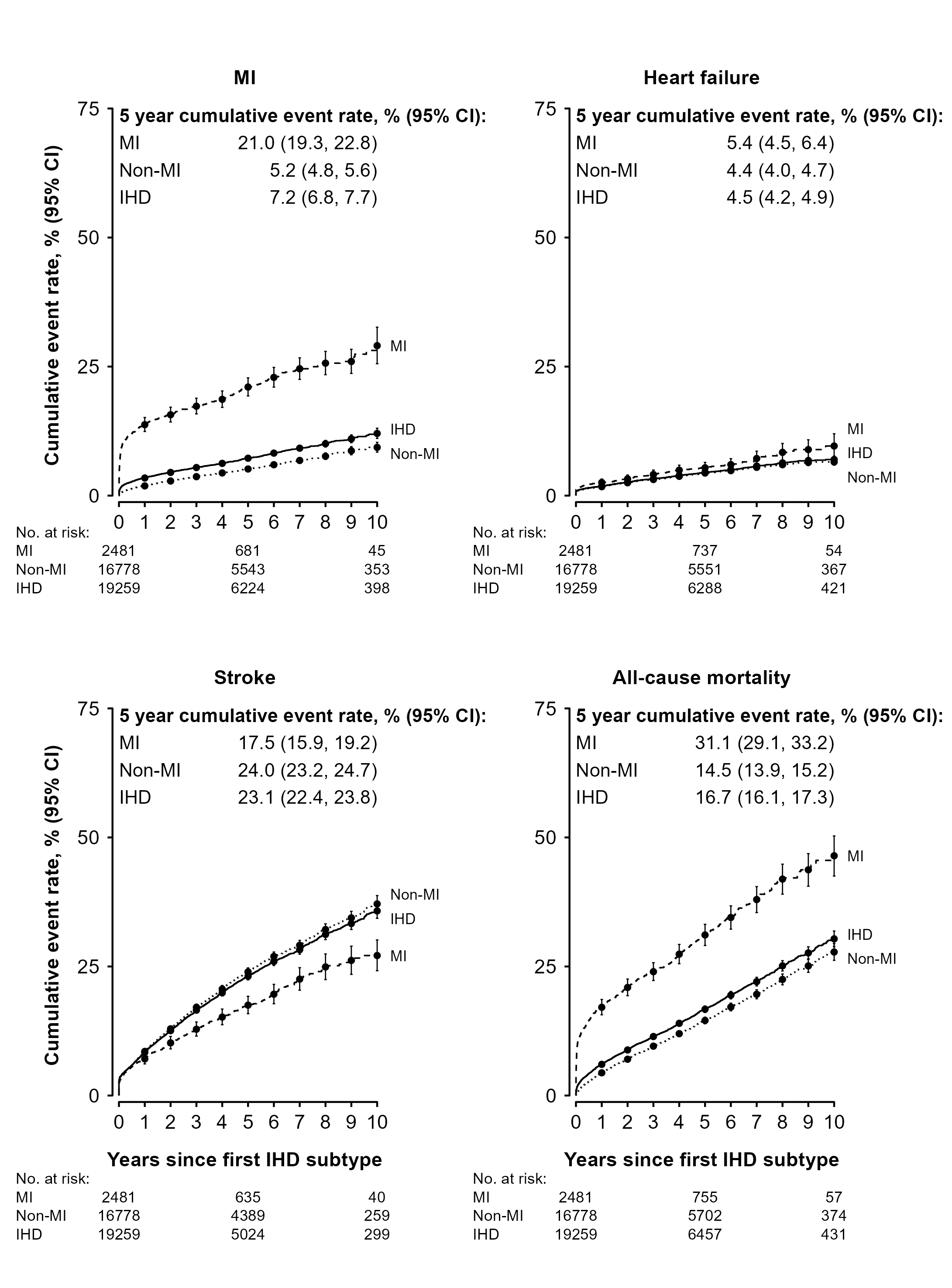


IHD = ischaemic heart disease; MI = myocardial infarction

Non-MI includes non-MI acute IHD and chronic IHD cases

# Figure S12: Estimated cumulative event rate of stroke types from 28 days after first adjudicated event of different IHD types


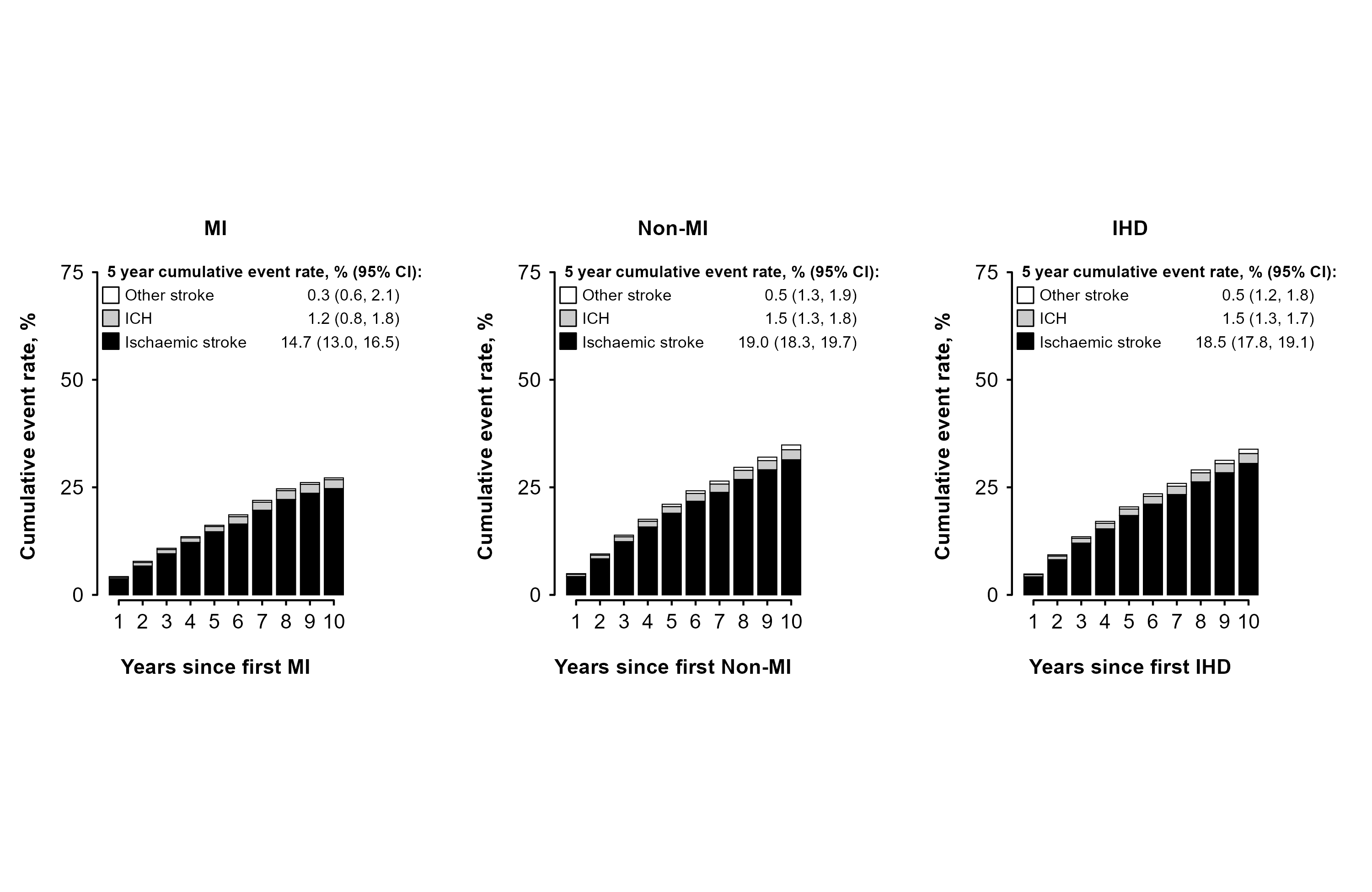


MI = myocardial infarction; ICH = intracerebral haemorrhage

Non-MI includes non-MI acute IHD and chronic IHD cases

# Figure S13: Estimated cumulative event rate of ischaemic heart disease and other cardiovascular disease from 28 days after first adjudicated event of different IHD types


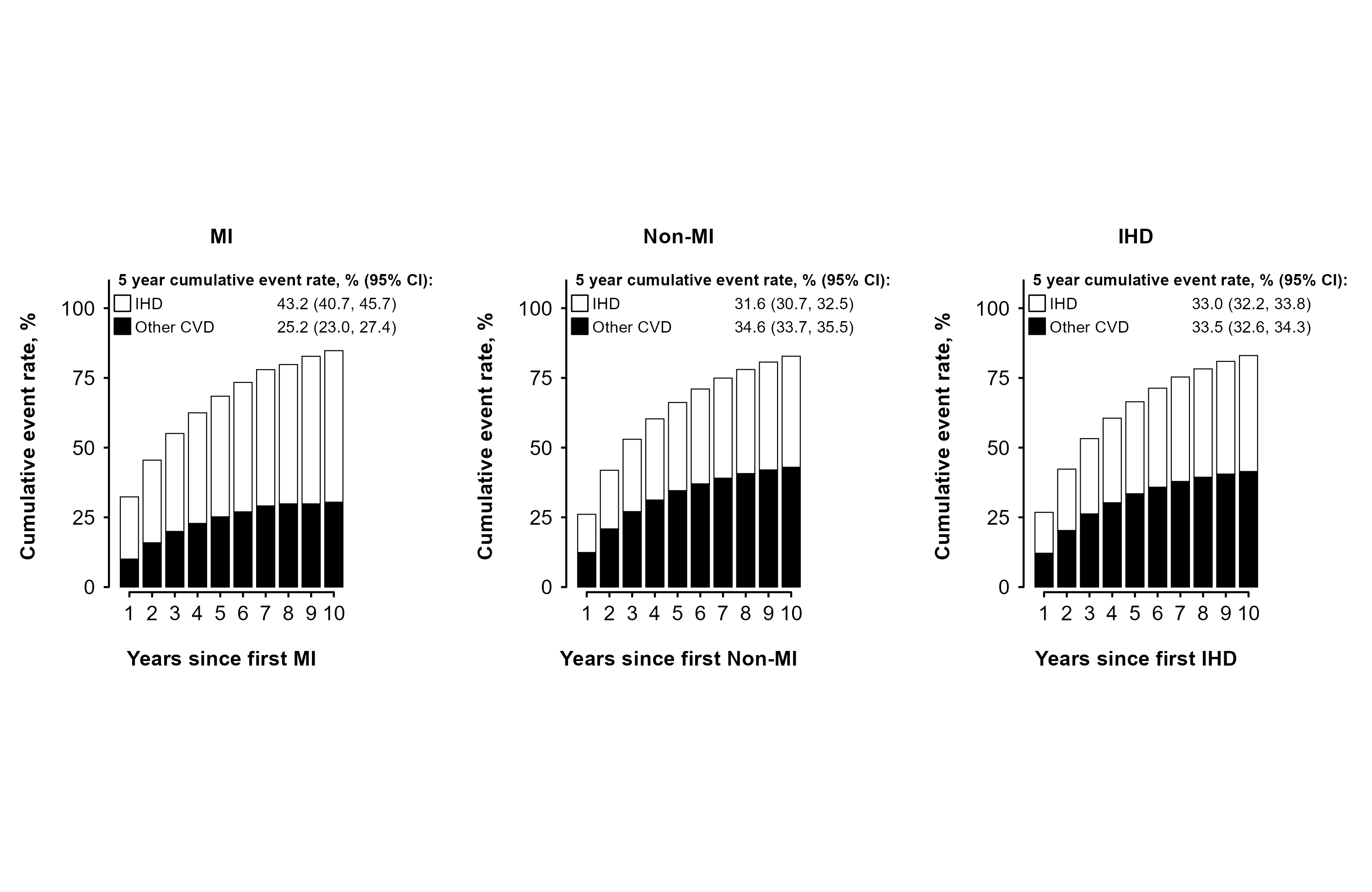


IHD = ischaemic heart disease; MI = myocardial infarction
